# A holistic understanding of literacy approaches at the environment-health-nexus: a scoping review and framework for health professionals

**DOI:** 10.3389/fpubh.2026.1960284

**Published:** 2026-10-19

**Authors:** Jennifer Kallenbach, Lydia Reismann, Ines Pfeffer, Carmen Jochem

**Affiliations:** 1 Faculty of Human Sciences, Department of Education, MSH Medical School Hamburg, University of Applied Sciences and Medical University, Hamburg, Germany; 2 Chair of Planetary & Public Health, University of Bayreuth, Bayreuth, Germany; 3 Institute of Cognitive and Affective Neuroscience (ICAN), MSH Medical School Hamburg, University of Applied Sciences and Medical University, Hamburg, Germany

**Keywords:** climate change, competence, concepts, environmental health, framework, health literacy, health professionals, planetary health

## Abstract

**Introduction:**

The health literacy of health professionals regarding planetary, climate, and environmental issues is crucial for protecting, promoting, and preserving human and planetary health, especially in the face of increasing environmental risks. To date, no systematic review of the existing literature has been conducted. This scoping review aimed to cluster and analyze scientific evidence on health literacy and competence concepts in the context of planetary, climate, and environmental health as applied to health professionals. Based on these findings, the aim was to develop a new, comprehensive, evidence-informed educational framework to promote professional planetary health literacy (PROF-PHL), to support the professional literacy development in addressing the growing challenges of planetary health.

**Methods:**

The scoping review was conducted in accordance with the JBI and PRISMA-ScR guidelines. Systematic searches were conducted in MEDLINE, CINAHL, and Web of Science, supplemented by manual searches. The results were evaluated using a combination of descriptive and deductive-inductive content analysis, in line with Kuckartz’s typification approach.

**Results:**

The review synthesized 17 health literacy and competence concepts from 28 existing studies on planetary, climate and environmental health. The review examined the concepts’ definitions, theoretical foundations, and environmental-health nexus, as well as their determinants across eight domains: knowledge, competences, attributes, competence dimensions, objectives, context, development processes, and proximal and distal antecedents. These were then integrated into an evidence-informed PROF-PHL framework through a conceptual synthesis. This framework embeds core competences across four dimensions—access, understand, appraise, and apply. The framework highlights the critical roles of systemic conditions, interprofessional education, and transformative action in advancing PROF-PHL.

**Discussion:**

The field of environment-related health literacy is characterized by conceptual heterogeneity and overlap, with a focus on individual competences. There has been a shift from one-sided environment-to-health concepts towards systemic, bidirectional approaches. Based on the available evidence, we have developed an integrative PROF-PHL framework that combines core competences, systemic determinants, and transformative action. This framework could support the integration of environment-related health competences into professional training programs, providing a foundation for initiatives that empower health professionals to promote planetary health in clinical, social, and political contexts. As a conceptual synthesis, however, the framework requires empirical validation.

**Systematic review registration:**

https://doi.org/10.17605/OSF.IO/HYNJR

## Background

Public and global health are increasingly impacted by environmental changes, with significant implications for physical, mental, and social well-being (1). In the face of climate change, the biodiversity crisis and rising levels of environmental pollution, the traditional, human-centered approach to health is inadequate (2). Planetary health offers a more comprehensive, systemic and forward-looking approach, emphasizing the inseparable link between human and ecological health (3). Planetary health is defined as ‘[…] the achievement of the highest attainable standard of health, wellbeing and equity worldwide through judicious attention to the human systems—political, economic, and social—that shape the future of humanity and the Earth’s natural systems that define the safe environment limits within which humanity can flourish’ (3). Planetary health aims to balance human well-being with environmental sustainability through multidisciplinary and cross-sector collaboration, fostering mindset and behavior change from the global to the local level (4). The Planetary health approach highlights the role of education and scientific innovation in addressing environmental health challenges and sustaining the foundations of human civilization (5).

Health systems are central to mitigating environment-related risks, safeguarding planetary health, and building the competences of health professionals to strengthen the resilience of both human and environmental systems in co-creating processes (6, 7). Accordingly, the Intergovernmental Panel on Climate Change (IPCC) 2023 and the World Health Organization (59) 2023 emphasize the importance of equipping health professionals with the competences to address environment-related health risks (8, 9). However, health professionals, defined according to the International Standard Classification of Occupational Health (ISCO) (58), often lack competences in the relationship between the environment and human health (6, 10). This restricts their ability to take transformative action within healthcare systems in a planetary health-promoting way (6, 11).

In this context, the professional health literacy among health professionals, is described as the motivation, knowledge, and competences required to find, understand, appraise, and apply relevant knowledge and information as part of a professional role (12). It appears to be of central importance for enabling health professionals to convey and communicate health- and illness-related knowledge and information in a way that enables patients to understand, appraise, and apply it to their own health decisions (12).

Emerging, environment-related health literacy concepts such as planetary health literacy (13), climate health literacy (11), One Health literacy (14) and environmental health literacy (15, 16) aim to bridge this gap. All these concepts highlight the link between environmental and human health, and aim to promote informed, health-oriented decisions and behavior—with varying emphasis on different environmental aspects (13, 17). By considering environmental determinants and health-related competences, these concepts have helped to develop a more comprehensive understanding of health literacy and its competences required to understand, and act on the interconnections between human and planetary health (13). In particular, the concept of planetary health literacy, one of the most recent environment-related health literacy concepts (18), aims to foster an integrated understanding of ecosystems and human health (13). Despite the similarities between the various concepts, the field remains fragmented due to the absence of a consistent theoretical framework for distinguishing between these overlapping health literacy concepts (18, 19). Although Jochem et al. (13) provide an initial overview of the definitions of selected environment-related health literacy concepts, so far, the key determinants shaping the development of environment-related health literacy, as well as the differences and similarities between existing concepts have not been comprehensively summarized.

In light of the growing environmental threats to health and the important role of educational institutions in shaping our future, it is crucial to establish an educational framework that encompasses existing environment-related health literacy approaches, and enables targeted competence development to support planetary health (5). Such a framework, as a holistic concept, has the potential to empower health professionals to contribute to healthier, more sustainable behaviors, build climate-resilient health systems, and advance a systemic response to today’s polycrisis (20). It may empower learners to take transdisciplinary, mutually reinforcing action to protect, and restore planetary health, and to achieve the Sustainable Development Goals (5).

To gain a comprehensive and systematic overview of existing environment-related health literacy and competence concepts, and their determinants, and to develop an initial, evidence-informed educational framework that combines environment-related and professional health literacy approaches under the umbrella of a new concept of professional planetary health literacy (PROF-PHL), this study pursues the following objectives: (1) Comprehensively summarize the scientific evidence on environment-related health literacy concepts as applied to health professionals; (2) Develop a conceptual evidence-informed framework of PROF-PHL for health professionals; and (3) Foster the scientific discourse on PROF-PHL of health professionals in the context of planetary health challenges.

Thereby the scoping review aims to answer the following research questions:

- Which concepts describe environment-related health literacy or competences applicable to health professionals?
- What are the key determinants of these concepts that influence health professionals’ environment-related health literacy and competences?
- Which similarities and differences exist among these concepts?

- What might a holistic, evidence-informed framework of PROF-PHL for health professionals look like?

## Method

This scoping review was conducted following the JBI methodology for scoping reviews (21, 22). The study protocol was registered in the OSF repository (https://doi.org/10.17605/OSF.IO/HYNJR). Reporting adhered to the checklist ‘Preferred Reporting Items for Scoping Reviews’ (PRISMA-ScR) to ensure transparency and quality (Additional Table 1 in the Supplementary material).

### Search strategy and selection criteria

A three-step search strategy was applied. An initial limited search in MEDLINE and CINAHL via EBSCO was performed to refine keywords. Search strings were developed using identified keywords and MeSH terms, with input from the ISCO classification (65). The final search was conducted on 31 January 2025 across MEDLINE, CINAHL, and Web of Science, supplemented by grey literature searches via Google Scholar and institutional platforms (ScienceDirect, ClinicalKey, Springer Nature). The databases were selected for their comprehensive, peer-reviewed content. Search terms included combinations of ‘health professionals’, ‘planetary health’, ‘environmental health’, ‘climate change’, ‘health literacy’, ‘competence’, and related synonyms. Search strategies were reviewed in an iterative process by co-authors and are detailed for each database in the Additional Table 2 in the Supplementary material.

Using the Population-Concept-Context (PCC) Framework (23), the inclusion criteria encompass studies on concepts of health literacy or competences among health professionals, integrating planetary, climate, or environmental aspects, published up to January 31, 2025, in English or German. Exclusion criteria apply to studies without health-related components, lacking conceptual frameworks, or unrelated to planetary, climate, or environmental health, as well as media, blogs, editorials, and publications in other languages. The final set of inclusion and exclusion criteria, and their explanations (Additional Table 3 in the Supplementary material) was developed through an iterative refinement process (24).

### Data extraction and synthesis

All citations were imported directly from the database, while the grey literature studies were added manually by JK to Citavi^®^ (version 7.1.0.1). After verifying that the data had been correctly transferred by JK, the citations were exported directly into Rayyan® software for screening (25). Two reviewers (JK, LR) independently screened duplicates, titles, abstracts, and full texts against the eligibility criteria. Discrepancies were resolved by common consent by the third reviewer (CJ). Additional Table 4 in the Supplementary material lists all studies excluded by the authors, together with the reasons for their exclusion. The team of authors then exported the full-text articles that were included directly from Rayyan® as an Excel® document and prepared them for content analysis. To this end, the authors extracted descriptive data to provide an overview of the main characteristics of environment-related health literacy and competence concepts. This was based on the following criteria: (a) references, (b) country, (c) affiliation/discipline, (d) methodological approach, (e) target group, (f) environmental focus, (g) literacy/competence concept, and (h) study objectives (Additional Table 5 in the Supplementary material), as well as the definitions and determinants of the concepts (Additional Table 6 in the Supplementary material). To answer all the research questions in a differentiated way, the authors carried out a deductive-inductive content analysis following the typification approach of qualitative content analysis (61). First, JK constructed the feature space for typification, i.e., the identification of the relevant determinants of all concepts, based on the health literacy typology developed by (63). This typology is based on a systematic review and synthesis of definitions and models (63). This provided a theoretically sound and empirically validated basis for identifying key determinants (e.g., antecedents, context, objectives). Secondly, JK and LR systematically and deductively grouped the included studies according to their conceptual determinants, using (63) predefined typological scheme. These typologies were then further developed using an inductive approach: determinants that could not be categorized were assigned to a new typology and determinants to which no concept could be assigned were discarded. However, only the predefined ‘Information’ typology (63) yielded no significant content. Instead, a new typology ‘Knowledge’, was derived. This deductive-inductive approach proved effective in capturing all determinants. It enabled the authors to identify recurring patterns and form clearly defined typological categories. The final set of typologies was as follows: (1) knowledge; (2) competences, skills and abilities; (3) competence attributes; (4) competence dimensions; (5) actions and outcomes; (6) context; (7) development process; and (8) individual and contextual antecedents (Additional Table 7 in the Supplementary material). Thirdly, each study was assigned to the typological scheme based on its content. To this end, JK extracted the data for the first five studies using the defined typological scheme. LR then checked the extracted data, after which CJ approved the final decisions to ensure inter-rater reliability. This process is documented in the Additional Table 7 in the Supplementary material. The final step involved synthetically integrating the typology scheme into a framework. This was achieved through cross-study analysis and iterative author discussions, ultimately leading to the development of the PROF-PHL framework.

## Results

Electronic database searches retrieved 1,599 references (MEDLINE: 406; CINAHL: 514; Web of Science: 679). A further 99 references were identified through manual searches of Google Scholar and publisher websites (e.g., Springer Nature, ScienceDirect and ClinicalKey), as well as through citation tracking. This led to a total number of records of 1,698. After removing duplicates, 1,399 titles and abstracts were screened. Four articles were not available in full text. The remaining 220 studies (182 from the database and 38 from other sources) underwent full-text screening to determine their eligibility. Of these, 192 were subsequently excluded, with the reasons indicated in Additional Table 4 in the Supplementary material. Finally, 28 articles were included in the scoping review. The study selection process is outlined in the PRISMA-ScR flow diagram in Figure 1 (26).

**Figure 1:**
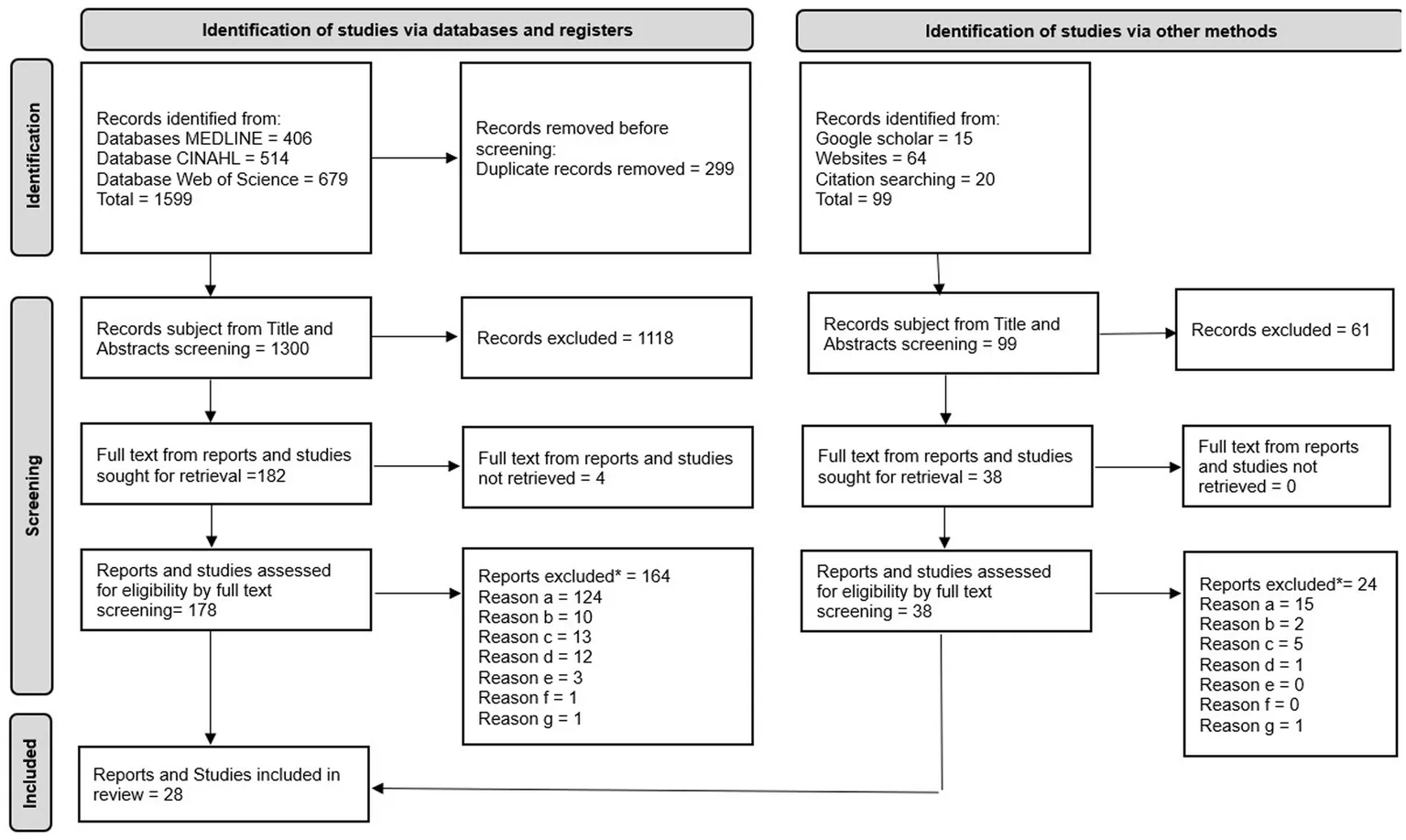
Review process in a PRISMA-ScR flow diagram. * Reasons (a) no literacy or competence concept; (b) no environmental focus; (c) wrong population; (d) no health aspect linking literacy or competence; (e) foreign language; (f) wrong type of source; (g) duplicate in other language. Source: Page MJ, et al., (26).

### Main characteristics

Additional Table 5 in the Supplementary material gives an overview of the descriptive analyzed main characteristics of the 28 included articles. The most important characteristics of all included studies in relation to the research questions are summarized in Table 1. The included studies are characterized by an interdisciplinary approach in terms of researcher collaboration, scientific disciplines, and a wide range of different methodological research designs, dominated by research institutions from the Global North. The environmental perspective taken in the studies is also diverse, with most studies pursuing the planetary health approach (*n* = 8), the environmental health approach (*n* = 7) or the climate health approach (*n* = 9). In addition, 17 different environment-related health literacy and competence concepts were identified.

**Table 1:** Cluster of key conceptual determinants of the included studies.

Authorship and collaboration	Authored teams (*n* = 26), international collaboration (*n* = 8), interdisciplinary collaboration (*n* = 21)
Disciplinary background	public health, medicine, environmental, climate and global health sciences, and educational research, nursing, physiotherapy, psychology, planetary health, quality and safety, technology, social work, sport, nutrition, communication, economics, and policy sciences
Methodological approaches	Qualitative (*n* = 4), quantitative (*n* = 4), mixed methods (*n* = 6), and conceptual approaches (*n* = 14)
Target group	Health professionals in general (*n* = 10), nursing (*n* = 2), medicine (*n* = 2), psychotherapy (*n* = 1), environmental health professionals (*n* = 1), and rural and remote health professionals (*n* = 1), students or learners (*n* = 15), from health-related disciplines such as public health (*n* = 1), nutrition (*n* = 2), medicine (*n* = 2), nursing (*n* = 4), teachers and trainers (*n* = 8), policy makers (*n* = 1), health policy, governance and leadership in One Health (*n* = 1), public health professionals (*n* = 1) and environmental scientists (*n* = 2), patients (*n* = 1), general public (*n* = 7)
Environmental focus	Planetary health (*n* = 8), environmental health (*n* = 7), climate change (*n* = 9), one health (*n* = 1), sustainable diets in relation to human and environmental health (*n* = 1), intersection of climate and environmental health (*n* = 1), ecological perspectives on health and care (*n* = 1)
Literacy and competence concepts	(a) Climate approach: climate health literacy (*n* = 2), climate-specific health literacy (*n* = 2), climate change literacy (*n* = 1), health -related climate literacy (*n* = 2) (b) Climate- und environmental approach: climate and environmental health competences (*n* = 1), climate, environmental change, and health competencies (*n* = 1) (c) Environmental approach: environmental health literacy (*n* = 6), environmental health competences (*n* = 1) (d) Planetary approach: planetary health literacy (*n* = 2), planetary health learning outcomes and competences (*n* = 2), planetary health knowledges and skills (*n* = 1), planetary health diet literacy (*n* = 1) (e) Other approaches: sustainable food literacy (*n* = 1), eco-medical literacy (*n* = 2), ecological care (*n* = 1), one health literacy and one health governance (*n* = 1), carbon health literacy (*n* = 1)

### Target groups

As seen in Additional Table 5 in the Supplementary material, the descriptive analyzed studies showed a broad understanding of health professionals. In addition to traditional health professionals such as medicals and nurses, the analyzed studies included professionals from public health, nutrition, environmental sciences, health policy, students, educators, and the broader public. As can be seen in Additional Tables 5, 8 in the Supplementary material, 14 of the 28 included studies aimed to target health professionals. Six of these also included students and trainees (27–32). Four studies explicitly targeted only health professional students or trainees (33–36). The remaining 10 studies targeted the general public and students or occupational groups working alongside health professionals in transdisciplinary settings (13–15, 28, 37–41). In order to analyze the results in greater detail for each target group, they are categorized according to the group involved:

- concepts developed directly among health professionals;
- concepts developed among health professional students or trainees;
- concepts originally developed among the general population or other non-professional groups.

This is because these three groups do not provide the same level of evidential support for a health professional competence framework, and their potential transferability to health professionals must be considered.

### Definitions, conceptual background, and determinants

Additional Table 6 in the Supplementary material provides a comprehensive overview of the definitions, theoretical foundations, and determinants associated with all concepts related to environmental health literacy and competence, which are summarized below.

#### Concepts developed directly among health professionals

The scoping review identified eight health literacy and competence concepts designed specifically for health professionals: climate-specific health literacy (6, 11); environmental literacy in relation to climate change and eco-medical literacy (42); health-related climate literacy (27, 43); climate, environmental change, and health competences (44); planetary health literacy (20); climate health literacy (30, 45); and carbon health literacy (46). All concepts are defined as multidimensional integrations of determinants, e.g., knowledge, skills, attitudes, and competences related to the environmental-health nexus. Most incorporate the four health literacy competences: accessing, understanding, appraising, and applying environmental health information (20, 27, 30, 42, 43, 45, 46). The environmental-health focus varies by conceptual foundation. For example, carbon health literacy emphasizes making decisions based on greenhouse gas emissions (46); Climate health literacy addresses the complex interplay between climate change and human health (27, 30, 43, 45); Planetary health literacy pursues the objectives to make judgements and take decisions regarding planetary health, across societies and for health-promoting, sustainable, and transformative actions (20); Eco-medical literacy focuses on the ecological impact on the delivery of medical services (42); ‘environmental literacy for climate change’ emphasizes ecosystem self-sustainability (42); and ‘climate, environmental change, and health competences’ analyze the intersections between climate, the environment, and health (44). While many concepts are grounded in established frameworks, such as Planetary Health (3), and the Global Competency Framework for Universal Health Coverage (44), others emerge as topic-specific constructs without reference to an underlying theoretical or conceptual approach, particularly with regard to climate change and health, e.g., carbon health literacy (46), and climate-specific health literacy (6). These approaches consider developing competence to be a multidimensional task involving various stakeholders and settings. Jochem et al. (20), for example, explain that planetary health literacy encompasses not only the individual level, but also the societal and global levels.

#### Concepts developed among health professional students or trainees

In addition, eight studies that aimed to promote health literacy and competence among students and/or trainees in the healthcare sector were identified. Six of these studies were specifically designed for medical and nursing students. These studies primarily focus on approaches that address competences and learning objectives in specific healthcare settings (e.g., care and medicine): ecological care (32); eco-medical literacy (31); environmental health competences (36); planetary health knowledges and skills (35); planetary health learning outcomes and competences (29, 34); and climate change health literacy (28, 33). These concepts also incorporate multidimensional determinants within their definitions of competence and health literacy. For example, Moghbeli et al. (32) describe ecological care as the integration of environmental sensitivity, knowledge, skills, attitudes, values, responsibilities and active engagement. This enables nurses to make informed and responsible decisions that promote environmental sustainability (32). Of all the concepts, only Lopez-Medina et al. (36) do not provide a definition of their area of expertise in environmental health. The four health literacy competences—access, understand, appraise, and apply—can be found in some of the definitions described here (28, 31). For instance, Maxwell et al. (31) defines eco-medical literacy as the capacity to acquire, comprehend, combine, and utilize information concerning the environmental health impacts of climate change to deliver, and enhance medical services. The concepts predominantly emphasize the interdependent development of different actors, and reference systems in their understanding of competence and health literacy (31, 32, 34). As described by Maxwell et al. (31), eco-health approaches recognize the interdependence of human, animal, and ecosystem health. They are aimed at developing transdisciplinary, systems-focused collaborations that improve health through social stability and ecological sustainability (31).

#### Concepts originally developed among the general population or other non-professional groups

The remaining 10 studies included in the analysis are aimed at the general public, teachers, learners, professionals working in environmental or health-related fields, and policymakers. The following concepts of health literacy and competence were presented in these studies: planetary health diet literacy (41); sustainable food literacy (47); environmental health literacy (15, 37–40, 48); planetary health literacy (13); and One Health literacy (14). Notably, six of the 10 studies relate to environmental health literacy. Furthermore, the environmental health literacy approach is the oldest concept included. The definitions described a broad consideration of the various determinants that influence health literacy and competence development. For example, Lichtveld et al. (39) note that environmental health literacy encompasses a wide range of knowledge, competences, attitudes, and behaviors. The environment-health nexus also varies depending on the concept. While the two nutrition-related approaches aim to develop the ability to make sustainable food choices (41, 47), environmental health literacy approaches focus on applying a nuanced understanding of environmental health risks, exposures, impacts, and strategies to reduce harmful effects and promote health (15, 37–40, 48). The One Health literacy approach addresses health issues within the interconnected human, agricultural, and environmental sectors (14). The four core competences (access, understand, appraise, and apply) can be identified in five concepts as a developmental process of competence (13–15, 38, 41). For example, Finn and O’Fallon (15) describe their health literacy concept as a broad spectrum of skills and competences that people need in order to seek out, understand, evaluate, and use environmental health information to make informed decisions, reduce health risks, improve quality of life and protect the environment. Environmental health literacy approaches emphasize that their understanding of competence development starts with the individual. Lindsey et al. (40) state that environmental health literacy fundamentals are achieved when an individuum understands the link between environmental exposures and health effects. In contrast, planetary health literacy, sustainable food literacy, and One Health literacy approaches emphasize the inter- and transdisciplinary nature of competence development. Accordingly, Blankart et al. (14) describe competence as something that considers the interconnection between the fields of human, agricultural, and environmental sciences, working towards a common goal in a transdisciplinary manner.

## Discussion

Based on a descriptive content analysis of the included studies, we compared conceptual similarities and differences. Through deductive-inductive content analysis, following the typification approach (61), we synthesized core determinants into a determinants cluster and developed an initial, comprehensive, evidence-informed framework for professional planetary health literacy. This new framework is presented alongside a definition.

### Similarities and differences

The similarities and differences between the concepts are presented across the various target groups, since no group-specific differences were observed. However, it is recognized that the generalizability of the findings to health professionals may be limited. The most significant similarities and differences were found in the following three areas: the environment-health nexus, target groups and settings, and definitions.

#### Environment-health nexus: from uni- to bidirectional understanding

Analysis of various environment-related health literacy and competence concepts reveals significant similarities and crucial differences in understanding the relationship between environmental and human health. While all approaches recognize the profound impact of ecological systems on health, concepts related to the environmental health approach frame the environment as a unidirectional influence on health (40). In contrast, other system-based concepts, such as ecological care (32), planetary health (13), and One Health (14), emphasize the reciprocal interdependence of healthy environments and ecosystems. These are not only prerequisites for health, but are also influenced by healthcare activities (32). In their ‘climate, environmental change and health competencies’ health worker framework, Jagals and Ebi (44) identify climate change and environmental degradation as key factors that exacerbate health inequalities, and impact the social and environmental determinants of health (44). The ecological care approach goes one step further, taking a systemic view of the interactions between living beings, and their physical and social environments (32). The One Health approach emphasizes the interconnectedness of human, animal, and environmental health, advocating for interdisciplinary collaboration (14). The planetary health approach integrates these perspectives into a transdisciplinary, holistic view that considers the entire socio-ecological basis of human health. Particular emphasis is placed on addressing global challenges such as climate change, biodiversity loss, and environmental pollution through adaptation and mitigation (13, 34). Many of the foundational concepts stem from environmental health literacy, which predominantly focuses on unidirectional environmental influences on health (15, 37, 39). More recent concepts such as planetary health literacy and One Health literacy advance a bidirectional understanding that highlights the inseparability of human and planetary health (13, 14, 32).

#### Definitions and determinants: heterogeneity of terminology, ambiguity, and overlap

The content analysis revealed considerable heterogeneity of terminology, ambiguity, and overlap among environment-related health literacy concepts. While the variety of health literacy concepts, e.g., planetary health literacy (13), environmental health literacy (40), climate health literacy (28), ecological care (32) reflects the richness of interdisciplinary influences, it also reveals substantial overlaps and lack of clarity, e.g., the terminologies of health-related climate literacy (27), climate-specific health literacy (11), climate and health literacy (30), and climate change literacy (33). While most studies provide definitions for their concepts (Additional Table 6 in the Supplementary material), inconsistencies exist within and across publications. For instance, the six environmental health literacy concepts (15, 37–40, 48) are all based on different theoretical foundations, each involving different skills and competences. For example, Binder et al. (48) describe their approach as ‘capturing the continuum between knowledge of environmental impacts on public health, and the skills and decisions needed to take health-protective actions’ (48), whereas Fiore et al. (38) describe environmental health literacy as ‘developing the wide range of skills and competences that people need to seek out, comprehend, evaluate, and use environmental health information to make informed choices, reduce health risks, improve quality of life and protect the environment’ (38). Some terms are used interchangeably without clarification (climate literacy vs. climate change literacy) (33). In a few cases, definitions remain vague or incomplete (e.g., environmental health literacy requires a ‘broad range of skills and competencies’ (38). This makes it difficult to operationalize and integrate the concepts in both research and practice. This observation is consistent with the findings of previous studies, both in terms of the limited number of studies and the heterogeneity observed (18, 19, 49, 50), and underscores the need for standardized terminology and transparent documentation in future research (13, 18). Despite these inconsistencies, most definitions share core determinants and competences. Health literacy in the environmental context is generally understood as the knowledge, skills, attitudes and competences needed to access, understand, appraise, and apply information to make judgments, decisions, and actions in everyday life with regard to health care, disease prevention, and health promotion, and to sustainably balance the health and quality of life of humans, animals, and the environment (13, 31, 32, 39).

#### Target groups and settings: from an individual to a systemic perspective

Analysis of the levels of target groups and settings reveals clear distinctions between individual, professional, and political actors, which are essential for developing and promoting health literacy related to the environment (27). Earlier concepts, such as environmental health literacy approach, primarily targeted individual behavioral changes and personal environmental attitudes (48). These concepts emphasized the functional level of environment-related health literacy while neglecting transformative and systemic competences, as well as key contextual factors that influence collaborative development (31, 33). Newer concepts, such as health-related climate literacy (27), planetary health literacy (13), and ecological care (32), represent a paradigm shift towards a more systemic understanding. Rather than emphasizing individual competences alone, these approaches consider a more holistic systems perspective that incorporates structural determinants of planetary health, and the complex interdependencies among individual, social, and ecological actors as part of a reciprocal network.

The analyzed concepts demonstrated a broad understanding of health professionals, extending beyond the confines of traditional medical disciplines. This is evident from the fact that several studies have involved a broad group of stakeholders, including health professionals, in developing and promoting health literacy and competences (20, 27, 28, 30). This highlights the transdisciplinary and socially embedded nature of environment-related health literacy concepts. However, it is important to note that concepts developed directly among health professionals provide the most robust evidence foundation, particularly regarding professional competences, systemic determinants, and transformative action, student-, and trainee-based concepts offer valuable insights into foundational learning but require further development to reflect professional practice, and general population-based concepts serve as important starting points but must be critically adapted to ensure relevance to health professionals’ roles and responsibilities. The comprehensive spectrum of environment-related health literacy concepts applicable to health professionals demonstrates that an environment-related health literacy framework cannot be limited to individual health professional groups, but requires the broad, systemic participation of health and environmental stakeholders (27). At the same time, health professionals play a crucial role in bringing together the various stakeholders, and implementing measures to protect planetary health (6, 34).

Furthermore, the research field is shaped by interdisciplinary perspectives, particularly those from Global North science. Rather than being used as a geographical classification, the Global North–South framework is employed as a critical tool for reflecting on power, knowledge hierarchies and structural inequalities. The dominance of the Global North in literature is seen as an indication of epistemological and institutional power relations (62).

### Cluster of determinants

Using a deductive-inductive typification approach (61), we clustered and analyzed the core determinants of the included concepts. The Additional Table 7 in the Supplementary material summarizes all the identified determinants for the cluster.

#### Development process

Environment-related health literacy and competence development is conceptualized as a dynamic, lifelong process (13), influenced by personal experiences (32), professional demands (28), and societal change (28). Health professionals evolve along a continuum from basic knowledge toward expert-level competences on which they can act professionally (44). Progress along this continuum requires not only education and training (44) but also enabling environments and institutional support (32).

#### Individual and contextual antecedents

These contextual factors can be described as antecedents, which can be divided into personal (proximal) and organizational (distal) factors (32). To translate health literacy into environmentally and health-promoting behavior, Lindsey et al. (40), and Moghbeli et al. (32) suggest that a wide range of proximal and distal influences must be considered (32, 40). Proximal antecedents, e.g., age (48), values and beliefs (32), self-efficacy (37), attitudes (39), and distal factors, e.g., cultural context, communication modalities (15), geography (48), act both as barriers, and facilitators in promoting health literacy and competences (32). Health literacy, in turn, can influence these determinants, e.g., by enhancing resource mobilization or reducing system-level resistance (13, 20).

#### Knowledge

According to Jagals and Ebi’s (44) ‘climate, environmental change and health competencies’, and Binder et al.’s (48) environmental health literacy concept, knowledge is fundamental to developing environment-related health literacy and is therefore a vital resource. They consider knowledge to be both a subjective and objective dimension of health literacy, referring to ‘knowledge of facts’, ‘willingness to make decisions’, and ‘expectations of effectiveness’ of knowledge (44, 48). Factual knowledge objectively influences decisions to take protective action (48). According to Finn and O’Fallon, people need to understand how environmental health risks affect health, how to avoid these risks, and the role of scientific research in understanding these relationships (15). Furthermore, Binder et al. (48) emphasized that individuals must subjectively perceive their ability to make informed decisions. Finn and O’Fallon believe that, once people feel they have the necessary information, and skills to change their behavior, they need to be convinced that their actions will protect them (15).

#### Competence dimensions and attributes

The content analysis identified four core competence dimensions essential for developing environment-related health literacy among health professionals (13–15, 27, 28, 30, 31, 39–42, 46), adapted from the planetary health literacy approach of Jochem et al. (13):

1. *Access:* The ability to locate and retrieve relevant planetary health-related information from diverse sources.
2. *Understand:* The capacity to process and comprehend complex interdependencies between environmental systems and human health.
3. *Appraise:* The critical evaluation of information, assessing relevance, reliability, and implications for decision-making regarding planetary health.
4. *Apply:* The translation of knowledge into concrete, sustainable, and health-promoting actions.

In line with Fiore et al. (38), the above four competence dimensions align with the three health literacy levels:

1. *Functional*: Basic competences to understand and use information with an emphasis on the importance of taking action to address planetary health risks.
2. *Interactive*: Competences that enable health professionals to understand and appraise statements about planetary risks related to human health and natural systems, as well as communicate these risks across multidisciplinary sectors.
3. *Critical*: Competences that enable health professionals to critically reflect on and evaluate health information related to planetary impacts, ultimately allowing them to act in a transformative and sustainable manner.

Each competence dimension is associated with a variety of cognitive, emotional, and behavioral attributes, relating to critical reasoning, collaboration, leadership, advocacy, ethical practice, and systems thinking (15, 29, 44). These attributes, which are specific verbs, can be used to describe each competence dimension explicitly (32).

#### Actions and outcomes

The overarching outcome objectives of the environment-related health literacy concepts for health professionals include: (1) Promoting planetary and population health (13, 29, 34, 35); (2) Adopting and supporting climate- and environment-promoting healthcare practices (6, 47); (3) Empowering individuals and communities to act in an environmental promoting manner (14, 48); (4) Enhancing system-level sustainability and resilience (14, 37), and (5) Providing high-quality of holistic care (6, 32, 35). The central role of health professionals—as change-agents for health—is to support the community in proactively managing their health (36, 41). Through collective action, these agents can exert a transformative influence on environment-related health literacy, encompassing both public health and global health concerns (13, 36). In this manner, they could contribute to achieving positive social tipping points toward a civilizational shift toward global sustainability (34).

#### Context

At the same time, the outcomes and actions show that the development of environment-related health literacy is context-dependent and that possible influences of the respective settings on health literacy development must be taken into account (27). These include: the clinical practice of health professionals (6, 11, 45), public health settings (13, 47), policy and governance structures (14, 48), and educational programs (28, 29, 37). Special attention is required in underserved, rural, and indigenous health settings (42).

### Integrative PROF-PHL definition and framework for health professionals

Based on the synthesized results of the determinants cluster, the authors proposed an integrative evidence-informed PROF-PHL definition and framework for health professionals (Figure 2). The core determinants were combined into a new, comprehensive definition of PROF-PHL, which aims to enable uniform terminology in its application to health professionals’ education:

**Figure 2:**
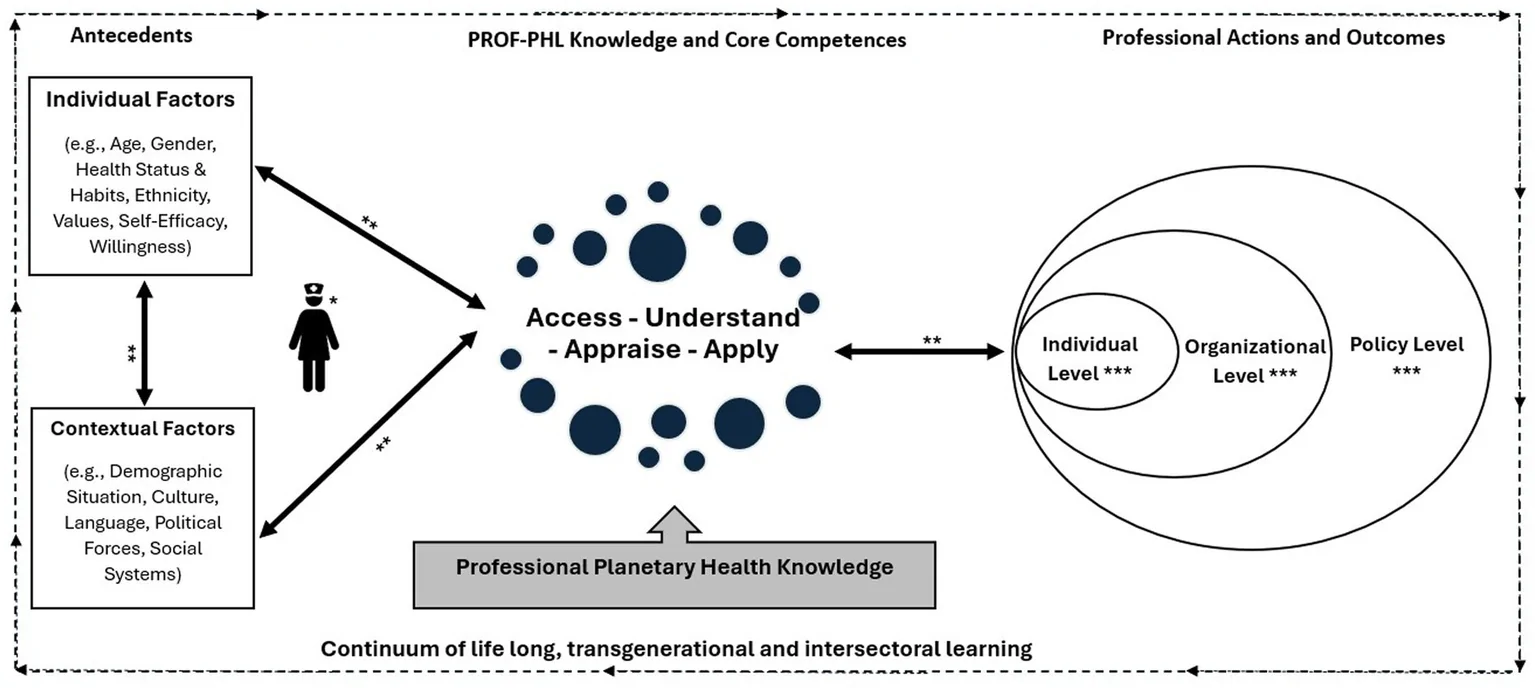
PROF-PHL framework – Integrative conceptual framework of Professional Planetary Health Literacy for health professionals (own representation). *Health professional. **The arrows represent the reciprocal, feedback-driven relationships that exist between all levels, reflecting the systemic and iterative nature of developing professional health literacy competences. ***What constitutes a professional action or outcome at each stage. For example: Individual level: 'Critical reflection on environmental impacts in clinical decision-making'; Organizational level: 'Implementation of sustainable clinical protocols'; Policy level: 'Advocacy for environmentally friendly health policy'.

*Professional planetary health literacy describes the ability of health professionals to access, understand and appraise planetary health-related information in order to derive decisions for health-promoting, sustainable, and transformative professional action, to communicate professionally, and to apply this information appropriately in their everyday work.*

PROF-PHL is an integrative concept combining environment-related health literacy and competence concepts under the umbrella of planetary health literacy. The umbrella of planetary health literacy was chosen for a more comprehensive systemic perspective, which enshrines the interdependence of human health and healthy ecosystems as a central principle (18).

To help illustrate how the results from the evidence synthesis have been incorporated into the PROF-PHL framework, the authors created an ‘Evidence-to-Framework Matrix’ (Additional Table 9 in the Supplementary material). This matrix shows how the framework’s components and underlying concepts are derived from the literature, adapted or synthesized by the authors. This makes the development process more transparent and traceable. The individual components of the PROF-PHL framework are explained in more detail below:

According to Lynch et al. (46), ‘knowledge’ is fundamental to developing PROF-PHL. Health professionals can use their knowledge to influence healthcare managers, promote preventive healthcare, and reduce low-value care in order to drive change (46). Jacobsen et al. (29) have defined eight knowledge domains that are relevant to courses and curricula in planetary health. These eight domains are: Earth system changes, ecological systems, impacts on human health, risk assessment, governance, interventions, ethics, and communication. Together, these domains provide learners with essential knowledge about the science, interventions, and communication relating to planetary health (29), informing our understanding of ‘professional planetary health knowledge’ within the PROF-PHL framework.

The identified ‘antecedents’ are defined in our framework as factors that could potentially influence the development of PROF-PHL. These include individual (proximal) antecedents from the health professional themselves, such as motivation and awareness, as well as contextual (distal) antecedents from their environment, including economic, social and cultural factors (32, 39, 40). The development of competences depends not only on an individual’s knowledge, but also on factors such as self-efficacy, emotional resilience, and social support. These factors are strong predictors of competence application in practice (39, 40). Furthermore, distal or contextual antecedents can act as systemic barriers that prevent the effective exercise of competence. Even if health professionals have the necessary knowledge and competences, they may be unable to act due to a lack of time or resources, a lack of institutional support, cultural norms that discourage critical thinking, or economic interests that hinder implementation (32). PROF-PHL is not merely an individual characteristic, but also a result of systems. Therefore, the inclusion of contextual antecedents in our framework is consistent with the principles of planetary and systemic health research, which advocate interdisciplinary, context-specific and transformative approaches (3, 51).

As the term ‘competence’ is used in many different ways across the definitions in the studies, included in Additional Table 6 in the Supplementary material, our understanding is based on a definition that views competences as integrative abilities. These combine knowledge, skills, attitudes, and contextual awareness, and enable their application in practice (52). According to the Council Recommendation on Key Competences for Lifelong Learning, competences are defined as ‘a combination of knowledge, skills and attitudes where: (a) knowledge comprises facts, figures, concepts, ideas, and theories that support understanding of a certain area or subject; (b) skills are the ability and capacity to carry out processes and apply existing knowledge to achieve results; and (c) attitudes describe dispositions and mindsets to act or react to ideas, persons or situations’ (52). These elements can be found either individually or collectively in all the definitions in the included studies (Additional Table 6 in the Supplementary material).

‘Attributes’ make competences measurable and observable, providing a more nuanced concept of the evolutionary process of becoming literate about environmental health issues (15). The attributes are based on well-established learning theories. In particular, they are aligned with Bloom’s Taxonomy (53), which classifies competences according to their complexity. They comply with current educational standards, such as the WHO Global Competency Framework, which explicitly require the use of operators to ensure that competences and learning objectives are clear, measurable and comparable (9). Additional Figure 1 in the Supplementary material shows the clustering of the identified attributes of the framework in combination with the four competence dimensions (access, understand, appraise, and apply).

Based on Jagals and Ebi, the development of PROF-PHL progresses from cognitive to practical competence, enabling health professionals to act (44). A health professional’s ability to act results in ‘outcomes’ (44) that can impact various levels of professional practice (27, 32). Therefore, a health professional’s actions have a direct impact on themselves and their outcomes (27, 44). Furthermore, their actions influence the healthcare system and all those involved in it (35, 44). The actions of health professionals also advocate for broader policies that protect health (27, 30). We have therefore divided the ‘professional actions and outcomes’ section of our framework into three sub-sections: ‘individual level’, ‘organizational level’, and ‘policy level’.

The proposed evidence-informed framework aims to illustrate how dimensions of competence, as well as individual and contextual factors, influence health professionals’ ability to act at the individual, organizational, and policy levels in relation to PROF-PHL development. As illustrated by the double arrows, each determinant is closely intertwined with the others. Changes to one determinant trigger chain reactions at several levels simultaneously. This nonlinear complexity, whereby nature and human action continuously influence each other through feedback loops, forms the core of planetary health (4), and our PROF-PHL framework.

To understand the connections and distinctions between the PROF-PHL dimensions, and to ensure conceptual clarity, Additional Table 10 in the Supplementary material classifies the dimensions ‘Antecedents’, ‘PROF-PHL knowledge’ ‘PROF-PHL core competences’, ‘Professional actions and outcomes’ within the PROF-PHL framework. The dimensions are categorized according to their respective roles and levels of abstraction.

Although all studies were incorporated into the development of the PROF-PHL framework, not every identified determinant was included separately in the Figure 2 to maintain clarity and transparency regarding the core constructs. Comprehensive and recurring terms were used, such as the competence dimensions (access, understand, appraise, and apply) and the levels of outcomes (individual, organizational, policy), as well as antecedents cited as examples, in an attempt to account for contextual diversity and openness. Therefore, the framework should not be understood as a definitive, rigid structure, but rather as a fundamental, context-sensitive framework that must be adapted and expanded to suit different professional practices, cultural contexts, and institutional frameworks.

### Implications of an integrative PROF-PHL framework

Following the scoping review, which provides an evidence base, our new evidence-informed PROF-PHL framework proposes a conceptual synthesis of environment-related health literacy and competence concepts under the umbrella of planetary health literacy. It integrates a proposed structure of core competences for health professionals—including accessing, understanding, appraising, and applying planetary health knowledge—with a logical model identifying individual and contextual influencing factors, as well as potential outcomes at professional, organizational, and policy levels. This structure provides a conceptual framework that could support the development of learning objectives, teaching modules, and assessment tools in future research, particularly in health professionals’ education.

It also reflects the interdisciplinary, systemic, and transformative dimensions of planetary health, emphasizing not only cognitive competences but also behavioral implementation, and systemic responsibility—principles consistent with lifelong learning (13).

The framework suggests that health professionals could promote PROF-PHL among patients and communities by collaborating across individual, organizational, and policy levels (27). The framework could inform the design of interdisciplinary curricula, training programs (29, 54), and interprofessional education initiatives (55), particularly with regard to fostering joint competences for planetary health action.

Furthermore, the framework may facilitate policy dialogue by highlighting the interplay between individual, organizational, and policy factors—including enabling and constraining conditions (34, 64). It could help decision-makers align institutional conditions with the goals of climate-resilient and sustainable health systems (2, 9).

However, it is important to clarify that these applications have not yet been empirically demonstrated. The scoping review establishes the existence of 17 heterogeneous concepts and their key determinants. The PROF-PHL framework hypothesizes a coherent, integrative structure based on these findings. The future validation of the framework must demonstrate its utility in real-world educational, clinical, and policy contexts—including its effectiveness in curriculum development, assessment, interprofessional collaboration, and policy implementation.

## Limitations

Despite its strengths, several limitations of the evidence base can also be acknowledged. The scoping review itself has several methodological limitations. As it is conceptual in nature, the evidence-informed PROF-PHL framework cannot be regarded as a direct application of scientific findings. Instead, it is a synthesis and adaptation of concepts drawn from various contexts. To validate it empirically, the determinants must be operationalized and measurement instruments developed and applied in practice, so that their relevance to health professionals can be assessed. The broad inclusion of studies applicable to health professionals shows that professional competences are not relevant to just one target group. However, including such a wide range of target groups limits the validity of the findings. They must first be empirically validated to be considered robust in terms of transferring concepts from non-professional to professional contexts. Restricting the selection of databases to MEDLINE, CINAHL, and Web of Science, supplemented by Google Scholar, and selected publishers and institutional platforms, may compromise the representativeness of the analysis. This is because these databases primarily reflect research from the Global North and from medical and health-related disciplines. This could result in the exclusion of interdisciplinary and global perspectives, particularly from the fields of environmental sciences, social sciences, and development studies. Limiting the search to English- and German-language publications represents a limitation as it compromises the representativeness of the results and may underrepresent perspectives from the Global South. This is particularly relevant in the context of planetary health, where environmental health risks and solutions depend heavily on regional, cultural, and ecological contexts. This synthesis therefore predominantly reflects the perspectives of the Global North, and should not be regarded as representative of global practice or competence development. Including heterogeneous conceptual and empirical study designs (e.g., mixed-methods studies, conceptual analyses, and frameworks, and qualitative studies) limits the findings, as it compromises the comparability, consistency, and methodological quality of the synthesis. Different approaches cannot be assessed uniformly, which restricts the potential for an evidence synthesis. The potential for subjectivity arising from the deductive-inductive coding process and the construction of the framework is a limitation. However, this was mitigated through the systematic approach outlined by (61), as well as through ongoing discussion within the team of authors, to ensure consistency and methodological transparency. No formal critical appraisal of study quality or risk of bias was conducted, as this is not a standard requirement for scoping reviews, especially when the main objective is to cluster the scope and nature of existing concepts rather than to evaluate the methodological rigor of individual studies (56). However, this approach means that the synthesis includes studies of varying methodological strength, ranging from conceptual frameworks and theoretical papers to qualitative, quantitative and mixed-methods research, which may differ significantly in their evidentiary value. Therefore, inclusion in the synthesis should not be interpreted as providing equivalent empirical support for all components of the PROF-PHL framework. The potential for overlap between the authors and the frameworks they developed is acknowledged as a limitation, given that three of the foundational publications on planetary health literacy and climate-specific health literacy included in the review were authored by members of the current research team. While this may introduce a risk of bias in the interpretation or prioritization of certain concepts, the authors emphasize that the primary goal of this work is to strengthen the visibility, coherence, and critical discourse of the emerging field of planetary health literacy, rather than to advance a personal framework. The development of the PROF-PHL framework is presented as an open, evidence-informed synthesis, not a definitive endpoint. The authors are open to refinement and critique, and welcome the framework being validated, adapted, and expanded by the broader research community to ensure its relevance, inclusivity and scientific rigor. These limitations highlight the conceptual and exploratory nature of the PROF-PHL framework, which is presented as a first step toward a professional competence framework requiring future empirical validation, operationalization, and critical evaluation.

While the scoping review provides the evidence base for the development of the PROF-PHL framework, the framework itself represents a conceptual synthesis. and extends existing ideas. It should not be understood as an empirical finding, but as an evidence-informed, integrative proposal.

## Future directions and research needs

While the development of the PROF-PHL framework is a significant step towards establishing a professional competence framework for planetary health literacy, the limitations identified in this review highlight the necessity for rigorous, context-sensitive research to enhance its validity and applicability. Due to the limited empirical evidence regarding the impact of environment-related health literacy on behaviors and environmental outcomes (57), future research must prioritize the empirical validation of the framework’s components. This should involve developing and testing operational indicators through participatory research with stakeholders from the fields of public health, education, environmental science, and healthcare systems. It should also involve taking a multilingual, inclusive, and context-sensitive approach to address global inequality in knowledge production and distribution. To ensure methodological robustness and scientific credibility, future studies should distinguish explicitly between concepts developed for the general public and those intended for professional use (19). This is because the transfer of competences from non-professional to professional contexts requires empirical validation. The heterogeneity in terminology and conceptual approaches (60) also necessitates the development of a common language and standardized measurement tools to enable comparability and integration across studies (18). Future validation efforts should test the framework across diverse professional groups, educational settings, healthcare systems, and cultural contexts, including rural and underserved communities (48). This will help to assess its adaptability and relevance beyond the contexts represented in the current review.

## Conclusion

Combining evidence synthesis and conceptual development, this scoping review demonstrates that research on health literacy concepts linking health and environment, and their application by health professionals, is an emerging and still evolving field shaped by interdisciplinary perspectives. The analysis of similarities and differences among existing environment-related health literacy and competence concepts, and their determinants reveals an evolution from unidirectional, environment-to-health concepts towards systemic, bidirectional frameworks that recognize the interdependence of human and planetary health. Recent concepts such as planetary and One Health Literacy are advancing a transdisciplinary understanding that integrates professional, institutional, and ecological dimensions. Despite significant conceptual overlap and terminological heterogeneity, the findings emphasize the need for profession-specific frameworks, that bridge the gap between health literacy at an individual level and the systemic, transformative competences required of health professionals when addressing planetary health challenges. This can help provide clearer context for the landscape research, improve understanding of and engagement with developments, and develop and refine shared understanding in discourse on existing concepts. Based on the included literature, the evidence-informed, developed conceptual framework of PROF-PHL for health professionals synthesizes and adapts key determinants from environment-related health literacy concepts, and centers the role of health professionals within broader ecological and social systems. It recognizes health-related actions must extend beyond individual behavior to include systemic change. This approach can be used to advance competences for transformative change and sustainability. Educationally, the framework offers a strategic tool for integrating planetary health thinking into the education and training of health professionals.
