# Supplementary material for: A holistic understanding of literacy approaches at the environment-health-nexus: a scoping review and framework for health professionals
Source: Front Public Health. 2026 Oct 19;14:1960284. doi: 10.3389/fpubh.2026.1960284 (PMC13724520; doi:10.3389/fpubh.2026.1960284)
Supplement: Supplementary file 1 [file Data_Sheet_1.docx]

**A holistic understanding of literacy approaches at the environment-health-nexus: a scoping review and framework for health professionals**

**Authors**

Jennifer Kallenbach M. Ed.^1,2*^, Lydia Reismann^2^, Ines Pfeffer^1,3^, Carmen Jochem^2^

1.Faculty of Human Sciences, Department of Education, MSH Medical School Hamburg, University of Applied Sciences and Medical University, Hamburg, Germany.

2. Chair of Planetary & Public Health, University of Bayreuth, Bayreuth, Germany.

3. Institute of Cognitive and Affective Neuroscience (ICAN), MSH Medical School Hamburg, University of Applied Sciences and Medical University, Hamburg, Germany.

*Correspondence:

Jennifer Kallenbach M. Ed.

jennifer.kallenbach@medicalschool-hamburg.de

**Supplementary Material**

**Table of Contents**

[**Additional Table 1: PRISMA-ScR checklist**	3](#_Toc241817644)

[**Additional Table 2: Search strings**	4](#_Toc241817645)

[**Additional Table 3: Inclusion and exclusion criteria**	4](#_Toc241817646)

[**Additional Table 4: List of excluded studies**	6](#_Toc241817647)

[**Additional Table 5: Overview of the included studies and their main characteristics**	16](#_Toc241817648)

[**Additional Table 6: Definition and determinants of included literacy and competence concepts**	20](#_Toc241817649)

[**Additional Table 7: Cluster of determinants of the included competence and literacy concepts**	26](#_Toc241817650)

[**Additional Table 8: Target groups applicability to health professionals**	28](#_Toc241817651)

[**Additional Table 9: Evidence-to-framework matrix of the PROF-PHL framework**	30](#_Toc241817652)

[**Additional Table 10: Distinct roles and levels of abstraction of the PROF-PH determinants.**	35](#_Toc241817653)

[**Additional Figure 1: Attributes of the PROF-PHL core competence dimensions**	36](#_Toc241817654)

[REFERENCES	36](#_Toc241817655)

# **Additional Table 1:** **PRISMA-ScR checklist**

| **SECTION** | **ITEM** | **PRISMA-ScR CHECKLIST ITEM** | **REPORTED IN SECTION** |
| --- | --- | --- | --- |
| **TITLE** | | | |
| Title | 1 | Identify the report as a scoping review. | Titel |
| **ABSTRACT** | | | |
| Structured summary | 2 | Provide a structured summary that includes (as applicable): background, objectives, eligibility criteria, sources of evidence, charting methods, results, and conclusions that relate to the review questions and objectives. | Abstract |
| **INTRODUCTION** | | | |
| Rationale | 3 | Describe the rationale for the review in the context of what is already known. Explain why the review questions/objectives lend themselves to a scoping review approach. | Background |
| Objectives | 4 | Provide an explicit statement of the questions and objectives being addressed with reference to their key elements (e.g., population or participants, concepts, and context) or other relevant key elements used to conceptualize the review questions and/or objectives. | Background |
| **METHODS** | | | |
| Protocol and registration | 5 | Indicate whether a review protocol exists; state if and where it can be accessed (e.g., a Web address); and if available, provide registration information, including the registration number. | Method |
| Eligibility criteria | 6 | Specify characteristics of the sources of evidence used as eligibility criteria (e.g., years considered, language, and publication status), and provide a rationale. | Method, Additional Table 3 |
| Information sources* | 7 | Describe all information sources in the search (e.g., databases with dates of coverage and contact with authors to identify additional sources), as well as the date the most recent search was executed. | Method |
| Search | 8 | Present the full electronic search strategy for at least 1 database, including any limits used, such that it could be repeated. | Additional Table 2 |
| Selection of sources of evidence† | 9 | State the process for selecting sources of evidence (i.e., screening and eligibility) included in the scoping review. | Method, Additional Table 4 |
| Data charting process‡ | 10 | Describe the methods of charting data from the included sources of evidence (e.g., calibrated forms or forms that have been tested by the team before their use, and whether data charting was done independently or in duplicate) and any processes for obtaining and confirming data from investigators. | Method |
| Data items | 11 | List and define all variables for which data were sought and any assumptions and simplifications made. | Method |
| Critical appraisal of individual sources of evidence§ | 12 | If done, provide a rationale for conducting a critical appraisal of included sources of evidence; describe the methods used and how this information was used in any data synthesis (if appropriate). | n/a |
| Synthesis of results | 13 | Describe the methods of handling and summarizing the data that were charted. | Method |
| **RESULTS** | | | |
| Selection of sources of evidence | 14 | Give numbers of sources of evidence screened, assessed for eligibility, and included in the review, with reasons for exclusions at each stage, ideally using a flow diagram. | Results, Figure 1 |
| Characteristics of sources of evidence | 15 | For each source of evidence, present characteristics for which data were charted and provide the citations. | Table 1, Additional Table 5 & 6 |
| Critical appraisal within sources of evidence | 16 | If done, present data on critical appraisal of included sources of evidence (see item 12). | n/a |
| Results of individual sources of evidence | 17 | For each included source of evidence, present the relevant data that were charted that relate to the review questions and objectives. | Results |
| Synthesis of results | 18 | Summarize and/or present the charting results as they relate to the review questions and objectives. | Results, Discussion |
| **DISCUSSION** | | | |
| Summary of evidence | 19 | Summarize the main results (including an overview of concepts, themes, and types of evidence available), link to the review questions and objectives, and consider the relevance to key groups. | Discussion, Figure 2, table 1, Additional Table 7, 8, 9 & 10 |
| Limitations | 20 | Discuss the limitations of the scoping review process. | Limitations |
| Conclusions | 21 | Provide a general interpretation of the results with respect to the review questions and objectives, as well as potential implications and/or next steps. | Conclusion |
| **FUNDING** | | | |
| Funding | 22 | Describe sources of funding for the included sources of evidence, as well as sources of funding for the scoping review. Describe the role of the funders of the scoping review. | Declarations |

Preferred Reporting Items for Systematic reviews and Meta-Analyses extension for Scoping Reviews (PRISMA-ScR) Checklist according to the Joanna Briggs Institute (JBI) (Tricco et al., 2018)

# **Additional Table 2: Search strings**

**Search string MEDLINE and CINAHL via EBSCO**

Date of search: 31. January 2025

(MH “Health Personnel” OR (Health* N4 (personnel* OR profession* OR worker* OR practitioner* OR assistant* OR provider* OR community* OR manager*)) OR (Care N1 (provider* or giver*)) OR ((Medic* or clinic*) N1 (professional* or personnel* or practitioner* or assistant* or expert* or staff* or technician*)) OR Nurs* OR Doctor* OR Physiotherapist* OR Psychologist* OR (Ambulance N4 worker*) OR Midwif* OR Dietician* OR Nutritionist* OR Dentist* OR Physician*) AND (MH "Health Literacy" OR TI literacy* OR AB literacy* OR TI competenc* OR AB competenc*) AND (model OR theory OR framework OR approach OR concept OR strategy OR defin* OR understanding OR characteris* OR identif* OR proposal) (MH "Environmental Health" OR MH "Environmental Sustainability" OR MH "Conservation of Natural Resources+" OR MH "Carbon Footprint" OR MH "Climate Change" OR TI ("Education for Sustainable Development" OR eco#health* OR (eco* N1 health*) OR one#health OR (one N1 health) OR ((medic* OR health*) N3 (planet* OR biodiversity* OR ecological)) OR (global* N1 warm*) OR (climate* N3 (act* OR chang* OR resilien* OR protect* OR mitigat* OR adaptation* OR crisis OR distress*)) OR climate#sensitive OR pro#environmental OR (sustainab* N3 (resilien* OR environmental OR development* OR ecolog* OR transform* OR educat*))) OR AB ("Education for Sustainable Development" OR eco#health* OR (eco* N1 health*) OR one#health OR (one N1 health*) OR ((medic* OR health*) N3 (planet* OR biodiversity* OR ecological)) OR (global* N1 warm*) OR (climate* N3 (act* OR chang* OR resilien* OR protect* OR mitigat* OR adaptation* OR crisis OR distress*)) OR climate#sensitive OR pro#environmental OR (sustainab* N3 (resilien* OR environmental OR development* OR ecolog* OR transform* OR educat*))))

Total results CINAHL: 514; total results MEDLINE: 406

**Search string Web of Science**

Date of search: 31. January 2025

TS=(("Health Personnel" OR (health* NEAR/4 (personnel* OR profession* OR worker* OR practitioner* OR assistant* OR provider* OR community* OR manager*)) OR (care NEAR/1 (provider* OR giver*)) OR ((medic* OR clinic*) NEAR/1 (professional* OR personnel* OR practitioner* OR assistant* OR expert* OR staff* OR technician*)) OR nurs* OR doctor* OR physiotherapist* OR psychologist* OR (ambulance NEAR/4 worker*) OR midwif* OR dietician* OR nutritionist* OR dentist* OR physician*) AND (("Health Literacy" OR literacy* OR competenc*) NEAR/9 (model OR theory OR framework OR approach OR concept OR strategy OR defin* OR understanding OR characteris* OR identif* OR propos*)) AND ("Education for Sustainable Development" OR "Environmental Health" OR "Environmental Sustainability" OR "Conservation of Natural Resources" OR "Carbon Footprint" OR "Climate Change" OR eco$ health* OR (eco* NEAR/1 health*) OR one$health OR (one NEAR/1 health) OR ((medic* OR health*) NEAR/3 (planet* OR biodiversity* OR ecological)) OR (global* NEAR/1 warm*) OR (climate* NEAR/3 (act* OR chang* OR resilien* OR protect* OR mitigat* OR adaptation* OR crisis OR distress*)) OR climate*sensitive OR pro*environmental OR (sustainab* NEAR/3 (resilien* OR environmental OR development* OR ecolog* OR transform* OR educat*))))

Total results: 679

**Key words Google Scholar**

Date of search: 31. January 2025

("Health Literacy" OR literacy* OR competence*) AND (“Health Personnel” OR “Health personnel*” OR “Health profession*” OR “Health worker*” OR “Health practitioner*” OR “Health assistant*” OR “Health provider*” OR “Health community*” OR “Health manager*” OR “Care provider*” OR “Care giver*” OR “Medic*professional*” OR “Medic*personnel*” OR “Medic* practitioner*” OR “Medic* assistant*” OR “Medic*expert*” OR “Medic* staff*” OR “Medic* technician*” “clinic* professional*” OR “Clinic* personnel*” OR “Clinic* practitioner*” OR “clinic* assistant*” OR “clinic* expert*” OR “clinic* staff*” OR “clinic* technician*” OR Nurs* OR Doctor* OR Physiotherapist* OR Psychologist* OR “Ambulance worker*” OR Midwif* OR Dietician* OR Nutritionist* OR Dentist* OR Physician*) AND ("Environmental Health" OR "Environmental Sustainability" OR "Conservation of Natural Resources " OR "Carbon Footprint" OR"Climate Change" OR "Education for Sustainable Development" OR “eco health* OR “one health OR “medic*planet*” OR “medic* biodiversity*” OR “medic* ecological” OR “health* planet*” OR “health* biodiversity*” OR “health* ecological” OR “global* warm*” OR “climate*act*” OR “climate* chang*” OR “climate* resilien*” OR “climate* protect*” OR “climate* mitigat*” OR “climate* adaptation*” OR “climate* crisis” OR “climate* distress*” OR “climate sensitive” OR “pro environmental” OR “sustainab* resilien*” OR “sustainab* environmental” OR “sustainab* development*” OR ecolog* OR transform* OR educat*) AND (model OR theory OR framework OR approach OR concept OR strategy OR defin* OR understanding OR characteris* OR identif* OR proposal)

Total results: 15

# **Additional Table 3: Inclusion and exclusion criteria**

|  | **Inclusion criteria** | **Explanation** | **Exclusion citeria** | **Explanation** |
| --- | --- | --- | --- | --- |
| **POPULATION** | ‘Applicable’ to health professionals according to the WHO ISCO classification (2019) who have completed their training or are in training or studying | ‘Applicable’ means:  a) Content-related relevance: Whether the core competences are directly transferable to the role of health professionals.  b) Contextual alignment: Whether application scenarios (e.g. patient counselling, health promotion, political advocacy) are relevant to health professional practice.  c) Incorporation of the target group: Although these concepts were originally developed for other groups, they do not explicitly exclude professional competences. Instead, they provide a foundation for further development in professional contexts. This is evident from the fact that many of the included studies address both healthcare professionals and other target groups (e.g. Jochem et al., 2022; Finn and O'Fallon, 2017; Grabow et al., 2023). This suggests that competences are not significant within a single professional group alone, but only acquire significance through interaction with other stakeholders. | Studies that do not demonstrate any explicit or implicit relevance to the role of health professionals in clinical, educational, or public health practice. | Including studies that are not explicitly or implicitly relevant to the role of health professionals in clinical, educational or public health practice would compromise the professional specificity, practical applicability and scientific credibility of the findings. This criterion is essential for ensuring that the resulting findings are recognised as a professional literacy framework rather than as general environmental-related health literacy. |
| **CONCEPT** | Articles considering concepts, models, theories, definitions, or characterizations of health literacies or competences. | This involves the systematic identification of articles examining the conceptual aspects of health literacy and/or competence development, as well as their determinants. | Articles focusing solely on literacy or competence without health aspects OR Articles mentioning health literacies or competences without providing concepts, models, definitions or theoretical background. | Articles that merely refer to health literacy or competence but do not provide any information on the understanding of these concepts do not provide a basis for systematically understanding concepts of health literacy or competence. |
| **CONTEXT** | Literacy or competence concepts and models integrating planetary, climate, and environmental issues into health literacy development OR Articles considering literacy or competence concepts and models or related to environment, ecosystems, climate, and/or biodiversity in relation to health and sustainability. | As the review focuses on identifying health literacy and competence concepts relating to the environment-health nexus, it includes all articles that combine an understanding of health literacy or competence with environmental and health-related aspects. | Articles solely addressing health literacy or competence without planetary, climate, or environmental aspects. | This concerns the examination of the link between the environment and health within the context of health literacy and competence frameworks, studies with no environmental relevance whatsoever are excluded |
| **TIME** | Published from inception to January 31, 2025 | In a systematic review, the aim is to include all available, relevant literature up to the time of data extraction to ensure the search process is complete, transparent and reproducible. This is a key requirement of the PRISMA-ScR guidelines (Page et al., 2021). | Publications from February 1, 2025, onwards | The research process has already been completed here. |
| **TYPE OF SOURCES** | Primary research  Secondary research  Thesis and Dissertations  Grey literature  Commentaries | In order to facilitate a comprehensive, transdisciplinary and context-relevant synthesis of the existing literature, the literature review will incorporate primary and secondary research, doctoral theses, grey literature and commentaries. This approach is informed by the following scientific and methodological considerations: Firstly, the inclusion of primary research is standard practice in scoping reviews, particularly for identifying concepts and their practical application (Peters et al., 2024). Secondary research is also explicitly included in scoping reviews to track the development and evolution of research fields (Peters et al., 2024). Dissertations are also routinely included in scoping reviews to address the 'grey literature' gap (Peters et al., 2024). Grey literature is explicitly recommended for inclusion in the PRISMA-ScR and JBI guidelines to enhance the representativeness and practical relevance of the analysis (Page et al., 2021; Peters et al., 2024). Commentaries are often included in scoping reviews to track the development of discourses and paradigm shifts (Peters et al., 2024). | Media  Blogs  Editorials | Articles from the media, blogs and editorials were not included as they do not meet the methodological requirements for being considered a scientific, verifiable and validated source (Peters et al., 2024). |
| **LANGUAGE** | Full text available in English or German | Most international research on topics such as planetary health, environmental health and health literacy is published in English because it is the primary language of communication within the scientific community.  German, on the other hand, is an important language in European research, particularly in Germany, Austria and Switzerland. As these countries have comparable healthcare profession education systems, findings are more readily transferable between them (Gani et al., 2023).  The PRISMA-ScR guidelines (Page et al., 2021) and the JBI methodology (Peters et al., 2024) allow for restriction to specific languages, provided this is transparently documented. The reasons outlined above ensure this. | All other languages | Furthermore, translating full-text articles into multiple languages is time-consuming and costly. It requires qualified translators and carries a high risk of distortion and loss of nuance. As insufficient resources are available for multilingual, high-quality analysis, particularly without external support or funding, the focus has been placed on studies in English and German. |

* ISCO: International Standard Classification of Occupational Health (World Health Organisation, 2019)

# **Additional Table 4: List of excluded studies**

| **List of excluded studies** | **References (n = 196)** | |  |
| --- | --- | --- | --- |
| **Name/s of the author/s** | **Year** | **Title** | **Reason of exclusion** |
| Adeyemi, O. A.; Agbabiaka, T. O.; Sujon, H. | 2024 | Global One Health post-graduate programs: a review | no literacy or competence concept |
| Álvarez-Nieto, Carmen; López-Medina, Isabel M.; Abad, Manuel Linares; Grande-Gascón, M. Luisa; Álvarez-García, Cristina | 2017 | Curriculum nurse and strategies training on environmental sustainability and climate change | no literacy or competence concept |
| Amin, Shaimaa Mohamed; El-Monshed, Ahmed Hashem; Khedr, Mahmoud Abdelwahab; Morsy, Omima Mohamed Ibrahim; El-Ashry, Ayman Mohamed | 2024 | Future Nurses in a Changing Climate: Exploring the Relationship Between Environmental Literacy and Climate Anxiety | no health aspects linking literacy or competence |
| Amuguni, H. J.; Mazan, M.; Kibuuka, R. | 2017 | Producing Interdisciplinary Competent Professionals: Integrating One Health Core Competencies into the Veterinary Curriculum at the University of Rwanda | no literacy or competence concept |
| Amuguni, Hellen; Bikaako, Winnie; Naigaga, Irene; Bazeyo, William | 2019 | Building a framework for the design and implementation of One Health curricula in East and Central Africa: OHCEAs One Health Training Modules Development Process | no literacy or competence concept |
| American Association of Occupational Health Nurses | 2007 | Competencies in occupational and environmental health nursing | no literacy or competence concept |
| Asaduzzaman, Muhammad; Ara, Rifat; Afrin, Sadia; Meiring, James E.; Saif-Ur-Rahman, K. M. | 2022 | Planetary Health Education and Capacity Building for Healthcare Professionals in a Global Context: Current Opportunities, Gaps and Future Directions | no literacy or competence concept |
| Astle, Barbara | 2021 | Planetary Health in Nursing: A Transdisciplinary Equity-Centered Approach | no literacy or competence concept |
| Azevedo, José; Marques, Margarida | 2017 | Climate literacy: a systematic review and model integration | no health aspects linking literacy or competence |
| Baena-Morales, Salvador; Fröberg, Andreas | 2023 | Towards a more sustainable future: simple recommendations to integrate planetary health into education | no literacy or competence concept |
| Baines, Anna E.; Errecaborde, Kaylee; Ames, Trevor R.; Goldsmith, Timothy J.; Kinzer, Hannah; Mahero, Michael; Molitor, Thomas; Molgaard, Laura; Pelican, Katharine; Ponder, Julia B.; Travis, Dominic A.; Willette, Michelle; Wolf, Tiffany M.; Zou, Xunwen; Wells, Scott J. | 2024 | One Health as a Core Component of Veterinary Medicine: Defining Day-1 Public Health Competencies for the Veterinary Workforce | no literacy or competence concept |
| Behera, Manas R.; Behera, Deepanjali; Satpathy, Sudhir K. | 2020 | Planetary health and the role of community health workers | no literacy or competence concept |
| Bell, Erica; Horton, Graeme; Blashki, Grant; Seidel, Bastian M. | 2012 | Climate change: could it help develop ’adaptive expertise’? | no literacy or competence concept |
| Bellack, J. P.; Musham, C.; Hainer, A.; Graber, D. R.; Holmes, D. | 1996 | Environmental health competencies: a survey of U.S. nurse practitioner programs | no literacy or competence concept |
| Bevan, James; Blyth, Rachel; Russell, Benjamin; Holtgrewe, Lydia; Cheung, Annie Hoi Ching; Austin, Isobel; Shah, Viraj; Butler, Megan; Fraser, Simon | 2023 | Planetary health and sustainability teaching in UK medical education: A review of medical school curricula | no literacy or competence concept |
| Bickler, G.; Morton, S.; Menne, B. | 2020 | Health and sustainable development: an analysis of 20 European voluntary national reviews | wrong population |
| Boakye, Dorothy Serwaa; Kwashie, Atswei Adzo; Adjorlolo, Samuel; Korsah, Kwadwo Ameyaw | 2024 | Nursing Education for Sustainable Development: A Concept Analysis | no literacy or competence concept |
| Bonnamy, James; Levett-Jones, Tracy; Carr, Bethany; Lokmic-Tomkins, Zerina; Maguire, Jane; Catling, Christine; Pich, Jacqueline; Fields, Lorraine; Brand, Gabrielle | 2024 | Australian and New Zealand nursing and midwifery educators’ planetary health knowledge, views, confidence and teaching practices: A descriptive survey study | no literacy or competence concept |
| Boucher, Beatrice A.; Manafo, Elizabeth; Boddy, Meaghan R.; Roblin, Lynn; Truscott, Rebecca | 2017 | The Ontario Food and Nutrition Strategy: identifying indicators of food access and food literacy for early monitoring of the food environment | no literacy or competence concept |
| Boudrias, Michel | 2010 | CCEP-I: Climate Science Meets Social Psychology and Strategic Communications: Applying proven learning and communications strategies to climate literacy in the San Diego Region | wrong population, no literacy or competence concept |
| Brennan, Meagan E.; Madden, Diana L. | 2023 | The evolving call to action for including climate change and environmental sustainability themes in health professional education: A scoping review | no literacy or competence concept |
| Brewer, Dawn; Bellamy, Hannah; Hoover, Anna; Koempel, Annie; Gaetke, Lisa | 2019 | Nutrition and Environmental Pollution Extension Curriculum Improved Diet-Related Behaviors and Environmental Health Literacy | wrong population |
| Brown, Maura J.; Forster, Bruce B.; McInnes, Matthew D. F.; Komar, Madeline A.; Amin, Parthiv; Atwal, Sukhreet; Chen, Shihan; Hamwi, Milad; Ladak, Rahman; Malik, Aleena; McKee, Hayley; Wang, Mark; Yang, Joseph; Hamel, Candyce; Hanneman, Kate | 2024 | Canadian Association of Radiologists Statement on Planetary Health Education in Radiology | no literacy or competence concept |
| Buka, I.; Brennan, L.; Tarrabain, J.; Aghazadeh, S.; Brune, Drisse, MN | 2020 | Need for global core competencies in Child Health and the Environment: a Canadian perspective | no literacy or competence concept |
| Cadet, Myriam Jean | 2022 | Integrating Climate Change Concepts into Advanced Practice Registered Nurses Curricula with the Application of the National Organization of Nurse Practitioner Faculties Competencies | no literacy or competence concept |
| Capetola, Teresa; Noy, Sue; Patrick, Rebecca | 2022 | Planetary health pedagogy: Preparing health promoters for 21st-century environmental challenges | no literacy or competence concept |
| Carducci, A.; Fiore, M.; Lorini, C.; Federigi, I.; Verani, M.; Ferrante, M.; Bonaccorsi, G. | 2022 | Environmental Health Literacy: an index to study its relations with pro-environmental behaviors | wrong population |
| Cebrián, Gisela; Junyent, Mercè | 2015 | Competencies in Education for Sustainable Development: Exploring the Student Teachers’ Views | wrong population, no literacy or competence concept |
| Cengiz, Burcu; Bahar, Zühal | 2023 | The Characteristics of University Students on Environmental Attitude and Environmental Literacy Level: Example of Faculty of Nursing | no health aspects linking literacy or competence |
| Cesar, Flaviane Cristina Rocha; Sousa, Thassara Felipe de; Alves, Angela Gilda; Moraes, Katarinne Lima; Barbosa, Maria Alves; Oliveira, Lizete Malagoni de Almeida Cavalcante | 2022 | Competencies of health personnel for the practice of health literacy in Brazil: A Delphi consensus survey | no environmental focus |
| Christie, Gillian P.; Ratzan, Scott C.; Logan, R. A.; Siegel, E. R. | 2020 | Beyond the Bench and Bedside: Health Literacy Is Fundamental to Sustainable Health and Development - HEALTH LITERACY IN CLINICAL PRACTICE AND PUBLIC HEALTH: NEW INITIATIVES AND LESSONS LEARNED AT THE INTERSECTION WITH OTHER DISCIPLINES | no environmental focus |
| Colak, M.; Dogan, R.; Dogan, S. | 2025 | Effect of Climate Change and Health Course on Global Warming Knowledge and Attitudes, Environmental Literacy, and Eco-Anxiety Level of Nursing Students: A Quasi-Experimental Study | no health aspects linking literacy or competence |
| Coleman, Clifford | 2011 | Teaching health care professionals about health literacy: a review of the literature | no environmental focus |
| Coleman, Clifford A.; Fromer, Amber | 2015 | A health literacy training intervention for physicians and other health professionals | no environmental focus |
| Coleman, Clifford A.; Hudson, Stan; Maine, Lucinda L. | 2013 | Health literacy practices and educational competencies for health professionals: a consensus study | no environmental focus |
| Czerwinski, Megan A. | 2020 | A Dissertation on Sustainability Competence: Directions for Nursing | no literacy or competence concept |
| Da Jeong, Woon; Kim, Gwang Suk; Park, Min Kyung | 2022 | Validity and Reliability of the Korean Version of the Climate, Health, and Nursing Tool | no literacy or competence concept |
| Dagal, Melike; Firat Kilic, Hülya | 2023 | Environmental Literacy Levels of Nursing Students: A Descriptive Study | no health aspects linking literacy or competence |
| Daniš, Petr | 2013 | A new definition of environmental literacy and a proposal for its international assessment in PISA 2015 | no health aspects linking literacy or competence |
| Delamare, Estelle; Greipl, Cora; Peterschmitt, Léo; Ruiz De Castañeda, Rafael; Sommer, Johanna; Jackson, Yves; Flahault, Antoine | 2022 | Enseigner la santé planétaire dans le curriculum médical prégradué en Suisse | foreign language |
| Demorest, S.; Cook, C.; Schenk, E.; Harris, L. W.; Earley, A. | 2024 | School of Nursing Climate Commitment: Nursing Faculty Bring Climate to the Classroom | no health aspects linking literacy or competence |
| Desmond, Sharon | 2016 | Implementing climate change mitigation in health services: the importance of context | no health aspects linking literacy or competence |
| El-Sayed, Ahmed Abdelwahab Ibrahim; Alsenany, Samira Ahmed; Abdelaliem, Sally Mohammed Farghaly; Asal, Maha Gamal Ramadan | 2024 | Exploring Organisational Agility’s Impact on Nurses’ Green Work Behaviour: The Mediating Role of Climate Activism | no literacy or competence concept |
| Eustachio, JHPP; Filho, W. L.; Baars, C.; Barbosa-Silva, J.; Lourencao, M.; Barbir, J.; Roseler, J.; Buddig, S.; de, Lima, PQ; Bandos, M. C. | 2024 | Fostering the discussion of planetary health in occupational therapy and physiotherapy. [Review] | no literacy or competence concept |
| Faerron Guzmán, Carlos A.; Porter, Teddie | 2021 | The Planetary Health Education Framework | no literacy or competence concept |
| Flaten, Carol B.; Pechacek, Judith M.; Potter, Teddi M.; Mueller, Christine | 2023 | Planetary health in nursing curricula: How one school transformed nursing curricula as they integrated planetary health concepts with the 2021 AACN essentials | no literacy or competence concept |
| Floss, Mayara; Vieira Ilgenfritz, Carlos Augusto; Rodrigues, Ylana Elias; Cláudia Dilda, Anna; Borngräber Corrêa, Ana Paula; Azevedo Conte de Melo, Diego; Falceto Barros, Enrique; Faerron Guzmán, Carlos Alberto; Devlin, Erin; Saldiva, Paulo Hilário Nascimento; Khoo, Su-Ming; Gonçalves, Marcelo Rodrigues | 2021 | Development and Assessment of a Brazilian Pilot Massive Open Online Course in Planetary Health Education: An Innovative Model for Primary Care Professionals and Community Training | no literacy or competence concept |
| Frankson, Rebekah; Hueston, William; Christian, Kira; Olson, Debra; Lee, Mary; Valeri, Linda; Hyatt, Raymond; Annelli, Joseph; Rubin, Carol | 2016 | One Health Core Competency Domains | no literacy or competence concept |
| Freedman, Darcy A.; Bess, Kimberly D.; Tucker, Holly A.; Boyd, David L.; Tuchman, Arleen M.; Wallston, Kenneth A. | 2009 | Public Health Literacy Defined | no environmental focus |
| Gehle, Kimberly S.; Crawford, Jewel L.; Hatcher, Michael T. | 2011 | Integrating Environmental Health into Medical Education | no literacy or competence concept |
| Goodman, Benny; East, Linda | 2014 | The ’sustainability lens’: a framework for nurse education that is ’fit for the future’ | no health aspects linking literacy or competence |
| Gray, Kathleen M. | 2018 | From Content Knowledge to Community Change: A Review of Representations of Environmental Health Literacy | no literacy or competence concept |
| Gray, Kathleen Marie; Blanchard, Margaret R.; LePrevost, Catherine E. | 2018 | Characterizing Environmental Health Literacy Related to Fish Consumption Advisories: Knowledge and Beliefs of Informal Educators in a Southeastern State | wrong population |
| Greenwald, L.; Blanchard, O.; Hayden, C.; Sheffield, P. | 2022 | Climate and health education: A critical review at one medical school. [Review] | no literacy or competence concept |
| Grimley, Benjamin L. | 2020 | A 3-D Multimodal Approach to K-8 Environmental Health Literacy | no environmental focus |
| Guzmán, Carlos A. Faerron; Aguirre, A. Alonso; Astle, Barbara; Barros, Enrique; Bayles, Brett; Chimbari, Moses; El-Abbadi, Naglaa; Evert, Jessica; Hackett, Finola; Howard, Courtney; Jennings, Jonathan; Krzyzek, Amy; LeClair, Jessica; Maric, Filip; Martin, Olwenn; Osano, Odipo; Patz, Jonathan; Potter, Teddie; Redvers, Nicole; Trienekens, Noortje; Walpole, Sarah; Wilson, Lynda; Xu, Chenchen; Zylstra, Matthew | 2021 | A framework to guide planetary health education | no literacy or competence concept |
| Hickman, Amy Christine; Johnson, Rebecca Lauren; Lawler, Sheleigh Patricia | 2022 | Health-promoting pedagogy: Using reflexivity to support learning and action in planetary health education | no literacy or competence concept |
| Ho, Ken Hok Man; Cheng, Ho Yu; McKenna, Lisa; Cheung, Daphne Sze Ki | 2024 | Nursing and midwifery in a changing world: Addressing planetary health and digital literacy through a global curriculum | no environmental focus |
| Hollweg, Karen; Taylor, Jason; Bybee, Rodger W.; Marcinkowski, Thomas J.; McBeth, William C.; Zoido, Pablo; North American Association for Environmental Education | 2015 | Developing a Framework for Assessing Environmental Literacy: Executive Summary | no health aspects linking literacy or competence |
| Huss, Norma; Ikiugu, Moses N.; Hackett, Finola; Sheffield, Perry E.; Palipane, Natasha; Groome, Jonathan | 2020 | Education for sustainable health care: From learning to professional practice | no literacy or competence concept |
| Incesu, Olga; Yas, Merve Altiner | 2024 | The relationship between nursing students’ environmental literacy and awareness of Global Climate Change | no health aspects linking literacy or competence |
| Irlam, J. H.; Reid, S.; Rother, H-A | 2023 | Education about planetary health and sustainable healthcare: A national Delphi panel assessment of its integration into health professions education in South Africa | no literacy or competence concept |
| Irlam, James H.; Scheerens, Charlotte; Mash, Bob | 2023 | Planetary health and environmental sustainability in African health professions education | no literacy or competence concept |
| Jadotte, Y. T.; Caron, R. M.; Kearney, G. D. | 2022 | Ecosystemic Theory, Practice, and Policy: Training Recommendations for Environmental Public Health | no literacy or competence concept |
| Kapan, Rukiye; Yardımcı Gürel, Tuğba | 2022 | An evaluation of the environmental literacy levels of nursing students in Turkey | no health aspects linking literacy or competence |
| Karuranga, Suvi; Sørensen, Kristine; Coleman, Clifford; Mahmud, Amina Jama | 2017 | Health Literacy Competencies for European Health Care Personnel | no environmental focus |
| Keating, Stacen A.; Berland, Alex; Capone, Kathleen; Chickering, Miriam J. | 2021 | Global Nursing Education: International Resources Meet the NLN Core Competencies for Nurse Educators | no environmental focus |
| Keating, Stacen A.; Vetter, Mary Jo; Klar, Robin Toft; Wright, Fay | 2022 | Integrating Climate Change in the Curriculum: Using Instructional Design Methods to Create an Educational Innovation for Nurse Practitioners in a Doctor of Nursing Practice Program | no literacy or competence concept |
| Kılıç, Hülya Fırat; Canatan, Sinem Dağ; Abiç, Arzu; Gök, Nur Demet | 2024 | Evaluating the Relationship between Nursing Students’ Environmental Awareness and Sensitivity and Their Environment Literacy | no health aspects linking literacy or competence |
| Kligler, B.; Pinto, Zipp, G; Rocchetti, C.; Secic, M.; Ihde, E. S. | 2021 | The impact of integrating environmental health into medical school curricula: a survey-based study | no literacy or competence concept |
| Kötter, Thomas; Hoschek, Mieke; Pohontsch, Nadine Janis; Steinhäuser, Jost | 2023 | Planetary Health in der curricularen Lehre im Fach Humanmedizin ‐ eine qualitative Studie zur Evaluation einer Lehr-/Lernintervention | no literacy or competence concept |
| Langdon, Cathy; Cammer, Allison; Dobson, Roy; Lieffers, Jessica | 2023 | Food Literacy: An Emergent Concept for Dietetic Education...Society for Nutrition Education and Behavior (SNEB), 55th Annual Conference, July 20-23, 2023, Washington, DC | fulltext not available |
| Larsen, Roxanne J. | 2021 | Shared Curricula and Competencies in One Health and Health Professions Education | no literacy or competence concept |
| Larsson, L. S.; Butterfield, P. | 2002 | Mapping the future of environmental health and nursing: strategies for integrating national competencies into nursing practice | no literacy or competence concept |
| LaVeaux, Deborah; Simonds, Vanessa W.; Picket, Velma; Cummins, Jason; Calkins, Erin | 2018 | Developing a Curriculum for Change: Water and Environmental Health Literacy in a Native American Community | wrong population |
| Leal Filho, Walter; Eustachio, João Henrique Paulino Pires; Paucar-Caceres, Alberto; Cavalcanti-Bandos, Melissa Franchini; Nunes, Cintia; Vílchez-Román, Carlos; Quispe-Prieto, Silvia; Brandli, Luciana Londero | 2022 | Planetary Health and Health Education in Brazil: Towards Better Trained Future Health Professionals | no literacy or competence concept |
| Lee, Jung Jae; Huang, Yangxi; Yan, Yongyang; Lui, Yan Wing; Ye, Fen | 2024 | Integrating climate change and sustainability in nursing education | no literacy or competence concept |
| Leffers, Jeanne; Levy, Ruth McDermott; Nicholas, Patrice K.; Sweeney, Casey F. | 2017 | Mandate for the Nursing Profession to Address Climate Change Through Nursing Education | no literacy or competence concept |
| Leonardi, Giovanni S.; Zeka, Ariana; Ashworth, Matthew; Bouland, Catherine; Crabbe, Helen; Duarte-Davidson, Raquel; Etzel, Ruth A.; Giuashvili, Nia; Gokdemir, Ozden; Hanke, Wojciech; van den Hazel, Peter; Jagals, Paul; Khan, Ejaz Ahmad; Martin-Olmedo, Piedad; Pett, Joseph; Ruadze, Ekaterine; Santamaria, Maria Grazia; Semenza, Jan C.; Sorensen, Cecilia; Vardoulakis, Sotiris; Yip, Fuyuen; Lauriola, Paolo | 2024 | Building competency to deal with environmental health challenges: experiences and a proposal | no literacy or competence concept |
| Liao, W.; Yang, L.; Zhong, S.; Hess, J. J.; Wang, Q.; Bao, J.; Huang, C. | 2019 | Preparing the next generation of health professionals to tackle climate change: Are China’s medical students ready? | no literacy or competence concept |
| Limaye, Vijay S.; Grabow, Maggie L.; Stull, Valerie J. | 2022 | Improving literacy in climate and health | full text not available |
| Lokmic-Tomkins, Z.; Barbour, L.; LeClair, J.; Luebke, J.; McGuinness, S. L.; Limaye, V. S.; Pillai, P.; Flynn, M.; Kamp, M. A.; Leder, K.; Patz, J. A. | 2024 | Integrating planetary health education into tertiary curricula: a practical toolbox for implementation | no literacy or competence concept |
| Luo, Owen Dan; Londono, Candelaria Aristizabal; Prince, Nicole; Iny, Ericka; Warnock, Tyler; Cropper, Kayla; Girgis, Sam; Walker, Celia | 2023 | The Climate Wise slides: An evaluation of planetary health lecture slides for medical education | no literacy or competence concept |
| MacKenzie-Shalders, K.; Zadow, G.; Hensley-Hackett, K.; Marko, S.; McLean, M. | 2024 | Rapid review: Guides and frameworks to inform planetary health education for health professions | no literacy or competence concept |
| Madden, Diana Lynne; McLean, Michelle; Brennan, Meagan; Moore, Aishah | 2020 | Why use indicators to measure and monitor the inclusion of climate change and environmental sustainability in health professions’ education? | no literacy or competence concept |
| Mahase, Elisabeth | 2024 | Planetary health prescriptions: Finland’s new physician role connects human and climate health | no literacy or competence concept |
| Malmqvist, Ebba; Oudin, Anna | 2024 | Bridging disciplines-key to success when implementing planetary health in medical training curricula | no literacy or competence concept |
| Mani, Z. A.; Naylor, K.; Goniewicz, K. | 2024 | Essential competencies of nurses for climate change response in Saudi Arabia: A rapid literature review | no literacy or competence concept |
| Markowitz Grossmann, Dina | 2020 | One Health Education: Connecting Humans, Animals, and the Environment | no literacy or competence concept |
| Martin-Garin, Alexander; Millan-Garcia, Jose Antonio; Leon, Inigo; Oregi, Xabat; Estevez, Julian; Marieta, Cristina | 2021 | Pedagogical Approaches for Sustainable Development in Building in Higher Education | no literacy or competence concept |
| Masarwa, Sonya Kabha | 2021 | Environmental health literacy: The Relation Between Perceived Knowledge, Attitudes and Reported Behavior Among Family Physicians and Pediatricians in Israel | full text not available |
| Massey, Philip M.; Prelip, Michael; Calimlim, Brian M.; Quiter, Elaine S.; Glik, Deborah C. | 2012 | Contextualizing an expanded definition of health literacy among adolescents in the health care setting | no environmental focus |
| Matesanz, Maria Majadas; Da Silva Caeiro, Sandra Sofia Ferreira; Bacelar Nicolau, Paula | 2023 | Anticipating Future Needs in Key Competences for Sustainability in Two Distance Learning Universities of Spain and Portugal | wrong population |
| McBride, B. B.; Brewer, C. A.; Berkowitz, A. R.; Borrie, W. T. | 2013 | Environmental literacy, ecological literacy, eco literacy: What do we mean and how did we get here? | no health aspects linking literacy or competence |
| McKinnon, Sarah; Breakey, Suellen; Fanuele, Jenny R.; Kelly, Debra E.; Eddy, Emily Zeman; Tarbet, Amanda; Nicholas, Patrice K.; Ros, Ana M. Viamonte | 2022 | Roles of health professionals in addressing health consequences of climate change in interprofessional education: A scoping review | no literacy or competence concept |
| McLean, Michelle; Phelps, Charlotte; Smith, Jessica; Maheshwari, Neelam; Veer, Vineesha; Bushell, Dayna; Matthews, Richard; Craig, Belinda; Moro, Christian | 2022 | An authentic learner-centered planetary health assignment: A five-year evaluation of student choices to address Sustainable Development Goal 13 (Climate Action) | no literacy or competence concept |
| Mengi Çelik, Özge; Köksal, Eda; Akpinar, Şerife; Kocaadam Bozkurt, Betül; Erdoğan Gövez, Nazlıcan; Çıtar Dazıroğlu, Merve Esra; Karaçil Ermumcu, Merve Şeyda; Acar Tek, Nilüfer | 2024 | Association between ecological footprint awareness and health literacy in adults: a population-based study from Turkey | wrong population |
| Miller, Mark D.; Valenti, Maria; Schettler, Ted; Tencza, Brian | 2016 | A Story of Health: Filling a Gap in Environmental Health Literacy for Health Professionals | wrong type of sources |
| Moloo, Husein; Selvam, Rajajee; Seguin, Nieve; Zhang, Lisa; Lacaille-Ranger, Ariane; Sikora, Lindsey; McIsaac, Daniel I. | 2022 | Integration of planetary health in undergraduate and postgraduate medical education: protocol for a scoping review | no literacy or competence concept |
| Moro, Christian; McLean, Michelle; Phelps, Charlotte | 2023 | Embedding planetary health concepts in a pre-medical physiology subject | no literacy or competence concept |
| Moser, J. Michael | 2008 | Core academic competencies for master of public health students: One health department practitioner’s perspective | no literacy or competence concept |
| Nicole, Wendee | 2024 | PERHLs of Wisdom: Environmental Reproductive Health Literacy and Phthalates | wrong population |
| Nikendei, Christoph; Kühl, Susanne J.; Bugaj, Till Johannes | 2023 | The climate crisis and planetary health in teaching | no literacy or competence concept |
| Nordrum, Ola Løkken; Kirk, Aoife; Lee, Sadhbh A.; Haley, Kathryn; Killilea, David; Khalid, Iffat; Pérez, Esmeralda Cecilie; Waters, Isabel; Yan, Ruth; Zulfiqar, Sana; Stanistreet, Debbi | 2022 | Planetary health education in medical curricula in the Republic of Ireland | no literacy or competence concept |
| Ogunseitan, Oladele A. | 2022 | Broad spectrum integration of climate change in health sciences curricula | no literacy or competence concept |
| Okatch, H.; Remshifski, P. A.; Fennessey, A.; Campbell, H.; Barnoy, S.; Friedman, J.; Kern, S. B.; Frasso, R.; Sorensen, C.; Bar-Shalita, T.; Hunter, L. N. | 2024 | Climate change and its impact on health: a global collaborative learning model | no literacy or competence concept |
| Olson, D.; Stedman-Smith, M.; Fredrickson, A. | 2005 | Environmental health and nursing: piloting a technology-enhanced distance learning module | no literacy or competence concept |
| Örs, Mukaddes | 2022 | A Measurement of Environmental Literacy of Nursing Students for Sustainable Environment | no health aspects linking literacy or competence |
| Ortega, Luis Martin; Westerhaus, Michael J.; Finnegan, A. M.Y.; Bhatt, AaartiI; Owilli, Alex Olirus | 2023 | Toward an Integrated Framework in Health and Human Rights Education: Transformative Pedagogies in Social Medicine, Collective Health, and Structural Competency | no literacy or competence concept |
| Ortner, Pamela M. | 2004 | The nurse as change agent: an approach to environmental health advocacy training | no literacy or competence concept |
| Ossebi, W.; Ndjoug, Ndour, AP; Dieng, S. D.; Bedekelabou, A. P.; Kalandi, M.; Diop, F. N.; Bada, Alambedji, R; Kaboret, Y. Y.; Faye, A.; Sambou, B. | 2022 | One Health training needs for Senegalese professionals to manage emerging public health threats | no literacy or competence concept |
| Oudbier, Janique; Sperna Weiland, Nicolaas H.; Boerboom, Tobias; Ravesloot, Jan Hindrik; Peerdeman, Saskia; Suurmond, Jeanine | 2022 | An evidence-based roadmap to integrate planetary health education into the medical curriculum | no literacy or competence concept |
| Pais Rodrigues, Catarina; Papageorgiou, Evangelos; McLean, Michelle | 2024 | Development of a suite of short planetary health learning resources by students for students as future health professionals | no literacy or competence concept |
| Parker, Gillian; Berta, Whitney; Shea, Christine; Miller, Fiona | 2020 | Environmental competencies for healthcare educators and trainees: A scoping review | no literacy or competence concept |
| Patrick, Rebecca; Bruges, Nicole; Gunasiri, Hasini; Wang, Yifan; Henderson-Wilson, Claire | 2025 | Healthy Me, Healthy Planet: Evaluation of a pilot planetary health library program | wrong population |
| Pattnaik, Anandita; Vaz, Manjulika | 2024 | Urgent need for planetary health education for healthcare professionals and students in India | no literacy or competence concept |
| Perán, Ángeles Navarro | 2023 | La formación específica en sostenibilidad y medio ambiente en el personal sanitario | foreign language |
| Perreault-Carranza, T.; Ni, V.; Savoie, J.; Saucier, J.; Frenette, J.; Jbilou, J. | 2024 | Core Competencies of the Public Health Workforce in Climate Change and Extreme Weather Events Preparedness, Response, and Recovery: A Scoping Review. [Review] | no literacy or competence concept |
| Pettinger, C. | 2018 | Sustainable eating: Opportunities for nutrition professionals | no literacy or competence concept |
| Pettinger, Clare; Tripathi, Smita; Shoker, Benji; Hodge, Gary | 2023 | Collaborative leadership to support sustainability in practice for dietitians as allied health professionals | no literacy or competence concept |
| Philipsborn, Rebecca Pass; Sheffield, Perry; White, Andrew; Osta, Amanda; Anderson, Marsha S.; Bernstein, Aaron | 2021 | Climate Change and the Practice of Medicine: Essentials for Resident Education | no literacy or competence concept |
| Piccardo, Chiara; Goto, Yutaka; Koca, Deniz; Aalto, Pasi; Hughes, Mark | 2022 | Challenge-based, interdisciplinary learning for sustainability in doctoral education | no literacy or competence concept |
| Portela Dos Santos, Omar; Perruchoud, Élodie; Pereira, Filipa; Alves, Paulo; Verloo, Henk | 2024 | Measuring Nurses’ Knowledge and Awareness of Climate Change and Climate-Associated Diseases: Systematic Review of Existing Instruments | no health aspects linking literacy or competence |
| Potter, Teddie | 2019 | Planetary Health: The Next Frontier in Nursing Education | no literacy or competence concept |
| Qi, Shaojie; Hua, Fengrui; Xu, Shengyuan; Zhou, Zheng; Liu, Feng | 2021 | Trends of global health literacy research (1995-2020): Analysis of mapping knowledge domains based on citation data mining | no environmental focus |
| Quinn Griffin, Mary T.; Alfes, Celeste M.; Chavez, Freida; Ea, Emerson E.; Lynn, Kelly A.; Rafferty, Margaret A.; Fitzpatrick, Joyce J. | 2022 | Incorporating climate change into Doctor of Nursing Practice curricula | no literacy or competence concept |
| Quitmann, Claudia; Griesel, Silvan; Nayna Schwerdtle, Patricia; Danquah, Ina; Herrmann, Alina | 2023 | Climate-sensitive health counselling: a scoping review and conceptual framework | no literacy or competence concept |
| Rabinowitz, Peter M.; Natterson-Horowitz, Barbara J.; Kahn, Laura H.; Kock, Richard; Pappaioanou, Marguerite | 2017 | Incorporating one health into medical education | no literacy or competence concept |
| Ramirez-Andreotta, Monica | 2016 | Advancing Informal Environmental STEM Literacy & Learning: A Co-Created Citizen Science Rainwater Harvesting in Underserved Communities | wrong population |
| Ramirez-Andreotta, Monica D.; Buxner, Sanlyn; Sandhaus, Shana | 2023 | Co-created environmental health science: Identifying community questions and co-generating knowledge to support science learning | wrong population |
| Redvers, Nicole; Celidwen, Yuria; Schultz, Clinton; Horn, Ojistoh; Githaiga, Cicilia; Vera, Melissa; Perdrisat, Marlikka; Mad Plume, Lynn; Kobei, Daniel; Kain, Myrna Cunningham; Poelina, Anne; Rojas, Juan Nelson; Blondin, Be’sha | 2022 | The determinants of planetary health: an Indigenous consensus perspective | literacy or competence concept |
| Rogers, Bonnie; McCurdy, Leyla Erk; Slavin, Katie; Grubb, Kimberly; Roberts, James R. | 2009 | Children’s Environmental Health Faculty Champions Initiative: A Successful Model for Integrating Environmental Health into Pediatric Health Care | no literacy or competence concept |
| Rosa, William E.; Upvall, Michele J. | 2019 | The case for a paradigm shift: from global to planetary nursing | no literacy or competence concept |
| Ross, Jean; Speirs, Jo | 2024 | Championing a move from sustainability to Planetary Health in nursing curriculum | no literacy or competence concept |
| Rusaanes, Veronica; Eide, Hilde; Brembo, Espen Andreas; Gladhus, Lise; Oswald, Birte Marten; Heyn, Lena Günterberg | 2024 | Educating nursing students for sustainable future rural health-care services: An umbrella review | no literacy or competence concept |
| Sá, Patricia; Lourenco, Monica; Carlos, Vania | 2022 | Sustainability Competencies in Higher Education Research: An Analysis of Doctoral Theses in Portugal | no literacy or competence concept |
| Samiee Roudi, Khaled; Peyman, Nooshin | 2019 | Understanding the Concept of Health Literacy by Health Personnel: A Qualitative Content Analysis | foreign language |
| Saunders, Carla; Palesy, Debra; Lewis, Joanne | 2019 | Systematic Review and Conceptual Framework for Health Literacy Training in Health Professions Education | no environmental focus |
| Sawleshwarkar, Shailendra; Negin, Joel | 2017 | A Review of Global Health Competencies for Postgraduate Public Health Education | no literacy or competence concept |
| Schmid, Joerg; Mumm, Anna; Koenig, Sarah; Zirkel, Janina; Schwienhorst-Stich, Eva-Maria | 2023 | Concept and implementation of the longitudinal mosaic curriculum planetary health at the Faculty of Medicine in Wurzburg, Germany | no literacy or competence concept |
| Schmitz, Daniela; Fiedler, Manfred; Ortloff, Jan-Hendrik | 2024 | Lehrkonzept für die Pflege zu Vulnerabilität und Klima | no literacy or competence concept |
| Schwerdtle, Patricia Nayna; Maxwell, Janie; Horton, Graeme; Bonnamy, James | 2020 | ’12 tips for teaching environmental sustainability to health professionals’ | no literacy or competence concept |
| Schwienhorst-Stich, E. M.; Kropff, D.; Kersken, K.; Konig, S.; Leutritz, T.; Parisi, S.; Schlittenhardt, C.; Schmid, J.; Simmenroth, A.; Junger, J.; Zirkel, J. | 2024 | The elective planetary health: Climate, environment and health at the faculty of medicine in Wurzburg: Concept, didactic methods and evaluation results | Duplicate in other language |
| Schwienhorst-Stich, Eva-Maria; Kropff, Dana; Kersken, Katharina; König, Sarah; Leutritz, Tobias; Parisi, Sandra; Schlittenhardt, Clara; Schmid, Jörg; Simmenroth, Anne; Jünger, Jana; Zirkel, Janina | 2024 | Das Wahlpflichtfach Planetare Gesundheit: Klima. Umwelt. Gesundheit an der Medizinischen Fakultät Würzburg ‐ Konzeption, didaktische Methoden und Evaluationsergebnisse | no literacy or competence concept |
| Schwienhorst-Stich, Eva-Maria; Wabnitz, Katharina; Geck, Eva; Gepp, Sophie; Jung, Laura; Mumm, Anna; Schmid, Jörg; Simmenroth, Anne; Simon, Johanna; Eichinger, Michael | 2023 | Initiatives promoting planetary health education in Germany: An overview | no literacy or competence concept |
| Selvam, R.; Seguin, N.; Zhang, L.; Lacaille-Ranger, A.; Sikora, L.; Raiche, I.; McIsaac, D. I.; Moloo, H. | 2024 | International Planetary Health Education in Undergraduate and Graduate Medical Curricula: A Scoping Review. [Review] | no literacy or competence concept |
| Sharma, Atul; Smyth, Lillian; Jian, Holly; Vargas, Nicole; Bowles, Devin; Hunter, Arnagretta | 2024 | Are we teaching the health impacts of climate change in a clinically relevant way? A systematic narrative review of biomechanism-focused climate change learning outcomes in medical curricula | no literacy or competence concept |
| Shaw, Emily; Walpole, Sarah; McLean, Michelle; Alvarez-Nieto, Carmen; Barna, Stefi; Bazin, Kate; Behrens, Georgia; Chase, Hannah; Duane, Brett; El Omrani, Omnia; Elf, Marie; Faerron Guzmán, Carlos A.; Falceto de Barros, Enrique; Gibbs, Trevor J.; Groome, Jonny; Hackett, Finola; Harden, Jeni; Hothersall, Eleanor J.; Hourihane, Maca; Huss, Norma May; Ikiugu, Moses; Joury, Easter; Leedham-Green, Kathleen; MacKenzie-Shalders, Kristen; Madden, Diana Lynne; McKimm, Judy; Nayna Schwerdtle, Patricia; Peters, Sarah; Redvers, Nicole; Sheffield, Perry; Singleton, Judith; Tun, SanYuMay; Woollard, Robert | 2021 | AMEE Consensus Statement: Planetary health and education for sustainable healthcare | no literacy or competence concept |
| Sibindi, T.; Chipps, J. A.; Crowley, T. | 2024 | Eco-nursing competencies for nurses: A scoping review. [Review] | no literacy or competence concept |
| Simon, Johanna; Parisi, Sandra; Wabnitz, Katharina; Simmenroth, Anne; Schwienhorst-Stich, Eva-Maria | 2023 | Ten characteristics of high-quality planetary health education-Results from a qualitative study with educators, students as educators and study deans at medical schools in Germany | no literacy or competence concept |
| Singer-Brodowski, Mandy; Henkel, Gianna-Maria; Reith, Alexandra; Frank, Pascal; Rieckmann, Marco | 2025 | What is needed to act as a professional change agent for sustainability? A scoping review | no literacy or competence concept |
| Sorensen, C.; Magalhaes, D.; Hamacher, N.; Sullivan, J. K.; Weinstein, H. N.W.; Pinho-Gomes, A. C.; Biberman, D.; Donaldson, H.; Gomez-Duarte, I.; Middleton, J.; Magana, L.; Urbina, M.; Kaseje, M.; Cascante-Flores, N.; Surenthirakumaran, R.; Ivers, R.; Saenz, R.; Chen, T. T.; Lopez, W.; Romanello, M.; Zhang, Y. | 2024 | Climate and health education in public health schools worldwide during 2023-24: a survey | wrong population no |
| Sorensen, C. J.; Fried, L. P. | 2024 | Defining Roles and Responsibilities of the Health Workforce to Respond to the Climate Crisis | no literacy or competence concept |
| Sorensen, Cecilia; Campbell, Haley; Depoux, Anneliese; Finkel, Madelon; Gilden, Robyn; Hadley, Kristie; Haine, Dana; Mantilla, Gilma; McDermott-Levy, Ruth; Potter, Teddie M.; Sack, Todd L.; Tun, SanYuMay; Wellbery, Caroline | 2023 | Core competencies to prepare health professionals to respond to the climate crisis | no literacy or competence concept |
| Spooner, Rosie; Clack, Alice; Parslow Williams, Siobhan; Mortimer, Frances | 2023 | Empowering students and health workers to take action on climate change | no literacy or competence concept |
| Stojkov, Svetlana; Tadic, Ivana; Crnjanski, Tatjana; Krajnovic, Dusanka | 2016 | Assessment and self-assessment of the pharmacists’ competencies using the Global Competency Framework (GbCF) in Serbia | no literacy or competence concept |
| Stoliarchuk, Olesia; Liang, Keyan; Khrypko, Svitlana; Ishchuk, Olena; Serhieienkova, Oksana; Kalishchuk, Svitlana; Divchuk, Tetiana | 2023 | Bridging Education and Sustainable Development: Assessing Future Psychologists’ Perspectives | no literacy or competence concept |
| Stone, Sara B.; Myers, Samuel S.; Golden, Christopher D. | 2018 | Cross-cutting principles for planetary health education | no literacy or competence concept |
| Sullivan, James K.; Lowe, Katherine E.; Gordon, Ilyssa O.; Colbert, Colleen Y.; Salas, Renee N.; Bernstein, Aaron; Utech, Jon; Natowicz, Marvin R.; Mehta, Neil; Isaacson, J. Harry | 2022 | Climate Change and Medical Education: An Integrative Model | no literacy or competence concept |
| Swärdh, Emma; Brodin, Nina; Pettersson, Anna; Palstam, Annie | 2024 | Time to Rethink Intended Learning Outcomes for Sustainable Development? A Qualitative Exploration and Reflection of Course Syllabuses in Swedish Undergraduate Physiotherapy Education | no literacy or competence concept |
| Tagtow, A.; Robien, K.; Bergquist, E.; Bruening, M.; Dierks, L.; Hartman, B. E.; Robinson-O’Brien, R.; Steinitz, T.; Tahsin, B.; Underwood, T.; Wilkins, J. | 2014 | Academy of Nutrition and Dietetics: Standards of professional performance for Registered Dietitian Nutritionists (Competent, Proficient, and Expert) in Sustainable, Resilient, and Healthy Food and Water Systems | no literacy or competence concept |
| Teherani, A.; Nicastro, T.; Clair, M. S.; Nordby, J. C.; Nikjoo, A.; Collins, S.; Irani, A.; Zakaras, J.; Weiser, S. D. | 2023 | Faculty Development for Education for Sustainable Health Care: A University System-Wide Initiative to Transform Health Professional Education | no literacy or competence concept |
| Teherani, A.; Nishimura, H.; Apatira, L.; Newman, T.; Ryan, S. | 2017 | Identification of core objectives for teaching sustainable healthcare education | no literacy or competence concept |
| Togami, E.; Behravesh, C. B.; Dutcher, T. V.; Hansen, G. R.; King, L. J.; Pelican, K. M.; Mazet, J. A.K. | 2023 | Characterizing the One Health workforce to promote interdisciplinary, multisectoral approaches in global health problem-solving | no literacy or competence concept |
| Tsakonas, Kiera; Badyal, Simi; Takaro, Tim; Buse, Chris G. | 2024 | Rapid review of the impacts of climate change on the health system workforce and implications for action | no literacy or competence concept |
| Tu’itahi, Sione; Watson, Huti; Egan, Richard; Parkes, Margot W.; Hancock, Trevor | 2021 | Waiora: the importance of Indigenous worldviews and spirituality to inspire and inform Planetary Health Promotion in the Anthropocene | wrong population |
| Tucker, C.; Keyel, J.; Blue, A.; Chun, R.; Estrada, A.; Khalili, H.; Riles, A.; Roopnarine, R.; Sick, B.; VandeWoude, S.; Watson, B.; Wisnieski, L.; Webb, T. | 2024 | The intersection of Interprofessional Education and One Health: A qualitative study in human and veterinary medical institutions | no literacy or competence concept |
| Upvall, Michele J.; Luzincourt, Geraldine | 2019 | Global citizens, healthy communities: Integrating the sustainable development goals into the nursing curriculum | no literacy or competence concept |
| Valentine, Nicole; Ajuebor, Onyema; Fisher, Julian; Bodenmann, Patrick; Baum, Fran; Rasanathan, Kumanan | 2022 | Planetary health benefits from strengthening health workforce education on the social determinants of health | no literacy or competence concept |
| Valois, Pierre; Blouin, Patrick; Ouellet, Claudine; Renaud, Jean-Sebastien; Belanger, Diane; Gosselin, Pierre | 2016 | The Health Impacts of Climate Change: A Continuing Medical Education Needs Assessment Framework | full text not available |
| Villagran, Melinda; Weathers, Melinda; Keefe, Brian; Sparks, Lisa | 2010 | Medical Providers as Global Warming and Climate Change Health Educators: A Health Literacy Approach | no environmental focus |
| Vold, Lindsey; Wallar, Lauren; Papadopoulos, Andrew | 2022 | An evolution in health professional education: the ecological determinants of health | wrong population |
| Voşki, Anaïs; Wong-Parodi, Gabrielle; Ardoin, Nicole M. | 2024 | Eco-emotions as the planetary boundaries: framing human emotional and planetary health in the global environmental crisis | no literacy or competence concept |
| Wabnitz, K.; Galle, S.; Hegge, L.; Masztalerz, O.; Schwienhorst-Stich, E. M.; Eichinger, M. | 2021 | Planetary health-transformative education regarding the climate and sustainability crises for health professionals | duplicate in other language |
| Wabnitz, Katharina; Galle, Sophia; Hegge, Louise; Masztalerz, Oskar; Schwienhorst-Stich, Eva-Maria; Eichinger, Michael | 2021 | Planetare Gesundheit ‐ transformative Lehr- und Lernformate zur Klima- und Nachhaltigkeitskrise für Gesundheitsberufe | no literacy or competence concept |
| Wabnitz, Katharina; Schwienhorst-Stich, Eva-Maria; Asbeck, Franziska; Fellmann, Cara Sophie; Gepp, Sophie; Leberl, Jana; Mezger, Nikolaus Christian Simon; Eichinger, Michael | 2022 | National Planetary Health learning objectives for Germany: A steppingstone for medical education to promote transformative change | no literacy or competence concept |
| Walpole, S.; Popat, A.; Blakey, E. P.; Holden, E.; Whittaker, B.; Saggu, R.; Fennell-Wells, A.; Armit, K.; Hothi, D. | 2024 | Health and social care professional standards need to be updated to advance leadership and action for environmental sustainability and planetary health | no literacy or competence concept |
| Walpole, Sarah Catherine; Barna, Stefi; Richardson, Janet; Rother, Hanna-Andrea | 2019 | Sustainable healthcare education: integrating planetary health into clinical education | no literacy or competence concept |
| Walpole, Sarah Catherine; Pearson, David; Coad, Jonathan; Barna, Stefi | 2016 | What do tomorrow’s doctors need to learn about ecosystems? - A BEME Systematic Review: BEME Guide No. 36 | no literacy or competence concept |
| Weatherford, Barbara; Bower, Kathleen A.; Vitello-Cicciu, Joan | 2018 | The CNO and Leading Innovation: Competencies for the Future | no environmental focus |
| White, Brandi M.; Hall, Eric S.; Johnson, Cheryl | 2014 | Environmental Health Literacy in Support of Social Action: An Environmental Justice Perspective | wrong population |
| Whitmee, Sarah; Haines, Andy; Beyrer, Chris; Boltz, Frederick; Capon, Anthony G.; Souza Dias, Braulio Ferreira de; Ezeh, Alex; Frumkin, Howard; Gong, Peng; Head, Peter; Horton, Richard; Mace, Georgina M.; Marten, Robert; Myers, Samuel S.; Nishtar, Sania; Osofsky, Steven A.; Pattanayak, Subhrendu K.; Pongsiri, Montira J.; Romanelli, Cristina; Soucat, Agnes; Vega, Jeanette; Yach, Derek | 2015 | Safeguarding human health in the Anthropocene epoch: report of The Rockefeller Foundation-Lancet Commission on planetary health | no literacy or competence concept |
| Wihlborg, Monne; Avery, Helen | 2021 | Global Health in Swedish Nursing Curricula: Navigating the Desirable and the Necessary | no literacy or competence concept |
| Wong, D.; Kogan, L. R. | 2013 | Veterinary students’ attitudes on One Health: implications for curriculum development at veterinary colleges | no literacy or competence concept |
| Worthington, S.; Armstrong, K.; Debevec, E. | 2010 | The green choices project: integrating environmental health education into reproductive health care settings | no literacy or competence concept |
| Xie, Edward; Barros, Enrique Falceto de; Abelsohn, Alan; Stein, Airton Tetelbom; Haines, Andy | 2018 | Challenges and opportunities in planetary health for primary care providers | no literacy or competence concept |
| Yu, Ji Hye; Hwa, Park Kwi | 2024 | Challenges for Sustainable Interprofessional Education in South Korea: Insights from Key Global Countries: 지속 가능한 국내 전문직 간 교육 발전을 위한 과제: 세계 주요 국가 사례를 중심으로 | foreign language |
| Zandavalli, Rafaela Brugalli; Stein, Airton Tetelbom; Camargo, Tatiana Souza de | 2024 | Systems approach in planetary health education for medical students: a mixed methods study | no literacy or competence concept |

# **Additional** **Table 5:** **Overview of the included studies and their main characteristics**

| Reference (First author, year) | Country | Main affiliation / discipline of the authors | Methodological approach | Target group | Environmental focus | Literacy / competence concept | Study objectives |
| --- | --- | --- | --- | --- | --- | --- | --- |
| (Albrecht et al., 2023) | Germany | Medicine, Medical Sociology | Qualitative study | Health professionals (physicians and nurses) from various clinical specialties | Planetary health | Climate-specific health literacy | Assess knowledge regarding climate change and its impacts on health and climate-specific health literacy in health professionals. |
| (Atta et al., 2024) | Egypt | Medical‑Surgical, Psychiatric & Mental Health, Family & Community Health, Administration, Critical Care & Emergency and Education in context of Nursing | Quantitative study | Nursing students | Climate change | Climate change literacy | Identify the predictors of climate change literacy among nursing students. |
| (Barna et al., 2020) | UK, Norway, India, Canada, Netherlands | Sustainable Healthcare, Public Health, Environmental Physiotherapy, Medicine, Teaching and Education | Conceptual analysis and framework | Health professional students (medical and nursing) | Planetary health | Planetary health learning outcomes and competences for health profession education | Provides suggestions for planetary health learning outcomes to equip health profession education graduates with the knowledge, skills and values they need to build a sustainable healthcare system. |
| (Baungaard et al., 2023) | UK | Sport & Exercise Sciences, Sustainable Nutrition | Mixed-methods study | Nutrition students | Sustainable diet | Environmental literacy, sustainable food literacy | Ascertain the level of knowledge of sustainable diets, the perceived barriers to their adoption in their own diets, students' experience of university-based teaching about sustainable diets and their views on the future role of the nutrition profession in relation to sustainable diet amongst nutrition students on association for nutrition-accredited degrees. |
| (Bell, 2010) | Australia | Rural Health | Conceptual analysis and discussion paper | Health practitioners (especially rural and remote health practitioners) | Climate change | Environmental literacy for climate change , eco-medical literacy | Explore the nature of key competences and related opportunities for teaching climate change in medical education and training. |
| (Binder et al., 2022) | USA | Human Health & Environment, Communication, Health & Equity | Quantitative survey study | General adult population | Environmental health | Environmental health literacy | Investigate the relationships between environmental health literacy, the characteristics of people (race, ethnicity, and socioeconomic status) associated with health disparities, and people’s willingness to engage in protective behaviors against environmental health threats. |
| (Blankart et al., 2024) | Switzerland, UK, Germany, Ireland | Public Management and Policy, Translational and Entrepreneurial Medicine, Infectious Diseases, Health Literacy, Health & Social Care, Population Health, Psychiatry, Nursing, Digital Health, Business | Conceptual analysis and framework | Health policy, governance, and leadership in One Health | One health | One health literacy, one health governance | Investigate how the implementation of one health can be supported by the concepts of one health literacy and one health governance. Show how one health literacy and one health governance influence and reinforce each other.  Describe a model that can be used to investigate how external triggering events can set a positive cycle in motion. |
| (Brugger et al., 2024) | Austria | Climate Resilience & One Health | Mixed-methods study | Health professionals | Climate change | Health-related climate literacy | Building a common understanding and developing an adequate content framework for a climate-smart healthcare workforce. |
| (Brugger and Horváth, 2024) | Austria | Climate Resilience, One Health and Care | Mixed-methods study | Health professionals, students, practitioners, and educators | Climate change | Health-related climate literacy | Develop a sound definition of the term health-related climate literacy. Describe the status quo and the necessary literacy development in health and care professions. Integration of health-related climate literacy in education in Austria. |
| (Davis et al., 2018) | USA | Teaching, Learning & Sociocultural Studies, Environmental Science, Health Promotion Sciences, Chemistry & Biochemistry | Mixed-methods study | Communities with known environmental health stressors in Arizona, USA | Environmental health | Environmental health literacy | Increasing Environmental Health Literacy through contextual learning in communities with known Environmental Health stressors in Arizona, USA. |
| (Finn and O'Fallon, 2017) | USA | Environmental Health Sciences, Health & Human Resources | Conceptual analysis and framework | Public health, Environmental Health scientists and community health educators | Environmental health | Environmental health literacy | Stimulate a conversation on, and advance research in, environmental health literacy . |
| (Fiore et al., 2024) | Italy | Medical, Surgical & Advanced Technologies, Health Literacy, Hygiene & Preventive Medicine, Biology | Qualitative study | University students from different fields of specialisation | Environmental health | Environmental health literacy | Fill this gap through the validation of the Environmental Health Literacy Index (EHLI) of university students. |
| (Grabow et al., 2023) | USA | Medicine, Public Health, Natural Resources, Sustainability, Global Environment & Health Studies | Conceptual analysis and framework | Learners: students, practicing professionals in the current formal workforce, and other interested individuals | Climate change | Climate literacy, climate change literacy, climate health literacy | Explore tangible opportunities to strengthen climate and health understanding among individuals spanning educational levels, professional settings, and societal needs. |
| (Jacobsen et al., 2024) | USA, South Africa, UK, Monaco, Australia, China, | Global Health, Health, Natural &Environmental Sciences, Health Systems & Public Health, Planetary Health, Health Policy & Organization, Scientific, Epidemiology & Environmental Health, Climate & Health Education, Global Citizenship, International Health | Conceptual analysis and framework | Educators and health professional learners | Planetary health | Planetary health learning objectives and competences | Develop a set of planetary health learning objectives that enable learners already familiar with foundational global health principles to gain crucial knowledge about planetary health science, interventions, and communication. |
| (Jagals and Ebi, 2021) | Australia, USA | Children’s Health & Environment, Global Health, Environmental & Occupational Health | Conceptual analysis and framework | Health workforce, health/medical trainers | Climate and environmental health | Climate and environmental health competences | Develop a climate and environmental health core competence framework for health workers. |
| (Jochem et al., 2022) | Germany | Epidemiology and Preventive Medicine, Development & Sustainability, Political Sciences & Sociology, General Practice & Family Medicine, Medical Teaching & Education, Social Medicine & Health Systems, Clinical Sciences Building | Conceptual analysis and framework | Specialists from the fields of public health, environmental science, education and policy-making | Planetary health | Planetary health literacy | Develop a conceptual model of planetary health literacy and necessary core competences. |
| (Jochem et al., 2024) | Germany, Singapore | Planetary & Public Health, Social Medicine & Health Systems, Development & Sustainability, Political Sciences & Sociology, Climate and Planetary Health, Clinical Sciences Building | Conceptual analysis and framework | Policy makers, health professionals, educators, general public | Planetary health | Planetary health literacy | Discuss the concept of planetary health literacy as an educational goal and the role of policymakers and health professionals in this context. |
| (Levett-Jones et al., 2024) | Australia | Science Medicine & Health, Sustainable Futures, Innovative Solutions for Well-Being & Health, Nursing, Health, Medicine & Wellbeing | Qualitative study | Nursing students | Planetary health | Planetary health knowledges and skills | To achieve consensus on the knowledge and skills that undergraduate/pre-licensure nursing students require to steward healthcare towards a more sustainable future. |
| (Lichtveld et al., 2019) | USA | Environmental Health Sciences, Biostatistics & Data Science | Quantitative study | Public health students, community members | Environmental health | Environmental health literacy | Developed a validated survey instrument with four scales for assessing media-specific (i.e., air, food, water) and general environmental health literacy. |
| (Limaye et al., 2020a) | USA | Natural Resources, Family Medicine & Community Health, Global Health, Population Health | Conceptual analysis and framework | students, educators, health professionals and the broader public | Environmental health | Climate health literacy | Propose a definition of climate health literacy and its corresponding set of elements. Assess how peer-reviewed literature addresses these elements. Develop a framework that builds on these elements to support a broader educational agenda, preparing students and future leaders to recognise health consequences of climate change. |
| (Lindsey et al., 2021) | USA | Environmental Health, Quality & Safety | Mixed-methods study | Environmental Health professionals, environmental public health educators, and users of Environmental Health information in the public | Environmental health | Environmental health literacy | Determine knowledge and skill items identified by environmental health professionals as being associated with environmental health literacy and to understand how these items rank by importance. |
| (Lopez-Medina et al., 2019) | Spain, UK, Germany, Netherlands | Nursing, Health & Human Sciences, Social Work, Heath & Care, Global Health | Conceptual analysis and framework | Nursing students | Climate change | Environmental health competences | Present a review of literature that focuses on the environmental health competences required and pedagogic approaches used to embed sustainability in curricula in nursing education. |
| (Lynch et al., 2024) | Australia, Canada | Medicine & Health, Public Health, Health Environments and Lives, Health Policy, Management & Evaluation, Climate, Health & Sustainable Care, Critical Care, Anaesthesia & Intensive Care | Qualitative study | Clinicians from speciality areas | Climate change | Carbon health literacy | Explore clinicians’ views on carbon health literacy to understand how knowledge, skills and capacities related to the emissions of clinical care has application in clinical practice. |
| (Maxwell and Blashki, 2016) | Australia | Global health | Conceptual analysis and framework | Medical students and doctors | Climate change | Eco-medical literacy | Argue why climate change should be integrated into medical education.  Assist universities to integrate teaching about climate change into medical education. |
| (Moghbeli et al., 2024) | Iran | Nursing & Medical Education, Health Management & Safety Promotion | Conceptual analysis and framework | Nursing students and Nurses | Ecology | Ecological care | Analyze and clarify the concept of ecological care in the nursing discipline. |
| (Raile, 2023) | Austria | Psychotherapy Science | Conceptual analysis and framework | Psychotherapists | Climate change | Climate health literacy | Make a scientifically based plea for greater consideration of the psychological impacts related to climate change in the education and training of health professionals. |
| (Reismann et al., 2021) | Germany | Epidemiology & Preventive Medicine | Quantitative study | Patients of general practitioners and gynecologists | Planetary health | Climate-specific health literacy | Investigate the current state of climate-specific health literacy in patients and the impact of climate-specific medical advice on patients’ behavior regarding health co-benefits. |
| (Rosenau et al., 2024) | Germany, USA | Nutrition & Psychology, Culinary Medicine, Teaching Kitchen | Mixed-methods study | University students | Planetary health | Planetary health diet | Introduce the planetary health diet curriculum and explain the theoretical framework in which it is embedded. Assess the practical feasibility of the course concept in higher education. Examine the effect of the planetary health diet curriculum on university students' ability to advise individuals in their professional or personal environment on more sustainable or healthier diets, referred to as planetary health diet literacy. |

# **Additional Table 6:** **Definition and determinants of included literacy and competence concepts**

| **References** | **Literacy / competence concept** | **Definition of the literacy /competence concept and theoretical foundation** | **Determinants of literacy / competence concept** |
| --- | --- | --- | --- |
| **(Albrecht et al., 2023)** | Climate-specific health literacy | Climate-specific health literacy encompasses knowledge about the present and long-term health risks of climate change and knowledge about the health co-benefits of health promoting behaviors related to climate change. Moreover, the concept includes emotional integration of knowledge and feelings of concern related to climate change and health and the ability to implement this knowledge into action and climate-friendly behavior (Reismann et al., 2021). | Demographic characteristics (gender, age, job, medical discipline); self-assessed awareness and knowledge of climate change in general, emotional integration of knowledge and feelings of concern related to climate change and health, the ability to implement this knowledge into action and willingness for climate friendly behavior. |
| **(Atta et al., 2024)** | Climate change literacy | Aspects of climate literacy are knowledge of climate science, health impacts, adaptation and mitigation strategies, and communication and advocacy skills (Ergin et al., 2021). | Climate literacy domains: knowledge of fundamental climate science principles and health risks, awareness of adaptation and mitigation strategies, communication and advocacy in addressing climate-related challenges Geographic diversity and socio‑demographics (regional affiliations, demographic characteristics like age, sex, income, and parental education), health status and habits (dietary choices, physical exercise routines, sleep patterns, chronic diseases, smoking habits), faculty knowledge, and institutional support. |
| **(Barna et al., 2020)** | Planetary health learning objectives and competences | Situates health within a broader understanding of the interdependent socio-ecological drivers of human and planetary health. It facilitates novel ways of protecting both population health and the natural environment on which human health and wellbeing depends (Walpole et al., 2015). | Values: recognize professional duty for environmental stewardship as part of the ethical principle of beneficence; support national and professional bodies to set guidance on healthcare practices, and commit to practices which simultaneously address equity, and sustainability in health.  Knowledge**:** integrate planetary health concepts (i.e. socio-ecological determinants of health) across the curriculum; identify how to prevent the harmful effects of global environmental changes in ways that integrate culturally relevant knowledge practices.  Skills: demonstrate clinical, leadership, and advocacy competences for environmental stewardship of the health system; identify research questions which address both equity, and sustainability in mitigation and adaptation. |
| **(Baungaard et al., 2023)** | Environmental literacy and sustainable food literacy | Environmental literacy refers to the capability of making environmentally responsible behaviors and involves an individual's understanding and motivation to make environmentally responsible decisions. Sustainable food literacy refers to the capability of making sustainable food choices. The environmental literacy components model suggests that higher levels of environmental knowledge tend to increase personal responsibility, and sustainable behaviors (Gerber, P.J., Steinfeld, H., Henderson, B., Mottet, A., Opio, C., Dijkman, J., Falcucci, A. & Tempio, G., 2013; Macdiarmid, 2013; Brent Kim et al., 2015; Innes et al., 2018; Springmann et al., 2018). | Demographic data (university level, entry qualifications, age, sex, degree of sustainability education and environmental organization membership), knowledge of sustainable diets, attitude, capability of making environmentally responsible behavior, capability of making sustainable food choices, eating habits, structural and behavioral barriers, awareness and assumptions, confidence in knowledge or behaviors, understanding, motivation, opinions, education experience. |
| **Bell (2010)** | Environmental literacy for climate change and eco-medical literacy | Environmental literacy can be understood as an occupational and environmental medicine. Environmental medicine for clinical practice defined as a style of medical detective work about the environment, particularly toxins, to improve medical treatment, particularly for chronic conditions and includes the growing subdiscipline of 'ecosystem health', focusing on the ways in which health is shaped by the capacity for ecosystems to be self-sustaining and to carry out all of their normal functions. Environmental literacy for climate change can also be defined as a natural extension of rural and remote medical education and training. The eco-medical literacy that relates to climate change can be defined as the ability to access, understand, integrate and use information about the health-related ecological effects of climate change to deliver and improve medical services (Australian College of Rural and Remote Medicine, 2007). | Knowledge and skills from diverse disciplines with focus on interactions between health, community and place, emergency medical skills, post-disaster management skills, knowledge about population, indigenous health and the underlying mechanisms at work in climate-sensitive health conditions in rural and remote context, professional, legal and ethical practice skills, communication, diagnostic, clinical and procedural skills, the ability to access, understand, integrate and use information about the health-related effects of climate change to deliver and improve medical services. |
| **(Binder et al., 2022)** | Environmental health literacy | Environmental health literacy capturing the continuum between the knowledge of environmental impacts on public health, and the skills and decisions needed to take health-protective actions. Environmental health literacy integrates three separate but complementary concepts drawing from public health, health communication, risk communication, and environmental communication (Dixon et al., 2009; Ramirez-Andreotta et al., 2016; Finn and O'Fallon, 2017; Gray, 2018; Gray and Lindsey, 2019; Lichtveld et al., 2019; Gray et al., 2021). | Factual knowledge (knowing the facts), knowledge sufficiency (feeling ready to decide what to do), and response efficacy (believing that protective behaviors work), demographics (age, sex, education, income, rent home, ethnicity, race), values and beliefs (political ideology, religiosity, trust in research), attention to news (science and health news, politics and government news). |
| **(Blankart et al., 2024)** | One health literacy | One health literacy as the knowledge, motivation and competences to access, understand, appraise, and apply all relevant information that are related to one health order to make judgments, take decisions and actions in everyday life concerning healthcare, disease prevention and health promotion to sustainably balance the health and quality of life of us humans, the animals and the environment. One health governance as the structures and regulations that are designed to promote and support the implementation of the One Health approach. Within the context of one health, systems leadership understand as a leadership style that accounts for the interconnectedness of the human, agricultural and environmental sectors and the ambition for transdisciplinary to achieve a common goal. One health literacy and one health governance as elements that influence and reinforce one another, with systemic leadership as the catalyst reinforcing their interplay (Karesh and Cook, 2009; Sørensen et al., 2012; Décamps et al., 2017; Finn and O'Fallon, 2017; Jochem et al., 2022). | Knowledge, motivation and competences to access, understand, appraise, and apply all relevant information that are related to one health order to make judgments, take decisions and actions. |
| **Brugger et al. (2024)** | Health-related climate literacy | Includes the knowledge and use of synergies between health promotion, climate protection, climate change adaptation and options for action aimed at reducing greenhouse gas emissions. Someone who has health-related climate literacy knows and understands the impact of their own behavior on the climate and the impact of the climate on themselves, the ecosystem and subsequently on society - particularly in terms of physical, mental and social health. In addition, the person also has knowledge of climate change adaptation approaches aimed at not only not exacerbating existing social inequalities, but also reducing them. Furthermore, the person strives to minimize the carbon footprint and increase the carbon handprint (Brugger and Horváth, 2024). | Understand essential principles and the impact of their own behavior on the climate and the impact of the climate on themselves, recognize the direct and indirect links between climate and health, find, understand, evaluate and communicate scientifically sound information on this topic, make informed and responsible decisions for themselves and others. These skills are needed in the areas of (i) self-management, (ii) communication and collaboration, and (iii) increasing the carbon handprint. |
| **(Brugger and Horváth, 2024)** | Health-related climate literacy | Health-related climate literacy involves the ability to understand key principles of the Earth's climate system and recognize how human behavior influences climate and vice versa. Identify direct and indirect connections between climate and health. Access, comprehend, evaluate, and communicate scientific information on climate and health. Make informed decisions that promote both personal and public health while protecting the climate (Sørensen et al., 2012; Austrian Panel on Climate Change, 2018; Adisasmito et al., 2022). Develop strategies for addressing the health impacts of climate change. A person who has sufficient health-related climate literacy therefore knows and understands the influence of their own behavior or health facilities on the climate and the influence of the climate on themselves, on health facilities, the ecosystem and subsequently on society - especially in the area of physical, mental and social health (U.S. Global change Research Program, 2009; Whitmee et al., 2015; Bundesministerium für Arbeit, Soziales, Gesundheit und Konsumentenschutz, 2019; Limaye et al., 2020a). | Understand essential principles and the impact of their own behavior on the climate and the impact of the climate on themselves, recognize the direct and indirect links between climate and health, find, understand, evaluate and communicate scientifically sound information on this topic, make informed and responsible decisions for themselves and others. These skills are needed in the areas of (i) self-management, (ii) communication and collaboration, and (iii) increasing the carbon handprint. |
| **(Davis et al., 2018)** | Environmental health literacy | Environmental health literacy as: (1) environmental science knowledge and awareness related to the community’s specific environmental risks; (2) skills and self-efficacy for learning science, doing science, and environmental action; and, (3) community action for systemic change. Environmental health literacy as a “continuum” of learning that is often tied to active engagement of participants throughout the research process (Zarcadoolas et al., 2001; White et al., 2014; Ramirez-Andreotta et al., 2016; Finn and O'Fallon, 2017; Gray, 2018). | Motivation to learn about environmental health, and attitude towards the environment, environmental science knowledge, skills and motivation for environmental health action, self-efficacy, and workplace action for systemic change, participants’ experiences. |
| **(Finn and O'Fallon, 2017)** | Environmental health literacy | Environmental health literacy begins with an understanding of the link between environmental exposures and health. Environmental health literacy is coalescing into a new subdiscipline that combines key principles and procedural elements from the fields of risk communication, health literacy, environmental health sciences (EHS), communications research, and safety culture. Thes disciplines have contributed unique expertise and perspectives to the development of environmental health literacy. Environmental health literacy integrates concepts from both environmental literacy and health literacy to develop the wide range of skills and competences that people need in order to seek out, comprehend, evaluate, and use environmental health information to make informed choices, reduce health risks, improve quality of life and protect the environment (Minkler et al., 2008; Mogford et al., 2011; Zoller, 2012; Estacio, 2013). | Level of environmental health literacy: recognize, understand, apply, analyze, evaluate, create (as competence levels that build on each other). |
| **(Fiore et al., 2024)** | Environmental health literacy | Environmental health literacy develop the wide range of skills and competences that people need in order to seek out, comprehend, evaluate, and use environmental health information to make informed choices, reduce health risks, improve quality of life and protect the environment (Nutbeam, 2000; Finn and O'Fallon, 2017; Lindsey et al., 2021). | Range of skills and competences that people need in order to seek out, comprehend, evaluate, and use environmental health information to make informed choices, reduce health risks, improve quality of life and protect the environment. Three dimensions of health literacy: functional, interactive, and critical health literacy (build on each other). |
| **(Grabow et al., 2023)** | Climate literacy, climate change literacy and climate health literacy | Climate literacy describes essential principles of climate science and overlays them with basic science literacy benchmarks. Climate literacy also defines one’s ability to assess credible scientific information, communicate clearly, and make informed decisions related to behaviors that impact the climate. No definition for climate change literacy, as it is understood to be synonymous with climate literacy (U.S. Global change Research Program, 2009; Romanello et al., 2023). Climate health literacy: complexity in the relationship between climate change and health via both direct and indirect linkages, as well as the ability to make informed decisions based on such knowledge. Climate health literacy builds on an incomplete characterization of the health impacts of climate change as included in climate change literacy; specifically, it includes understanding of how mitigation and adaptation can reduce harms to physical and mental health, the overlap between fossil fuel dependency and worsening health, as well as the economic costs associated with climate-sensitive health impacts (Crimmins et al., 2016; Limaye et al., 2019; Limaye et al., 2020a; Limaye et al., 2020b). | Assess credible scientific information, communicate clearly, and make informed decisions related to behaviors that impact the climate, understands complexity in the relationship between climate change and health via both direct and indirect linkages, ability to make informed decisions based on such knowledge. understanding of how mitigation and adaptation can reduce harms to physical and mental health, the overlap between fossil fuel dependency and worsening health, as well as the economic costs associated with climate-sensitive health impacts. At three levels of climate health literacy: functional, intermediate, advanced. |
| **(Jacobsen et al., 2024)** | Planetary health learning outcomes and competences | Learning objectives (LOs) and competences are complementary but distinct educational concepts. LOs are specific, focused, measurable statements about what learners will know and be able to do at the end of a class; they typically feature verbs such as compare, explain, and summarize that emphasize factual and conceptual knowledge. Competences define the set of knowledge, skills, and attitudes that learners are expected to achieve across an entire curriculum; they typically feature verbs such as create, design, and demonstrate that emphasize application of procedural knowledge, and they are often focused on preparing students and trainees for success in the workforce. The LOs feature mostly lower-level verbs that describe achievements that learners should be able to demonstrate by the end of an introductory course. The competences, typically require the application of concepts to work in clinical settings. LOs can be attained through classroom-based study, but many of the competences can only be mastered through professional practice (Stone et al., 2018; Guzmán et al., 2021; The Association of Schools and Programs of Public Health, 2022; Redvers et al., 2023; Sorensen et al., 2023; Planetary Health Alliance, 2024). | 8 Planetary health learning outcome domains:  1. Identify Earth system changes  2. Describe Ecological systems  3. Explain Human health outcomes  4. Analyze Risk assessment  5. Evaluate Governance  6. Compare Actions  7. Articulate Ethics  8.Accessing, evaluating, and communicating reliable scientific information’s |
| **Jagals and Ebi (2021)** | Climate, environmental change and health competences | The set of core competences is flexible enough to enable competence in climate, environmental change and health as well as competence in clinical health workers (CHW). All CHWs competent in climate, environmental change and health should know and understand the ways in which climate, environment and health intersect. To contribute to preparedness, response and recovery from adverse climate- and environment-related events, CHW also need to know and understand how climate, environmental change and health risks and impacts are managed, from local to global. CHW know what it means to be climate, environmental change and health competent; understand how diverse and multi-level health work is; competence occurs as workers gain experience in the world of work - supported by early training as well as continuous professional development - commensurate with their respective roles, duties and responsibilities. health in many different settings, from local to global. CHWs work at different levels, but all should be required to have some competences (knowledge, application and action) in intra-sectoral (e.g. within the health sector, public health, medical and emergency health services) and health-defining intersectoral services (e.g. environmental and social services supporting health). This means that competent health workers should be able to integrate complex climate, environmental change and health knowledge and skills about the environment, infrastructure, exposures, cultures, behaviors and health in many different settings, from local to global (Word Health Organization, 2018; International Council of Nurses, 2019; *Global Competency Framework for Universal Health Coverage*, 2022). | A six-domain competence: ‘know, apply, and take action’ according to the competence domains: Factual knowledge (known facts and understand them), gather evidence through research, tracking, monitoring, surveillance and be able to assess, be able to generate expert judgements and/or understand the evidence, advocating, collaborate, plan responses, access, engage and use aid and support processes, and know where and when to apply, Meaning, decision-making and action competences Integrating the competences = behavior as a process from novice to expert. Competence progression through learning (educational/training approach): Building practical knowledge and skill on top of educational foundation. Through continual learning eventually becoming an expert by gaining ‘knowing and understanding real life problems’ (from factual to practical knowledge) as well as ‘what to do about it’ (apply and act–which is the skill). The levels of competence depend development of cognitive abilities and executive behaviors. Initially during early training, the progresses start from factual knowledge to application (know what to do). Ultimately this leads to competent professional behavior demonstrated by the ability to act through making and implementing good decisions commensurate with their respective roles, duties and responsibilities. |
| **(Jochem et al., 2022)** | Planetary health literacy | Planetary health literacy is a holistic framework that combining elements from climate health literacy, ecological health literacy, environmental health literacy, and sustainable literacy. Planetary health literacy can be defined as the knowledge and competences of accessing, understanding, appraising, and applying information in order to make judgements and take decisions regarding planetary health, across societies and for health-promoting, sustainable, and transformative actions. Planetary health literate individuals and societies are enabled to sustain and promote their own health, population health, and the planet’s health. They are able to adopt a more holistic understanding of their health embedded in natural systems they are living in. Based on their knowledge and attitude, they take decisions that reflect and foster the interconnectedness of human health and well-being with the state of the natural systems and related areas of nature-society interactions. This conceptual model of planetary health literacy pursues a whole-of-society perspective that targets individuals of all age groups (irrespective of their personal and professional backgrounds) (Orr, 1989; Hollweg, K. S., Taylor, J. R., Bybee, R. W., Marcinkowski, T. J., McBeth, W. C., & Zoido, P, 2011; Goleman, 2012; Sørensen et al., 2012; Schneidewind, 2013; Whitmee et al., 2015; Decamps, 2017; Finn and O'Fallon, 2017; Limaye et al., 2020a; Nutbeam and Muscat, 2021). | Information, knowledge and competences regarding planetary health at individual, societal and global level in interconnection: The process of accessing, understanding, appraising, and applying knowledge and expertise on planetary health. Planetary health literacy goes beyond the individual health level across the life course, and includes the societal and global level across multiple generations. Planetary health-literate individuals and societies as the desired outcome of education for planetary health. |
| **(Jochem et al., 2024)** | Planetary health literacy | Planetary health literacy defining as the knowledge and competences of accessing, understanding, appraising, and applying information in order to make judgements and take decisions regarding planetary health, across societies and for health-promoting, sustainable, and transformative actions. Planetary health literate individuals and societies are enabled to sustain and promote their own health, population health, and the planet’s health. They are able to adopt a more holistic understanding of their health embedded in natural systems they are living in. Based on their knowledge and attitude, they take decisions that reflect and foster the interconnectedness of human health and wellbeing with the state of the natural systems and related areas of nature-society interactions. By encompassing both a life-course and transgenerational approach, planetary health literacy may act across multiple current and future generations. Thereby, planetary health literacy goes beyond the individual level and includes a societal and global level (Jochem et al., 2022). | See Jochem et al. (2023) and policy makers accelerate the implementation of broadly based educational programmers and health professionals perform planetary health literacy sensitive counselling and behavior within health systems. |
| **(Levett-Jones et al., 2024)** | Planetary health knowledges and skills | The three overarching domains of planetary health knowledges and skills are the science of planetary health and climate change, the Mitigation of the adverse impacts of healthcare on the environment and the Adaptation to the actual and expected impacts of climate change on healthcare and health outcomes .These domains provided a greater focus on climate change adaptation and mitigation, including sustainable practices, that are linked to nurses' scope of practice and actionable in any healthcare setting (Potter, 2019; Jochem et al., 2022). | Domains of planetary health knowledges and skills: Domain 1. The science of planetary health and climate change; Domain 2. Mitigation of the adverse impacts of healthcare on the environment, Domain 3. Adaptation to the actual and expected impacts of climate change on healthcare and health outcomes |
| **(Lichtveld et al., 2019)** | Environmental health literacy | Environmental health literacy refers to the understanding and use of information about potentially harmful environmental exposures and how those exposures influence health. Environmental health literacy encompasses a range of knowledge, attitudes, and behaviors that influence how individuals and communities use environmental information in making decisions about health. environmental health literacy is viewed as a dynamic process through which individuals and communities are increasingly able to apply a more nuanced understanding of environmental health risks, exposures, outcomes, and strategies to reduce adverse environmental exposures and promote health. Given the diversity of potential environmental exposures, it is also important to note that environmental health literacy is often specific to personal and community context and the contaminated environmental media involved. The term “environmental media” refers to parts of the environment, such as soil, water, air, plants and animals, that can contain contaminants, potentially resulting in individual or community exposure. Recently, the literature has emphasized the importance of information-seeking and decision-making skills that raise awareness and knowledge of environmental health and could result in health-protective actions. There is also recognition that the sources of environmental exposures are often outside an individual’s control and that health disparities can heighten community concerns. Furthermore, socioeconomic and contextual factors influence environmental health Literacy, including language, education, community networks, and news media. Accordingly, it is necessary to address environmental health concerns utilizing a culturally competent, interdisciplinary, and context-specific approach (Finn and O'Fallon, 2017; Gray, 2018; Hoover, 2019). | Knowledge was defined as facts and information pertaining to environmental health gained through experience or education. Attitudes were defined as settled ways of thinking or feeling about environmental health. Behaviors were defined as how individuals act in response to environmental health concerns. |
| **Limaye et al. (2020)** | Climate health literacy | Climate health–literate individual can recognize direct and indirect linkages between climate change and health, communicate risks, assess data, comprehend uncertainty, and make informed and responsible personal decisions or advocate for broader policies that protect health (U.S. Global change Research Program, 2009). | Climate health–literate individual recognizes linkages between climate change and health, communicates risks, assesses data, comprehends uncertainty, and make informed and responsible personal decisions or advocates for broader policies in the context of the Climate Literate Principles: Root Cause, Mechanism, Determinants, Implications, Interventions, Evidence, Complexity. At three levels of climate health literacy: functional, intermediate, advanced. |
| **(Lindsey et al., 2021)** | Environmental health literacy | Environmental health literacy combines elements from different disciplines, including health literacy, risk communication, environmental health, communications research, and safety culture. The basics of environmental health literacy start when an individual understands the link between environmental exposures and health outcomes. The process of becoming environmental health literate is defined as raising scientific literacy, environmental literacy, and numeracy; while increasing the awareness of specific exposures and their potential health effects, educational programs and community groups would benefit from a shared understanding of the skills and knowledge that underpin environmental health literacy (Massey, 2004; Sørensen et al., 2012; White et al., 2014; Finn and O'Fallon, 2017; Gray, 2018). | Individual understands the link between environmental exposures and health outcome, increasing the awareness of exposures, shared understanding of the skills and knowledge that underpin environmental health literacy. An environmental health-literate person is able to find info, to convey the concerns about environmental risk, to identify established hazards, to judge whether an information source is reliable. |
| **(Lopez-Medina et al., 2019)** | Environmental health competences | No definition. | Competences: communication, management and decision-making skills. critical reflection and thinking;  Knowledge and awareness: use interdisciplinary approaches to explore issues, identify, promote, own intelligence and philosophy of environmental sustainability and environment, understanding of Government policies and standards/targets, educate people to avoid impacts;  Skills; development and evaluation of interventions, detection of educational necessities and elaboration of educative programs, problem solving skills for adaptation to engage with complexity and uncertainty, disaster management skills  Behavior: interdisciplinary collaboration and also the need for sustainability behavior when not at work. |
| **(Lynch et al., 2024)** | Carbon health literacy | An individual’s ability to obtain, understand and evaluate the relevant information necessary to make decisions with an awareness of the likely consequences regarding greenhouse gas emissions (Howell, 2018; Irvin et al., 2019; Dósa and Russ, 2020). | Individual’s ability to obtain, understand and evaluate relevant information to make decisions with an awareness of the likely consequences. Demographic information (age, gender, years in practice, employment status, clinical specialty areas, country of training, and country of practice), views on the definition of carbon literacy, applies to healthcare context, use knowledge, share views on knowledge and skills. |
| **(Maxwell and Blashki, 2016)** | Eco-medical literacy | Eco-medical literacy as the ability to access, understand, integrate and use information about the health-related ecological effects of climate change to deliver and improve medical services. This can be further developed using eco-health perspectives. Eco-health approaches recognize the interdependence of human, animal and eco-system health and aim to develop transdisciplinary systems-focused collaboration to improve health through social stability and ecological sustainability. This broadest conceptualization of health, eco-health literacy, encompasses public health literacy (Waltner-Toews et al., 2004; Bell, 2010; Zinsstag, 2016). | Ability to access, understand, integrate and use information about the health-related ecological effects of climate change to deliver and improve medical services, needs factual, conceptual, skill-based, affective knowledge. |
| **(Moghbeli et al., 2024)** | Ecological care | Ecological care is a multidimensional integration of thinking, attitudes, awareness, sensitivity, and literacy to deliver high-quality holistic care while maintaining environmental sustainability and promoting energy conservation. Generally, Ecological Care can be defined as the integration of environmental sensitivity, knowledge, skills, attitudes, values, responsibilities, and active engagement, which enables nurses to make informed and responsible decisions to promote environmental sustain ability (Laustsen, 2006; Sattler and Hall, 2007; Dahlberg et al., 2016; Al-Shamaly, 2021). | Multidimensional integration of environmental thinking, understanding = (knowledge), awareness, sensitivity, skills, behavior, attitudes, religious values, responsibilities, consciousness, intelligence, sense of motivation and active engagement, which enables nurses to make informed and responsible decisions to promote environmental sustainability Interaction/ relationships, communication in an individual and professional approach. |
| **Raile (2023)** | Climate health literacy | Climate health literacy as the degree to which an individual understands the complex relationship between climate change and human health; a Climate health–literate individual can recognize direct and indirect linkages between climate change and health, communicate risks, assess data, comprehend uncertainty, and make informed and responsible personal decisions or advocate for broader policies that protect health (Limaye et al., 2020a; Grabow et al., 2023). | An individual understands the relationship between climate change and human health; can recognize linkages between climate change and health, communicate risks, assess data, comprehend uncertainty, and make informed and responsible personal decisions or advocate for broader policies. |
| **(Reismann et al., 2021)** | Climate-specific health literacy | Climate-specific health literacy entails:1) Knowledge about the present and long-term health risks of climate change.2) Knowledge about the health-co-benefits of preventative behaviors related to climate change. 3) Emotional integration of knowledge and feelings of concern related to climate change and health. 4) The ability of to implement this knowledge into action and climate-friendly behavior (Nutbeam, 2000; Whitmee et al., 2015). | Climate-specific health literacy entails: knowledge, emotional integration of knowledge and feelings of concern, the ability to implement this knowledge into action and climate-friendly behavior in seven climate-health literacy elements of functional, intermediate literacy, and advanced literacy. |
| **(Rosenau et al., 2024)** | Planetary health diet | No definition of planetary health diet literacy but a definition of planetary health literacy based on (Breidenassel C, Schäfer AC, Micka M, Richter M, Linseisen J, Watzl B, 2022) and (Willett et al., 2019). | Access/obtain, understand, appraise/judge information regarding planetary health and apply/use information relevant to planetary health. |

# **Additional Table 7: Cluster of determinants of the included competence and literacy concepts**

| **Cluster and explanation of determinants**  (in accordance to Sørensen et al., 2012) | **Key aspects included in the cluster of determinants** |
| --- | --- |
| **Knowledge** | Faculty knowledge, practical knowledge, environmental knowledge, skill-based knowledge, knowledge of fundamental climate science, knowledge of adaptation and mitigation strategies |
| **Competences, Skills, Abilities** | Sense of responsibility, critical reflection, critical reasoning, critical thinking, innovative problem-solving, flexible thinking, attention, experience, opinions, confidence, collaboration, post-disaster management, self-management, willingness for climate friendly behavior , emotional integration of knowledge and feelings, awareness, thinking, understanding, sensitivity, attitudes, consciousness, intelligence, motivation, active engagement, communication, clinical procedural and management skills emergency medical skills, leadership skills, advocacy skills, academic skills, transformative skills, transferable skills |
| **Competence Attributes** | engage, plan, collaborate, generate, track, monitor, gather evidence, describe, explain, analyze, evaluate, compare, articulate, integrate, make choices, seek out, comprehend, identify, convey, share, create, adopt, find, recognize, comprehend, advocate, access, understand, appraise, apply, use, integrate, deliver, improve, obtain, make decisions, applies, interact, promote, judge, take actions, assess, communicate |
| **Competence Dimensions** | Functional- critical - interactive  Functional – intermediate – advanced  From novice too expert:  Factual knowledge – meaning and decision-making – act  Access – understand – appraise – apply  Recognize – understand – apply – analyze – evaluate – create  No knowledge – partial knowledge – baseline knowledge – advanced knowledge  Factual knowledge – knowledge sufficiency – efficacy  Factual – conceptual – procedural knowledge  Factual knowledge – practical knowledge  Individual – societal – global level |
| **Actions and outcomes** | Implement knowledge into action and climate-friendly behavior.  Increasing the carbon handprint.  Reduce health risks.  Improve quality of life.  Protect the environment.  Justify health decisions.  Positive health outcomes.  Health protective actions.  Avoid contact with harmful environmental influences.  Prevention of environmental health risk.  Empower individuals and communities to experts.  Control over the environmental exposures that may lead to adverse health outcomes.  Enable individuals to develop and trust their intellectual competences and practical skillsets.  Transdisciplinary and systems-focused collaboration to improve health through social stability and ecological sustainability.  Shaping the health conditions of indigenous peoples, their health status and their social and emotional well-being.  Help local governments.  Support one’s decision to engage in protective behaviors.  Fight against pollution.  Deliver high-quality holistic care.  Deliver and improve medical services.  Apply a more nuanced understanding of Environmental Health risks, exposures, outcomes, and strategies to reduce adverse environmental exposures and promote health.  Mitigation of the adverse impacts of healthcare on the environment.  Adaptation to the actual and expected impacts of climate change on healthcare and health outcomes.  Policy makers accelerate the implementation of broadly based educational programs.  Perform Planetary Health Literacy sensitive counselling and behavior within health systems.  Make judgements and take decisions regarding Planetary Health, across societies and for health-promoting, sustainable, and transformative actions.  Sustain and promote the own health, population health, and the planet’s health.  Adopt a more holistic understanding of health embedded in natural systems.  Take decisions that reflect and foster the interconnectedness of human health and well-being with the state of the natural systems and related areas of nature-society interactions.  Integrate complex knowledge and skills about the environment, infrastructure, exposures, cultures, behaviors and health in many different settings, from local to global.  Improve quality of life.  Knows and understands the influence of own behavior or health facilities on the climate and the influence of the climate on themselves, on health facilities, the ecosystem and subsequently on society - especially in the area of physical, mental and social health.  Reducing greenhouse gas emissions.  Minimize the carbon footprint.  Make sustainable food choices. |
| **Context** | Clinical settings, different healthcare settings, health-related contexts, healthcare system, leadership, individual and community level, workplace, professional approach , interdisciplinary approaches, general public, health workers in science and governance, interactions among components of the earth system., interconnectedness and interdependence of human, animal and eco-system health, ecological care in nursing, emergency care, extended clinical practice, environmental health, planetary health, public health, population health, indigenous health, professional, legal and ethical practice, rural and remote context, holistic care, intersectoral (e.g. within the health sector, public health, medical and emergency health services), health-defining intersectoral services (e.g. environmental and social services supporting health). |
| **Development Process** | Across the life course, transgenerational, over lifetime, dynamic process, across multiple current and future generations, in everyday life |
| **Individual Antecedents**  **Contextual Antecedents** | Behavioral barriers, assumptions, respective roles of a person, gender, sex, ethnicity, race, social-economic status, views, values, beliefs, philosophy, political ideology, religiosity, trust in research, attention to news, academic performance, level of education, self-efficacy, rent home, income, health status & habits, age, cognitive abilities, intelligence, willingness, language |
|  | Structural barriers, respective roles of a person, location/ place of residence, education system, health system, policy makers, safety culture, specific environmental media, different languages, community networks, influence of media, governance, cultural context, technological and related regulatory developments, communication modalities, scientific enterprise, systems leadership, community networks, new media, institutional support |

# **Additional Table 8: Target groups applicability to health professionals**

| **First author, year** | **Target group including** | **Reason for applicability to health professionals (including trainees/students)** |
| --- | --- | --- |
| (Albrecht et al., 2023) | Health professionals (physicians and nurses) from various clinical specialties | *a) Can be directly transferred to the role of health professionals |
| (Atta et al., 2024) | Nursing students | a) Can be directly transferred to the role of health professionals |
| (Barna et al., 2020) | Health professional students (medical and nursing) | a) Can be directly transferred to the role of health professionals |
| (Baungaard et al., 2023) | Nutrition students | b) The application scenario is relevant to health professional practice: ‘Nutrition professionals are important stakeholders in sustainable food systems with skills to promote the connection between health, food production, environment, culture and economics.’ (Baungaard et al., 2023, p.572) |
| (Bell, 2010) | Health practitioners (especially rural and remote health practitioners) | a) Can be directly transferred to the role of health professionals |
| (Binder et al., 2022) | General adult population | b) The application scenario is relevant to health professional practice: ‘[…] Investigates the relationships between environmental health literacy, the characteristics of people (race, ethnicity, and socioeconomic status) associated with health disparities, and people’s willingness to engage in protective behaviors against environmental health threats.’ (Binder et al., 2022, p. 1) |
| (Blankart et al., 2024) | Health policy, governance, and leadership in One Health | b) The application scenario is relevant to health professional practice: ‘Improved One Health Literacy will then have an impact on policymakers resulting in changes in governance through enforcing pandemic preparedness plans, regulations for agriculture, veterinary and human medicine, public health interventions and enhanced health curricula/training’ (Blankart et al., 2024, p. 2) |
| (Brugger et al., 2024) | Health professionals | a) Can be directly transferred to the role of health professionals |
| (Brugger and Horváth, 2024) | Health professionals, students, practitioners, and educators | a) Can be directly transferred to the role of health professionals |
| (Davis et al., 2018) | Communities with known environmental health stressors in Arizona, USA | b) The application scenario is relevant to health professional practice: ‘As the mitigation or prevention of community-level environmental health risk often requires coordinated action, scholars have identified the need for Environmental Health Literacy to not only include knowledge and efficacy for personal environmental health actions, but also for community level action’ (Davis et al., 2018, p.2). |
| (Finn and O'Fallon, 2017) | Public health, EH scientists and community health educators | b) ‘Health care professionals are another stakeholder group that could benefit from targeted education and training to enable them to recognize symptoms caused by environmental exposures and to diagnose environmentally induced diseases. Conduct Environmental Health Literacy research’ (Finn and O'Fallon, 2017, p. 499). |
| (Fiore et al., 2024) | University students from different fields of specialisation | b) The application scenario is relevant to health professional practice: ‘The respondent students were equally distributed into courses belonging to scientific-health (e.g. biological and environmental sciences, medicine, pharmacy, mathematics, and engineering) and Humanistic-Legal-Social (sociology, political sciences, literature, philosophy, economics, and law) areas’ (Fiore et al., 2024, p. 2002). |
| (Grabow et al., 2023) | Learners: students, practicing professionals in the current formal workforce, and other interested individuals | a) Can be directly transferred to the role of health professionals : ‘Health professional students across subject matter areas could access Tier 3 content via formal professional development, such as the University of Colorado’s innovative Diploma in Climate Medicine, which trains students to develop leadership skills on climate and health science policy, workforce training, research, and linkages to environmental justice’ (Grabow et al., 2023, p. 04). |
| (Jacobsen et al., 2024) | Educators and health professional learners | a) Can be directly transferred to the role of health professionals |
| (Jagals and Ebi, 2021) | Health workforce, health/medical trainers | a) Can be directly transferred to the role of health professionals |
| (Jochem et al., 2022) | Specialists from the fields of public health, environmental science, education and policy-making | a) Can be directly transferred to the role of health professionals: ‘Current and future teachers and health professionals play a key role as “health literacy agents” and need to acquire planetary health literacy for themselves and the corresponding capability to provide planetary health literacy centered education or medical consultation. (Jochem et al., 2022, p. 08). |
| (Jochem et al., 2024) | Policy makers, health professionals, educators, general public | a) Can be directly transferred to the role of health professionals |
| (Levett-Jones et al., 2024) | Nursing students | a) Can be directly transferred to the role of health professionals |
| (Lichtveld et al., 2019) | Public health students, community members | a) Can be directly transferred to the role of health professionals: Public health workers, such as public health nurses, are part of the ISCO classification (World Health Organisation, 2019). |
| (Limaye et al., 2020a) | students, educators, health professionals and the broader public | a) Can be directly transferred to the role of health professionals |
| (Lindsey et al., 2021) | Environmental Health professionals, environmental public health educators, and users of Environmental Health information in the public | b) The application scenario is relevant to health professional practice: ‘This study aimed to identify knowledge and skill items identified by Environmental Health professionals as being associated with EHL, and understand how these items rank by importance (Lindsey et al., 2021, p. 4). |
| (Lopez-Medina et al., 2019) | Nursing students | a) Can be directly transferred to the role of health professionals |
| (Lynch et al., 2024) | Clinicians from speciality areas | a) Can be directly transferred to the role of health professionals |
| (Maxwell and Blashki, 2016) | Medical students and doctors | a) Can be directly transferred to the role of health professionals |
| (Moghbeli et al., 2024) | Nursing students and Nurses | a) Can be directly transferred to the role of health professionals |
| (Raile, 2023) | Psychotherapists | a) Can be directly transferred to the role of health professionals |
| (Reismann et al., 2021) | Patients of general practitioners and gynecologists | a) Can be directly transferred to the role of health professionals |
| (Rosenau et al., 2024) | University students | c) Professional competences are not explicitly excluded: ‘University students, in their role as potential future experts and leaders in their fields, can serve as important change agents of society.’ (Rosenau et al., 2024, p.2) |

# **Additional Table 9: Evidence-to-framework matrix of the PROF-PHL framework**

| **PROF-PHL Determinants in Figure 2** | **Identified Original Determinants**  **Reference**  **Original Concept** | **Statement of Integration**  (directly derived, adapted from theoretical framework, synthesized by the authors) | **Explanation of the Mode of Integration** |
| --- | --- | --- | --- |
| **Antecedents**  **Individual factors**  **Contextual factors** | Antecedents (personal characteristics and organizational policies)  (Moghbeli et al., 2024)  **Ecological care** | Adapted from theoretical framework | In their analysis of ecological care, Moghbeli et al. (2024) identified the ‘antecedents’ as important potential influences on health literacy. We have incorporated the concept of 'antecedents' into our model. From the sub-categories 'Personal Characteristics' and 'Organisational Policies', we have chosen the more general term 'Individual and Contextual Factors', as we believe it encompasses a wider range of potential influences on competence development. |
| **Antecedents**  **Individual factors: age and gender** | The demographic characteristics gender and age  (Albrecht et al., 2023)  **Climate-specific health literacy** | Adapted from theoretical framework | In their analysis of climate-specific health literacy, Albrecht et al. (2023) identified the aforementioned demographic factors as important potential influences on health literacy. We have renamed these factors 'antecedents', categorized them according to their origin (individual influencing factors) and adapted them in our PROF-PHL framework. The terms ‘age’ and ‘gender’ were thus assigned to ‘Individual Factors’ in our model and adopted by name. |
| **Antecedents**  **Individual factors: health status and habits, ethnicity**  **Contextual factors: demographic situation, culture, political forces, social Systems** | Geographic diversity, health status and habits, institutional policies or initiatives concerning climate change and health education  (Atta et al., 2024)  **Climate change literacy** | Adapted from theoretical framework | In their analysis of climate-change literacy, Atta et al. (2024) identified the aforementioned demographic factors as important potential influences on health literacy. We have renamed these factors 'antecedents', categorized them according to their origin (individual or contextual) and adapted them for use in our PROF-PHL framework. In our model, the geographical diversity identified by Atta et al. (2024) was used to derive demographic situation, culture (contextual factors) and ethnicity (individual factors), while institutional policies or initiatives relating to climate change and health education in the PROF-PHL were used to derive political forces and social systems (contextual factors). Health status and habits were incorporated directly into our model as individual factors. |
| **Antecedents**  **Individual factors: values, willingness, ethnicity** | Ethnicity, personal values and beliefs, people’s willingness to engage in protective behaviors against environmental health threats.  (Binder et al., 2022)  **Environmental health literacy** | Adapted from theoretical framework | In their analysis of environmental health literacy, Binder et al. (2022) identified the aforementioned demographic factors as important potential influences on health literacy. We have renamed these factors 'antecedents', categorised them according to their origin (individual or contextual) and adapted them for use in our PROF-PHL framework. Consequently, in our model, we classified the identified factors – “ethnicity, race, personal values and beliefs, and people’s willingness to take protective measures against environment-related health risks” (Binder et al. 2022) – as individual factors, abbreviated them and adopted their names (ethnicity, values, willingness). |
| **Antecedents**  **Individual factors: self-efficacy** | ‘[…] we define EHL here as: (1) environmental science knowledge and awareness related to the community’s specific environmental risks; (2) skills and self-efficacy […].’  (Davis et al., 2018)  **Environmental health literacy** | Adapted from theoretical framework | In their analysis of environmental health literacy, Davis et al. (2018) identified self-efficacy as very important potential influence on health literacy development. We have classified this influencing factor as an individual factor and identified it by name in our model. |
| **Planetary health knowledge** | Figure 1: Knowledge and competencies regarding planetary health  (Jochem et al. 2022)  **Planetary health literacy** | Adapted from theoretical framework | Jochem et al. (2022) regard knowledge of planetary health as a key prerequisite for accessing, understanding, evaluating and applying information relating to planetary health. As this aligns with the understanding of the frameworks planetary-health nexus, knowledge of planetary health has been incorporated as a central foundation for the development of PROF-PHL. |
|  | […] the concept of climate-specific health literacy encompasses knowledge about the present and long-term health risks of climate change and knowledge about the health co-benefits of health promoting behaviours related to climate change. Moreover, the concept includes emotional integration of knowledge and feelings of concern related to climate change and health and the ability to implement this knowledge into action and climate-friendly behaviour.  (Albrecht et al., 2023)  **Climate-specific health literacy** | Synthesized by the authors | Albrecht et al. (2023) investigated the knowledge of climate change and its health impacts amongst health professionals, as well as their climate-specific health literacy. They identified knowledge as a prerequisite for developing health literacy. Within our PROF-PHL concept, we have reformulated their climate-health nexus as a planetary-health nexus and integrated it as the basis for the development of PROF-PHL. |
|  | […] enhancing climate change literacy among  nursing students is paramount to building a more resilient healthcare system prepared to confront the complexities of a changing climate by equipping future healthcare  professionals with the knowledge and skills necessary to address emerging health challenges.  (Atta et al., 2024)  **Climate change literacy** | Synthesized by the authors | Atta et al. (2024) identified knowledge as a prerequisite for developing climate change literacy. ‘Based on these associations, it is recommended that nursing professionals be equipped with comprehensive knowledge of climate adaptation strategies to better advocate for and implement effective public health measures.’ (Atta et al. 2024, p. 2)  Within our PROF-PHL concept, we have reformulated their climate-health nexus as a planetary-health nexus and integrated it as the basis for the development of PROF-PHL. |
|  | Knowledge: Integrate planetary health concepts across the curriculum; identify how to prevent the harmful effects of global environmental changes in ways that integrate culturally relevant knowledge practices.  (Barna et al., 2020)  **Planetary health learning outcomes and competences** | Synthesized by the authors | Barna et al. (2020) regard knowledge of planetary health as one of three key principles in health profession education (HPE). We have taken this as an opportunity to integrate planetary health as a knowledge base into our PROF-PHL framework. |
|  | Figure 1: Knowledge and competencies regarding planetary health  (Jochem et al. 2022)  **Planetary health literacy** | Adapted from theoretical framework | Jochem et al. (2022) regard knowledge of planetary health as a key prerequisite for accessing, understanding, evaluating and applying information relating to planetary health. As this aligns with the understanding of the frameworks planetary-health nexus, knowledge of planetary health has been incorporated as a central foundation for the development of PROF-PHL. |
|  | Climate-specific health literacy entails:1) Knowledge about the present and long-term health risks of climate change.2) Knowledge about the health-co-benefits of preventative behaviours related to climate change. 3) Emotional integration of knowledge and feelings of concern related to climate change and health.  (Reismann et al., 2021)  **Climate-specific health literacy** | Synthesized by the authors | Reismann et al. (2021) investigated the knowledge of climate change and its health impacts amongst health professionals, as well as their climate-specific health literacy. They identified knowledge as a prerequisite for developing climate-specific health literacy. Within our PROF-PHL concept, we have reformulated their climate-health nexus as a planetary-health nexus and integrated it as the basis for the development of PROF-PHL. |
| **PROF-PHL Core Competences: access, understand, appraise, apply** | The ability to access, understand, integrate and use information about the health-related effects of climate change to deliver and improve medical services.  (Bell, 2010)  **Environmental literacy for climate change and eco-medical literacy** | Adapted from theoretical framework | Bell et al. (2010) describe their concept of health literacy in terms of the core competences of 'access', 'understand', 'integrate' and 'use'. According to Bloom's Taxonomy, these attributes of competences correspond to the same levels of competence as access – understand – appraise – apply (Bloom, 1956). The fact that these competence descriptors are also included in validated health literacy frameworks, such as those developed by Sorensen et al. (2012), provides further evidence of their relevance, and have therefore used in the PROF-PHL. Furthermore, as their understanding of competence and literacy is geared towards health professionals, it can be readily applied to our PROF-PHL framework. |
|  | […] to access, understand, appraise and apply all relevant information that are related to one health order to make judgments, take decisions […].  Brugger et al. (2024)  **Health-related climate literacy** | Directly derived | Brugger et al. (2024) use the same core competence terms to define their health-related climate literacy concept as those found in validated health literacy frameworks (e.g. Sorensen et al., 2012) and other frameworks relating to environmental health literacy (e.g. Jochem et al., 2022; Rosenau et al., 2024). This highlights the importance of these competences. Accordingly, we have adopted these terms directly for our own core competences within the PROF-PHL framework. Furthermore, as their understanding of competence and literacy is geared towards health professionals, it can be readily applied to our PROF-PHL framework. |
|  | […] competencies that people need in order to seek out, comprehend, evaluate, and use environmental health information to make informed choices, […].  (Finn and O'Fallon, 2017)  **Environmental health literacy** | Adapted from theoretical framework | Finn and O'Fallon (2017) describe their concept of environmental health literacy in terms of the core competences of 'seek out’, ' comprehend’, ‘evaluate’, and 'use'. According to Bloom's Taxonomy, these attributes of competences correspond to the same levels of competence as access – understand – appraise – apply (Bloom, 1956). The fact that these competence descriptors (access, understand, appraise, apply) are also included in validated health literacy frameworks, such as those developed by Sorensen et al. (2012), provides further evidence of their relevance, and have therefore used in the PROF-PHL. |
|  | Assess credible scientific information, communicate clearly, and make informed decisions related to behaviours that impact the climate,  (Grabow et al., 2023)  **Climate health literacy** | Adapted from theoretical framework | Grabow et al. (2023) describe their concept of climate health literacy in terms of the core competences of 'assess’, ' communicate’, and ‘make'. According to Bloom's Taxonomy, these attributes of competences correspond to the same levels of competence as access – appraise – apply (Bloom, 1956). The fact that these competence descriptors (access, appraise, apply) are also included in validated health literacy frameworks, such as those developed by Sorensen et al. (2012), provides further evidence of their relevance, and have therefore used in the PROF-PHL. |
|  | Figure 1: Access – Understand – Appraise – Apply  (Jochem et al., 2024)  **Planetary health literacy** | Directly derived | Jochem et al. (2022) describe 'access, understand, appraise and apply' as the core competences of Planetary Health Literacy. As our PROF-PHL framework also encompasses the planetary-health nexus, and as these core competences are reflected in many of the included studies, we have incorporated them directly into our framework. Furthermore, as their understanding of competence and literacy is geared towards health professionals, it can be readily applied to our PROF-PHL framework. |
|  | Climate health–literate individual recognizes linkages between climate change and health, communicates risks, assesses data, comprehends uncertainty, […]  Limaye et al. (2020)  **Climate heath literacy** | Adapted from theoretical framework | Limaye et al. (2020) describe their concept of climate health literacy in terms of the core competences of ‘assess’, 'recognize’, ' comprehend’ and 'communicate'. According to Bloom's Taxonomy, these attributes of competences correspond to the same levels of competence as access – understand – appraise – apply (Bloom, 1956). The fact that these competence descriptors (access, understand, appraise, apply) are also included in validated health literacy frameworks, such as those developed by Sorensen et al. (2012), provides further evidence of their relevance, and have therefore used in the PROF-PHL. Furthermore, as their understanding of competence and literacy is geared towards health professionals, it can be readily applied to our PROF-PHL framework. |
|  | An individual’s ability to obtain, understand and evaluate the relevant information necessary to make decisions […].  (Lynch et al., 2024)  **Carbon health literacy** | Adapted from theoretical framework | Lynch et al. (2024) describe their concept of carbon health literacy in terms of the core competences of 'obtain’, ' understand’, ‘evaluate’, and 'make'. According to Bloom's Taxonomy, these attributes of competences correspond to the same levels of competence as access – understand – appraise – apply (Bloom, 1956). The fact that these competence descriptors (access, understand, appraise, apply) are also included in validated health literacy frameworks, such as those developed by Sorensen et al. (2012), provides further evidence of their relevance, and have therefore used in the PROF-PHL. Furthermore, as their understanding of competence and literacy is geared towards health professionals, it can be readily applied to our PROF-PHL framework. |
|  | Eco-medical-literacy as the ability to access, understand, integrate and use information about the health-related ecological effects of climate change to deliver and improve medical services. (Maxwell and Blashki, 2016)  **Eco-medical-literacy** | Adapted from theoretical framework | Maxwell and Blashki (2016) describe their concept of eco-medical literacy in terms of the core competences of 'access’, ' understand’, ‘integrate’, and 'use'. According to Bloom's Taxonomy, these attributes of competences correspond to the same levels of competence as access – understand – appraise – apply (Bloom, 1956). The fact that these competence descriptors (access, understand, appraise, apply) are also included in validated health literacy frameworks, such as those developed by Sorensen et al. (2012), provides further evidence of their relevance, and have therefore used in the PROF-PHL. Furthermore, as their understanding of competence and literacy is geared towards health professionals, it can be readily applied to our PROF-PHL framework. |
|  | Access/obtain, understand, appraise/judge information regarding Planetary Health and apply/use information relevant to Planetary Health.  (Rosenau et al., 2024)  **Planetary health diet** | Adapted from theoretical framework | Rosenau et al. (2024) describe their concept of planetary health diet in terms of the core competences of 'access/obtain’, 'understand’, ‘appraise/judge’, and 'apply/use'. According to Bloom's Taxonomy, these attributes of competences correspond to the same levels of competence as access – understand – appraise – apply (Bloom, 1956). The fact that these competence descriptors (access, understand, appraise, apply) are also included in validated health literacy frameworks, such as those developed by Sorensen et al. (2012), provides further evidence of their relevance, and have therefore used in the PROF-PHL. Furthermore, the concept is shaped by the same planetary health nexus as the PROF-PHL framework, ensuring good transferability. |
| **Professional Actions and Outcomes:**  **Individual level, organizational level, policy level** | Figure 2.3: Levels of action: policy, healthcare system, individuum  (Brugger and Horváth, 2024)  **Health-related climate literacy** | Directly derived and adapted from theoretical framework | Figure 2.3, 'Levels of action and target groups for health-related climate literacy', and the accompanying explanation by Brugger and Horváth (2024), outline the levels of action for health professionals. These levels of action (individual, healthcare and policy) have been directly applied to our PROF-PHL framework, with 'healthcare' generalised as 'organisational level', as our study is aimed at the practice of health professionals, and these levels provide a useful classification of the key actions required. |
| **Continuum of life long, transgenerational and intersectoral learning** | ‘In order to take a life course and transgenerational approach,  lifelong learning should be an integral aspect of both education  for planetary health and planetary health literacy.’  (Jochem et al. 2022)  **Planetary Health Literacy** | Directly derived | The concept of competence development outlined by Jochem et al. (2022) aligns with well-established competence frameworks (World Health Organisation, 2022). Furthermore, it aligns with the environmental-health nexus examined in our study. The authors therefore deemed this approach suitable for direct incorporation into the PROF-PHL model. |

# **Additional Table 10: Distinct roles and levels of abstraction of the PROF-PH determinants.**

| **PROF-PHL Determinants** | **Distinct Roles and Levels of Abstraction** |
| --- | --- |
| **Antecedents (Individual and Contextual Factors)** | They refer to the factors that influence competence development (Lichtveld et al., 2019; Lindsey et al., 2021; Moghbeli et al., 2024). Individual antecedents (e.g. values, self-efficacy) (Moghbeli et al., 2024) are internal to the health professional, whereas contextual antecedents (e.g. social systems and political forces) are systemic and structural (Lichtveld et al., 2019; Lindsey et al., 2021). These all are enabling or constraining conditions, not competences themselves. |
| **PROF-PHL Knowledge** | Planetary Health Knowledge encompasses the understanding of the interdependence between human health and the health of Earth's natural systems. Core domains include Earth system changes, ecological dynamics, climate–health interactions, environmental justice, sustainable healthcare practices and the ethical dimensions of planetary stewardship (Jacobsen et al., 2024; Whitmee et al., 2015).  Within this framework, PROF-PHL Knowledge itself is not a competence, but rather the essential foundation upon which core competences are built (Jochem et al., 2022). It is a prerequisite for developing the four competence dimensions: access, understand, appraise and apply. PROF-PHL Knowledge occupies a distinct foundational level, serving as the resource that is transformed through competences into meaningful professional practice and organizational and policy impact (Jochem et al., 2024). Although individual and contextual factors (antecedents) influence the acquisition and application of knowledge, knowledge itself is the content that is accessed, understood, and applied. |
| **PROF-PHL Core Competences** | They represent integrated capabilities - the ability to access, understand, appraise and apply planetary health knowledge in professional contexts. Unlike isolated skills, competences combine knowledge, skills, and attitudes into actionable, context-sensitive abilities (The Council of the European Union, 2018). Attributes are measurable, observable indicators of competence development (Sørensen et al., 2012). They operationalize competences using specific verbs (e.g. ‘understand’, ‘appraise’) that are aligned with Bloom's Taxonomy (1956) and the Council of the European Union (2018) competence standards. Therefore, attributes determine how competences are demonstrated and form part of the formulation of competences. |
| **Professional Actions and Outcomes** | Professional actions are the visible manifestations of applied competences in practice (Jagals and Ebi, 2021), such as advocating for sustainable healthcare policies or integrating environmental risk assessments into clinical decisions. These are what professionals do, not the underlying capacity. Outcomes of these actions are their impacts, categorized by level: individual (e.g. improved professional planetary health knowledge), organizational (e.g. use greener clinical practices) and policy (e.g. policy engagement to reduced carbon footprint in healthcare)(Brugger and Horváth, 2024). Outcomes reflect the consequences of health professionals’ actions(Limaye et al., 2020a). |

# **Additional Figure 1: Attributes of the PROF-PHL core competence dimensions**

*The attributes are examples from the included studies

**REFERENCES**

Adisasmito, W. B., Almuhairi, S., Behravesh, C. B., Bilivogui, P., Bukachi, S. A., Casas, N., et al. (2022). One Health: A new definition for a sustainable and healthy future. *PLOS Pathogens* 18, e1010537. doi: 10.1371/journal.ppat.1010537

Albrecht, L., Reismann, L., Leitzmann, M., Bernardi, C., Sommoggy, J. von, Weber, A., et al. (2023). Climate-specific health literacy in health professionals: an exploratory study. *Front Med (Lausanne)* 10, 1236319. doi: 10.3389/fmed.2023.1236319

Al-Shamaly, H. S. (2021). A focused ethnography of the culture of inclusive caring practice in the intensive care unit. *Nurs Open* 8, 2973–2985. doi: 10.1002/nop2.1009

Atta, M. H. R., Zoromba, M. A., Asal, M. G. R., AbdELhay, E. S., Hendy, A., Sayed, M. A., et al. (2024). Predictors of climate change literacy in the era of global boiling: a cross-sectional survey of Egyptian nursing students. *BMC NURSING* 23, 676. doi: 10.1186/s12912-024-02315-y

Austrian Panel on Climate Change (2018). *Österreichischer Special Report Gesundheit, Demographie und Klimawandel (ASR18).* Wien.

Barna, S., Maric, F., Simons, J., Kumar, S., and Blankestijn, P. J. (2020). Education for the Anthropocene: Planetary health, sustainable health care, and the health workforce. *Med Teach* 42, 1091–1096. doi: 10.1080/0142159X.2020.1798914

Baungaard, C., Lane, K. E., and Richardson, L. (2023). Understanding nutrition students’ knowledge, perceived barriers and their views on the future role of nutritionists regarding sustainable diets. *Nutrition Bulletin* 48, 572–586. doi: 10.1111/nbu.12649

Bell, E. J. (2010). Climate change: what competencies and which medical education and training approaches? *BMC medical education* 10, 31. doi: 10.1186/1472-6920-10-31

Binder, A. R., May, K., Murphy, J., Gross, A., and Carlsten, E. (2022). Environmental Health Literacy as Knowing, Feeling, and Believing: Analyzing Linkages between Race, Ethnicity, and Socioeconomic Status and Willingness to Engage in Protective Behaviors against Health Threats. *Int J Environ Res Public Health* 19. doi: 10.3390/ijerph19052701

Blankart, C. R., Gani, S. M. de, Crimlisk, H., Desmedt, M., Bauer, B., and Doyle, G. (2024). Health literacy, governance and systems leadership contribute to the implementation of the One Health approach: a virtuous circle. *Health Policy* 143, 105042. doi: 10.1016/j.healthpol.2024.105042

Breidenassel C, Schäfer AC, Micka M, Richter M, Linseisen J, Watzl B (2022). The Planetary Health Diet in contrast to the food-based dietary guidelines of the German Nutrition Society (DGE). *Ernährungs Umschau*, 56–72.e1–3.

Brent Kim, Roni Neff, Raychel Santo, and Juliana Vigorito (2015). *The Importance of Reducing Animal Product Consumption and Wasted Food in Mitigating Catastrophic Climate Change*. Unpublished.

Brugger, K., Horvath, I., Schmidt, A. E., and Marent, J. (2024). National activities to strengthen climate literacy in healthcare professions in Austria. *PRAVENTION UND GESUNDHEITSFORDERUNG*. doi: 10.1007/s11553-024-01169-1

Brugger, K., and Horváth, I. (2024). *Klimakompetenz von Angehörigen der Gesundheitsberufe: Ergebnisbericht*. Wien.

*Classifying health workers: mapping occupations to the international standard classification (ISCO)* (2019).

*Climate Change and Health: A Public Health Education Toolkit* (2022).

*Core Competencies in disaster nursing* (2019). Geneva.

Crimmins, A., Balbus, J., Gamble, J. L., Beard, C. B., Bell, J. E., Dodgen, D., et al. (2016). *The Impacts of Climate Change on Human Health in the United States: A Scientific Assessment*. U.S. Global Change Research Program.

Dahlberg, H., Ranheim, A., and Dahlberg, K. (2016). Ecological caring-Revisiting the original ideas of caring science. *Int J Qual Stud Health Well-being* 11, 33344. doi: 10.3402/qhw.v11.33344

Davis, L. F., Ramirez-Andreotta, M. D., McLain, J. E. T., Kilungo, A., Abrell, L., and Buxner, S. (2018). Increasing Environmental Health Literacy through Contextual Learning in Communities at Risk. *Int J Environ Res Public Health* 15. doi: 10.3390/ijerph15102203

Decamps, A. (2017). *Analysis of Determinants of a Measure of Sustainability Literacy: Global Education Monitoring (GEM) Report, Accountability in Education: Meeting our Commitments*.

Décamps, A., Barbat, G., Carteron, J.-C., Hands, V., and Parkes, C. (2017). Sulitest: A collaborative initiative to support and assess sustainability literacy in higher education. *The International Journal of Management Education* 15, 138–152. doi: 10.1016/j.ijme.2017.02.006

Dixon, J. K., Hendrickson, K. C., Ercolano, E., Quackenbush, R., and Dixon, J. P. (2009). The environmental health engagement profile: what people think and do about environmental health. *Public Health Nurs* 26, 460–473. doi: 10.1111/j.1525-1446.2009.00804.x

Dósa, K., and Russ, R. S. (2020). Making sense of carbon footprints: how carbon literacy and quantitative literacy affects information gathering and decision-making. *Environmental Education Research* 26, 421–453. doi: 10.1080/13504622.2019.1569205

Ergin, E., Altinel, B., and Aktas, E. (2021). A mixed method study on global warming, climate change and the role of public health nurses from the perspective of nursing students. *Nurse Educ Today* 107, 105144. doi: 10.1016/j.nedt.2021.105144

Estacio, E. V. (2013). Health literacy and community empowerment: it is more than just reading, writing and counting. *J Health Psychol* 18, 1056–1068. doi: 10.1177/1359105312470126

Finn, S., and O'Fallon, L. (2017). The Emergence of Environmental Health Literacy-From Its Roots to Its Future Potential. *Environ Health Perspect* 125, 495–501. doi: 10.1289/ehp.1409337

Fiore, M., Lorini, C., Bonaccorsi, G., Paoli, S., Vaccaro, G., Verani, M., et al. (2024). Development and validation of the Environmental Health Literacy Index: a new tool to assess the environmental health literacy among university students. *EUROPEAN JOURNAL OF PUBLIC HEALTH* 34, 1001–1007. doi: 10.1093/eurpub/ckae120

Gani, S. de, Schaeffer, D., Griebler, R., Jaks, R., Haarmann, A., Griese, L., et al. (2023). Professional health literacy - first results of a pilot study in Austria, Germany, and Switzerland. *Popul. Med.* 5. doi: 10.18332/popmed/164043

Gerber, P.J., Steinfeld, H., Henderson, B., Mottet, A., Opio, C., Dijkman, J., Falcucci, A. & Tempio, G. (2013). *Tackling climate change through livestock – A global assessment of emissions and mitigationopportunities.* Rome.

*Gesundheitsziel 4: Luft, Wasser, Boden und alle Lebensräume für künftige Generationen sichern - Bericht der Arbeitsgruppe* (2019). Wien.

*Global Competency Framework for Universal Health Coverage* (2022). Geneva: World Health Organization.

Goleman, D. (2012). *Ecoliterate: How Educators Are Cultivating Emotional, Social, and Ecological Intelligence*. Somerset: John Wiley & Sons Incorporated.

Grabow, M. L., Stull, V. J., Hahn, M. B., and Limaye, V. S. (2023). A blueprint for strengthening climate and health literacy through professional adaptability. *Front Public Health* 11, 1112944. doi: 10.3389/fpubh.2023.1112944

Gray, K. M. (2018). From Content Knowledge to Community Change: A Review of Representations of Environmental Health Literacy. *Int J Environ Res Public Health* 15. doi: 10.3390/ijerph15030466

Gray, K. M., and Lindsey, M. (2019). “Measuring Environmental Health Literacy,” in *Environmental Health Literacy*, eds. S. Finn, and L. O'Fallon (Cham: Springer International Publishing), 19–43.

Gray, K. M., Triana, V., Lindsey, M., Richmond, B., Hoover, A. G., and Wiesen, C. (2021). Knowledge and Beliefs Associated with Environmental Health Literacy: A Case Study Focused on Toxic Metals Contamination of Well Water. *Int J Environ Res Public Health* 18. doi: 10.3390/ijerph18179298

Guzmán, C. A. F., Aguirre, A. A., Astle, B., Barros, E., Bayles, B., Chimbari, M., et al. (2021). A framework to guide planetary health education. *The Lancet Planetary Health* 5, e253-e255. doi: 10.1016/S2542-5196(21)00110-8

Hollweg, K. S., Taylor, J. R., Bybee, R. W., Marcinkowski, T. J., McBeth, W. C., & Zoido, P (2011). *Developing a framework for assessing environmental literacy*. Washington DC.

Hoover, A. G. (2019). “Defining Environmental Health Literacy,” in *Environmental Health Literacy*, eds. S. Finn, and L. R. O'Fallon (Cham: Springer International Publishing), 3–18.

Howell, R. A. (2018). Carbon management at the household level: a definition of carbon literacy and three mechanisms that increase it. *Carbon Management* 9, 25–35. doi: 10.1080/17583004.2017.1409045

Innes, S., Shephard, K., Furnari, M., Harraway, J., Jowett, T., Lovelock, B., et al. (2018). Greening the Curriculum to Foster Environmental Literacy in Tertiary Students Studying Human Nutrition. *Journal of Hunger & Environmental Nutrition* 13, 192–204. doi: 10.1080/19320248.2016.1255693

Irvin, V. L., Rohlman, D., Vaughan, A., Amantia, R., Berlin, C., and Kile, M. L. (2019). Development and Validation of an Environmental Health Literacy Assessment Screening Tool for Domestic Well Owners: The Water Environmental Literacy Level Scale (WELLS). *Int J Environ Res Public Health* 16. doi: 10.3390/ijerph16050881

Jacobsen, K. H., Waggett, C. E., Berenbaum, P., Bayles, B. R., Carlson, G. L., English, R., et al. (2024). Planetary health learning objectives: foundational knowledge for global health education in an era of climate change. *The Lancet Planetary Health* 8, e706-e713. doi: 10.1016/S2542-5196(24)00167-0

Jagals, P., and Ebi, K. (2021). Core Competencies for Health Workers to Deal with Climate and Environmental Change. *Int J Environ Res Public Health* 18. doi: 10.3390/ijerph18083849

Jochem, C., Sommoggy, J. von, Hornidge, A.-K., Schwienhorst-Stich, E.-M., and Apfelbacher, C. (2022). Planetary health literacy: A conceptual model. *Front Public Health* 10, 980779. doi: 10.3389/fpubh.2022.980779

Jochem, C., Sommoggy, J. von, Hornidge, A.-K., Schwienhorst-Stich, E.-M., and Apfelbacher, C. (2024). Planetary health literacy as an educational goal contributing to healthy living on a healthy planet. *Front Med (Lausanne)* 11, 1464878. doi: 10.3389/fmed.2024.1464878

Karesh, W. B., and Cook, R. A. (2009). One world--one health. *Clin Med (Lond)* 9, 259–260. doi: 10.7861/clinmedicine.9-3-259

Laustsen, G. (2006). Environment, ecosystems, and ecological behavior: a dialogue toward developing nursing ecological theory. *ANS Adv Nurs Sci* 29, 43–54. doi: 10.1097/00012272-200601000-00005

Levett-Jones, T., Catling, C., Cheer, S., Fields, L., Foster, A., Maguire, J., et al. (2024). Achieving consensus on the essential knowledge and skills needed by nursing students to promote planetary health and sustainable healthcare: A Delphi study. *JOURNAL OF ADVANCED NURSING*. doi: 10.1111/jan.16229

Lichtveld, M. Y., Covert, H. H., Sherman, M., Shankar, A., Wickliffe, J. K., and Alcala, C. S. (2019). Advancing Environmental Health Literacy: Validated Scales of General Environmental Health and Environmental Media-Specific Knowledge, Attitudes and Behaviors. *Int J Environ Res Public Health* 16. doi: 10.3390/ijerph16214157

Limaye, V. S., Grabow, M. L., Stull, V. J., and Patz, J. A. (2020a). Developing A Definition Of Climate And Health Literacy. *Health affairs (Project Hope)* 39, 2182–2188. doi: 10.1377/hlthaff.2020.01116

Limaye, V. S., Max, W., Constible, J., and Knowlton, K. (2019). Estimating the Health-Related Costs of 10 Climate-Sensitive U.S. Events During 2012. *Geohealth* 3, 245–265. doi: 10.1029/2019GH000202

Limaye, V. S., Max, W., Constible, J., and Knowlton, K. (2020b). Estimating The Costs Of Inaction And The Economic Benefits Of Addressing The Health Harms Of Climate Change. *Health affairs (Project Hope)* 39, 2098–2104. doi: 10.1377/hlthaff.2020.01109

Lindsey, M., Chen, S.-R., Ben, R., Manoogian, M., and Spradlin, J. (2021). Defining Environmental Health Literacy. *Int J Environ Res Public Health* 18. doi: 10.3390/ijerph182111626

Lopez-Medina, I. M., Alvarez-Nieto, C., Grose, J., Elsbernd, A., Huss, N., Huynen, M., et al. (2019). Competencies on environmental health and pedagogical approaches in the nursing curriculum: A systematic review of the literature. *NURSE EDUCATION IN PRACTICE* 37, 1–8. doi: 10.1016/j.nepr.2019.04.004

Lynch, M., Mccaffery, K., Barratt, A., Bell, K., Miller, F. A., Mcgain, F., et al. (2024). Australian and Canadian clinicians’ views and application of ’carbon health literacy’: a qualitative study. *BMC HEALTH SERVICES RESEARCH* 24. doi: 10.1186/s12913-024-11903-2

Macdiarmid, J. I. (2013). Is a healthy diet an environmentally sustainable diet? *Proc Nutr Soc* 72, 13–20. doi: 10.1017/S0029665112002893

Massey, R. (2004). *Environmental Justice: Income, Race, and Health*. Medford, U.S.

Maxwell, J., and Blashki, G. (2016). Teaching About Climate Change in Medical Education: An Opportunity. *Journal of public health research* 5, 673. doi: 10.4081/jphr.2016.673

Minkler, M., Vásquez, V. B., Tajik, M., and Petersen, D. (2008). Promoting environmental justice through community-based participatory research: the role of community and partnership capacity. *Health Educ Behav* 35, 119–137. doi: 10.1177/1090198106287692

Mogford, E., Gould, L., and Devoght, A. (2011). Teaching critical health literacy in the US as a means to action on the social determinants of health. *Health Promot Int* 26, 4–13. doi: 10.1093/heapro/daq049

Moghbeli, G., Soheili, A., Ghafourifard, M., Shahbazi, S., and Karkan, H. A. (2024). Ecological care in nursing practice: a Walker and Avant concept analysis. *BMC NURSING* 23, 1–10. doi: 10.1186/s12912-024-02279-z

Nutbeam, D. (2000). Health literacy as a public health goal: a challenge for contemporary health education and communication strategies into the 21st century. *Health Promot Int* 15, 259–267. doi: 10.1093/heapro/15.3.259

Nutbeam, D., and Muscat, D. M. (2021). Health Promotion Glossary 2021. *Health Promot Int* 36, 1578–1598. doi: 10.1093/heapro/daaa157

Orr, D. W. (1989). Ecological literacy. *Conserv Biol* 3, 334–335. doi: 10.1111/j.1523-1739.1989.tb00238.x

Page, M. J., McKenzie, J. E., Bossuyt, P. M., Boutron, I., Hoffmann, T. C., Mulrow, C. D., et al. (2021). The PRISMA 2020 statement: an updated guideline for reporting systematic reviews. *BMJ* 372, n71. doi: 10.1136/bmj.n71

Peters, M. D. J., Godfrey, C., McInerney, P., Munn, Z., Tricco, A. C., and Khalil, H. (2024). “Scoping reviews,” in *JBI Manual for Evidence Synthesis*, eds. E. Aromataris, C. Lockwood, K. Porritt, B. Pilla, and Z. Jordan (JBI).

*Planetary Health: Roadmap and Action Plan* (2024).

Potter, T. (2019). Planetary Health: The Next Frontier in Nursing Education. *Creat Nurs* 25, 201–207. doi: 10.1891/1078-4535.25.3.201

*Primary Curriculum for Rural and Remote Medicine* (2007).

Raile, P. (2023). The importance of teaching climate-health literacy in psychotherapeutic training and continuing education. *F1000Res* 12, 982. doi: 10.12688/f1000research.139879.2

Ramirez-Andreotta, M. D., Brody, J. G., Lothrop, N., Loh, M., Beamer, P. I., and Brown, P. (2016). Improving Environmental Health Literacy and Justice through Environmental Exposure Results Communication. *Int J Environ Res Public Health* 13. doi: 10.3390/ijerph13070690

Redvers, N., Faerron Guzmán, C. A., and Parkes, M. W. (2023). Towards an educational praxis for planetary health: a call for transformative, inclusive, and integrative approaches for learning and relearning in the Anthropocene. *The Lancet Planetary Health* 7, e77-e85. doi: 10.1016/S2542-5196(22)00332-1

Reismann, L., Weber, A., Leitzmann, M., and Jochem, C. (2021). Climate-specific health literacy and medical advice: The potential for health co-benefits and climate change mitigation. An exploratory study. *The Journal of Climate Change and Health* 4, 100072. doi: 10.1016/j.joclim.2021.100072

Romanello, M., Di Napoli, C., Green, C., Kennard, H., Lampard, P., Scamman, D., et al. (2023). The 2023 report of the Lancet Countdown on health and climate change: the imperative for a health-centred response in a world facing irreversible harms. *Lancet* 402, 2346–2394. doi: 10.1016/S0140-6736(23)01859-7

Rosenau, N., Neumann, U., Hamblett, S., and Ellrott, T. (2024). University Students as Change Agents for Health and Sustainability: A Pilot Study on the Effects of a Teaching Kitchen-Based Planetary Health Diet Curriculum. *Nutrients* 16. doi: 10.3390/nu16040521

Sattler, B., and Hall, K. (2007). Healthy choices: transforming our hospitals into environmentally healthy and safe places. *Online J Issues Nurs* 12, 3.

Schneidewind, U. (2013). Transformative Literacy Gesellschaftliche Veränderungsprozesse verstehen und gestaltenTransformative Literacy. Understanding and Shaping Societal Transformations. *GAIA - Ecological Perspectives for Science and Society* 22, 82–86. doi: 10.14512/gaia.22.2.5

Sorensen, C., Campbell, H., Depoux, A., Finkel, M., Gilden, R., Hadley, K., et al. (2023). Core competencies to prepare health professionals to respond to the climate crisis. *PLOS Clim* 2, e0000230. doi: 10.1371/journal.pclm.0000230

Sørensen, K., van den Broucke, S., Fullam, J., Doyle, G., Pelikan, J., Slonska, Z., et al. (2012). Health literacy and public health: a systematic review and integration of definitions and models. *BMC Public Health* 12, 80. doi: 10.1186/1471-2458-12-80

Springmann, M., Wiebe, K., Mason-D'Croz, D., Sulser, T. B., Rayner, M., and Scarborough, P. (2018). Health and nutritional aspects of sustainable diet strategies and their association with environmental impacts: a global modelling analysis with country-level detail. *Lancet Planet Health* 2, e451-e461. doi: 10.1016/S2542-5196(18)30206-7

Stone, S. B., Myers, S. S., and Golden, C. D. (2018). Cross-cutting principles for planetary health education. *The Lancet Planetary Health* 2, e192-e193. doi: 10.1016/S2542-5196(18)30022-6

The Council of the European Union (2018). Council Recommendation of 22 May 2018 on key competences for lifelong learning. *Official Journal of the European Union*.

Tricco, A. C., Lillie, E., Zarin, W., O'Brien, K. K., Colquhoun, H., Levac, D., et al. (2018). PRISMA Extension for Scoping Reviews (PRISMA-ScR): Checklist and Explanation. *Ann Intern Med* 169, 467–473. doi: 10.7326/M18-0850

U.S. Global change Research Program (2009). *Climate Literacy: The Essential Principles of Climate Science*.

Walpole, S. C., Mortimer, F., Inman, A., Braithwaite, I., and Thompson, T. (2015). Exploring emerging learning needs: a UK-wide consultation on environmental sustainability learning objectives for medical education. *Int J Med Educ* 6, 191–200. doi: 10.5116/ijme.5643.62cd

Waltner-Toews, D., Wilcox, B. A., Aguirre, A. A., Daszak, P., Horwitz, P., Martens, P., et al. (2004). EcoHealth: A Transdisciplinary Imperative for a Sustainable Future. *EcoHealth* 1, 3–5. doi: 10.1007/s10393-004-0014-9

White, B. M., Hall, E. S., and Johnson, C. (2014). Environmental health literacy in support of social action: an environmental justice perspective. *J Environ Health* 77, 24–29.

Whitmee, S., Haines, A., Beyrer, C., Boltz, F., Capon, A. G., Souza Dias, B. F. de, et al. (2015). Safeguarding human health in the Anthropocene epoch: report of The Rockefeller Foundation-Lancet Commission on planetary health. *Lancet* 386, 1973–2028. doi: 10.1016/S0140-6736(15)60901-1

*WHO competency framework for health workers’ education and training on antimicrobial resistance* (2018). Geneva.

Willett, W., Rockström, J., Loken, B., Springmann, M., Lang, T., Vermeulen, S., et al. (2019). Food in the Anthropocene: the EAT-Lancet Commission on healthy diets from sustainable food systems. *Lancet* 393, 447–492. doi: 10.1016/S0140-6736(18)31788-4

World Health Organisation (2022). *Global Competency Framework for Universal Health Coverage*. Geneva: World Health Organization.

Zarcadoolas, C., Timm, E., and Bibeault, L. (2001). Brownfields: a case study in partnering with residents to develop an easy-to-read print guide. *J Environ Health* 64, 15–20.

Zinsstag, J. (2016). One Health: EcoHealth 2016: Welcome from the President of the International Association for Ecology and Health. *EcoHealth* 13, 613–614. doi: 10.1007/s10393-016-1180-2

Zoller, H. M. (2012). Communicating Health: Political Risk Narratives in an Environmental Health Campaign. *Journal of Applied Communication Research* 40, 20–43. doi: 10.1080/00909882.2011.634816
